# PRISM-GEP: Viewing single-cell expression through the lens of topic modeling

**DOI:** 10.1093/bioinformatics/btag728

**Published:** 2026-09-29

**Authors:** Yanir Buznah, Tal Ishon, Uri Shaham

**Affiliations:** Department of Computer Science, Bar-Ilan University, Ramat Gan, Israel; Department of Computer Science, Bar-Ilan University, Ramat Gan, Israel; Department of Computer Science, Bar-Ilan University, Ramat Gan, Israel

## Abstract

**Motivation:**

Gene-expression programs (GEPs) are the co-regulated gene modules whose coordinated activity encodes biological processes. Recovering them from single-cell RNA-seq is hard because regulation is many-to-many. A cell can run several programs at once, and a gene can take part in several. Methods that resolve programs along a trajectory break this structure by assigning each gene to a single program.

**Results:**

PRISM-GEP discovers overlapping GEPs with Latent Dirichlet Allocation, giving it a prior estimated from gene co-expression in place of the flat default it normally assumes. No reference dataset or pathway database is required. Across 15 human and mouse datasets, PRISM-GEP matches specialized factorizations such as cNMF and scHPF on Gene Ontology Biological Process metrics. Over the 43 rankable dataset-by-metric entries, its mean rank is level with the best, and unlike every specialized method it never ranks last. The same co-expression geometry orders the genes within each program, recovering developmental cascades on par with GeneTrajectory.

**Availability and implementation:**

The implementation of our methods, including all code, datasets, and experimental workflows, is available in Python at https://github.com/shaham-lab/PRISM-GEP. The version used in this paper is archived at https://doi.org/10.5281/zenodo.22659056.

## 1 Introduction

Single-cell RNA-sequencing (scRNA-seq) has transformed our ability to dissect gene-expression programs (GEPs): the modular, co-regulated gene sets whose coordinated activity encodes biological processes, cell states, and perturbation responses. Because genes act in concert rather than in isolation (Eisen *et al.* 1998; Segal *et al.* 2003), delineating these combinatorial patterns is central to understanding differentiation, disease progression, and therapeutic response.

Recovering coherent GEPs is difficult because regulation is inherently many-to-many: a cell co-activates several programs, and a gene participates in several depending on cell type, microenvironment, and perturbation (He and Zhang, 2006; Zhang *et al.* 2022). Most established methods break this structure. Trajectory approaches that order cells along a latent pseudotime assume a single dominant process per cell (Trapnell *et al.* 2014; Wolf *et al.* 2019; Lange *et al.* 2022), so co-occurring processes such as cell-cycle progression and differentiation blur together. The gene-trajectory framework of Qu *et al.* (2025) instead lets each cell express multiple programs but assigns every gene to a single one, which again conflicts with pleiotropy. Both families thus risk composite or artificially partitioned programs that miss the overlapping, modular architecture of regulation.

Topic modeling is a natural fit for this many-to-many structure. Latent Dirichlet Allocation (LDA) (Blei *et al.* 2003) represents each document as a mixture over latent topics and each topic as a distribution over words. Mapping documents to cells, words to genes, and topics to programs, it grants genes and cells probabilistic, overlapping membership rather than hard partitions (developed in Section 4.1). Applied off the shelf, however, LDA draws its topics from a flat, uninformative prior (the assumption the model makes about each topic before it sees the data), which ignores the co-expression structure of the data. Moreover, topic modeling has not been systematically evaluated as a GEP-discovery method against the specialized factorizations used in practice.

We address both gaps with PRISM-GEP (PRIor from scRNA-seq Statistics for Modeling Gene-Expression Programs) (Fig. 1). PRISM-GEP replaces LDA’s flat prior with a data-derived one estimated from the dataset’s own gene–gene co-expression, grounding each program in the intrinsic structure of the data rather than a uniform default. The prior needs no external database or reference dataset, which distinguishes it from priors transferred from a reference fit (Min *et al.* 2023) or read from curated pathway databases (Zhao *et al.* 2021). It initializes LDA without altering the generative process, so the fitted model remains a standard, interpretable topic model. Its construction is detailed in the Proposed Method section.

**Figure 1:**
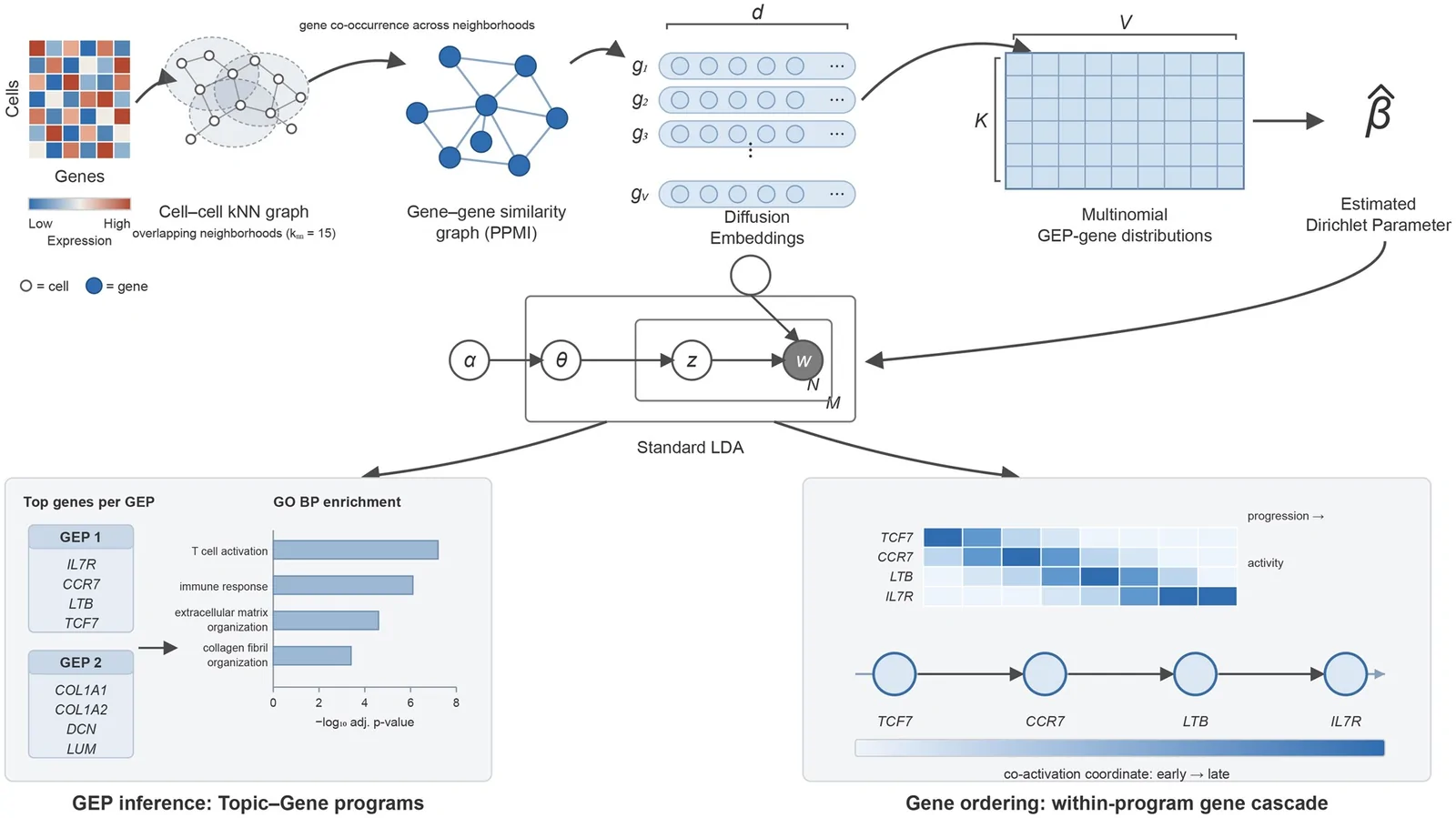
Overview of PRISM-GEP. From a gene-expression matrix we measure which genes are consistently co-expressed and use that structure to give a standard topic model (LDA) an informed, data-derived starting point, without changing its generative process (method details in Section 4). The fitted model supports (left) gene-program (GEP) inference with Gene Ontology enrichment and (right) an ordering of the genes within each program along a co-activation trajectory.

This work makes three contributions. First, we cast gene-program discovery as topic modeling with a data-derived co-expression prior, an interpretable framework that grounds each program in the dataset’s own gene co-expression. Second, we find this approach robust and competitive with specialized single-cell factorizations on Gene Ontology Biological Process quality across diverse datasets, with robustness as its distinguishing property. Third, the same gene–gene geometry yields an intrinsic within-program gene ordering that reproduces canonical marker cascades, competitive with the dedicated GeneTrajectory method (Qu *et al.* 2025).

The informed prior recovers the standard model’s performance at no systematic cost. An informed prior matters most when the data alone are only weakly informative, so any benefit is most visible where co-expression is focused rather than uniform. Together, the prior, the systematic benchmark, and the trajectory extension establish topic modeling as a robust, interpretable, and extensible framework for discovering and ordering GEPs.

## 2 Related work

A substantial literature addresses the recovery of GEPs from scRNA-seq. We group prior work into three families: (i) clustering approaches, (ii) trajectory-based methods (with cell- and gene-trajectory subfamilies), and (iii) scRNA-specific probabilistic models. We outline representative methods in each family, emphasizing modeling assumptions, strengths, and limitations for inferring overlapping, context-dependent GEPs.

### 2.1 Clustering methods

Direct clustering (*k*-means, Gaussian Mixture Models, or graph-based community detection such as Louvain/Leiden (Traag *et al.* 2019), typically after dimensionality reduction or *k*NN-graph construction) is widely used for coarse stratification of scRNA-seq. These methods optimize geometric separation of cells rather than an explicit generative model of programs and are sensitive to dropout, batch effects, feature choice, and resolution, leading to over/under-partitioning (Duò *et al.* 2018; Kiselev *et al.* 2019; Luecken and Theis, 2019). Even soft assignments (e.g., GMM) yield cell partitions, not the many-to-many relationships whereby cells co-activate multiple programs and genes contribute to several. We therefore treat clustering as a baseline rather than a primary mechanism for recovering multiplexed GEPs.

### 2.2 Trajectory-based methods

Trajectory-based approaches instead capture continuous dynamics, most following a two-step scheme: (1) infer gene programs and (2) order them along a latent trajectory. We distinguish *cell-trajectory* methods (ordering cells in latent space) from *gene-trajectory* methods (ordering genes in gene space). PRISM-GEP addresses both steps: it discovers overlapping programs (Step 1) and, from the same construction, orders the genes within each program (Step 2). We benchmark both against the methods below.

#### 2.2.1 Cell-trajectory

Cell-trajectory methods embed cells in a latent space and arrange them along smooth pseudotime paths that capture coordinated expression changes. *Monocle* established the idea of ordering cells to recover differentiation cascades (Trapnell *et al.* 2014). Later methods stabilize the latent manifold: diffusion pseudotime (DPT) propagates local structure along branches (Haghverdi *et al.* 2016), *Slingshot* fits simultaneous lineages through cluster centers (Street *et al.* 2018), and *PAGA* coarse-grains the *k*NN graph into a topology-preserving map (Wolf *et al.* 2019). *Palantir* instead quantifies fate probabilities and plasticity (Setty *et al.* 2019), and velocity-augmented approaches such as *CellRank* (Lange *et al.* 2022) use RNA velocity (La Manno *et al.* 2018) to orient state transitions. These tools reconstruct lineages and branch points but assume a single dominant process per cell, so concurrent processes are blurred by a one-dimensional pseudotime (Qu *et al.* 2025).

#### 2.2.2 Gene-trajectory

The gene-trajectory *concept* reframes the problem in gene space, ordering *genes* within each program rather than cells along pseudotime. *GeneTrajectory* (Qu *et al.* 2025) operationalizes this with optimal-transport distances between gene-specific expression distributions over the cell–cell graph, clustering genes into programs and assigning a gene-level pseudotime within each. This lets a cell contribute to multiple programs, but its one-program-per-gene assignment again conflicts with pleiotropy, motivating models that support overlap at both levels.

### 2.3 Probabilistic scRNA-seq specific models

Probabilistic factor models infer programs as latent components with explicit uncertainty and cell-level mixtures. Representative examples include consensus NMF (cNMF), which stabilizes parts-based decompositions across repeated runs (Kotliar *et al.* 2019), *scHPF*, a hierarchical Poisson factorization aligned with UMI count statistics (Levitin *et al.* 2019), and Bayesian NMF variants such as *scCoGAPS* (Stein-O’Brien *et al.* 2019). Topic models themselves have been applied to single-cell data. Grade-of-membership/LDA models visualize scRNA-seq structure and annotate topic gene sets by Gene Ontology enrichment (Dey *et al.* 2017; Carbonetto *et al.* 2023), and *scETM* learns embedded topics as interpretable gene signatures (Zhao *et al.* 2021). Indeed, Kotliar *et al.* (2019) benchmarked LDA alongside NMF and independent component analysis and found it recovers GEPs competitively with matrix factorization. Informed Dirichlet priors for single-cell LDA also exist: Min *et al.* (2023) replaced the uniform prior with a matrix prior transferred from a reference dataset’s fitted topics. PRISM-GEP differs in the *source* of the prior. It uses a per-gene prior estimated from within-dataset gene–gene co-expression, with no external database and no reference fit. This prior is a data-driven initialization that leaves LDA’s generative process unchanged, and programs are characterized directly by their co-expression coherence and by GO Biological Process coverage and enrichment strength (these measures are defined in Section 5.1).

## 3 Preliminaries

This section introduces the key mathematical tools underpinning our approach.

### 3.1 Latent Dirichlet Allocation

LDA (Blei *et al.* 2003) is a generative probabilistic model in which each document is a mixture over $K_{\text{topics}}$ latent topics, and each topic is a distribution over words. (We write $K_{\text{topics}}$ for the number of topics throughout, and $k_{\text{nn}}$ for the cell-neighborhood size introduced in Section 4.2, to keep the two distinct.) For each document *d*, topic proportions $\theta_{d}$ are drawn from a Dirichlet distribution with parameter α. Each word $w_{dn}$ is then generated by first sampling a topic $z_{dn}∼\text{Multinomial}(\theta_{d})$, followed by sampling the word from the corresponding topic–word distribution, drawn from a Dirichlet distribution with parameter β.

In the original formulation of LDA, β denotes the *multinomial topic–word distribution*. In contrast, implementations based on collapsed Gibbs sampling, including MALLET (McCallum, 2002), treat β as the *Dirichlet parameter* over topic–word distributions. Throughout this work, we adopt the latter convention, consistent with MALLET and other Gibbs-based LDA implementations.

Inference is commonly performed using *Collapsed Gibbs Sampling* (Griffiths and Steyvers, 2004), which samples topic assignments $z_{dn}$ based on current token counts and the priors α and β. This method, implemented efficiently in MALLET, is a widely adopted baseline.

### 3.2 Pointwise mutual information and variants

PMI (Church and Hanks, 1990) measures the strength of association between two words $w_{i}$ and $w_{j}$, by comparing their observed co-occurrence probability (joint) to the expected probability if they were independent: $\text{PMI}(i,j)=\text{log}\;\frac{P(i,j)}{P(i)P(j)}$. The intuition behind PMI is based on the assumption that if $w_{i}$ and $w_{j}$ are independent, their joint probability equals the product of their marginal probabilities, i.e., $P(w_{i},w_{j})=P(w_{i})P(w_{j})$. A positive PMI indicates higher-than-expected co-occurrence (positive correlation), a negative PMI indicates less frequent co-occurrence (negative correlation), and a zero PMI implies statistical independence. To remove noisy or uninformative associations, Positive PMI (PPMI) retains only non-negative values: PPMI(i,j)=max{PMI(i,j),0}.

### 3.3 Diffusion maps

Diffusion maps (Coifman and Lafon, 2006) provide a nonlinear dimensionality reduction technique that captures the intrinsic geometry of data represented as a similarity graph. Given an undirected gene–gene similarity graph *G*, the method constructs a Markov transition matrix *P* according to the following formulation: $P=D^{-1}G,$ where *D* is the diagonal degree matrix with entries $D_{ii}=\sum_{j}G_{ij}$. Then, it performs eigen-decomposition over *P* to obtain the diffusion embedding. Each gene is then embedded as a vector:


$$\Psi_{t}(w_{i})=\left(\lambda_{1}^{t}\psi_{1}(i),\lambda_{2}^{t}\psi_{2}(i),\ldots ,\lambda_{m}^{t}\psi_{m}(i)\right),$$

where $\lambda_{k}$ and $\psi_{k}$ are the *k*-th eigenvalue and eigenvector of the transition matrix, respectively; *m* is the embedding dimension, and *t* is the diffusion time controlling the decay.

## 4 Proposed method: Effective LDA initialization

We introduce PRISM-GEP, a method that improves gene-expression-program modeling by providing a data-driven initialization for LDA (implemented by the MALLET toolkit, https://mimno.github.io/Mallet/). The approach consists of two main stages: (i) construct *gene embeddings* from scRNA-seq statistics alone, and (ii) convert these embeddings into a Dirichlet parameter β over topic–gene (GEP–gene) distributions. Unlike the usual scalar (symmetric) prior, this β is a per-gene vector with one concentration per gene, so it can encode which genes tend to co-occur within a program. β initializes LDA and guides inference toward biologically coherent modules without modifying the generative model. This LDA stage performs the first step of trajectory frameworks, gene-program discovery. The resulting GEPs feed the second step, gene ordering, which we recover from the same construction and benchmark against dedicated trajectory methods.

### 4.1 Rationale

scRNA-seq captures heterogeneous, multiplexed GEPs whose recovery is central to mechanism-of-action studies, target discovery, and the interpretation of tissue-specific phenotypes. The computational challenge is to infer these programs directly from noisy count data while accommodating many-to-many relationships: cells co-activating multiple programs and genes contributing to several programs in a context-dependent manner.

A natural reduction links text corpora to scRNA-seq (see Fig. 2). The document-term matrix corresponds to the cell-gene UMI count matrix. Documents map to cells, words to genes, and topics to biological programs. In both settings, counts reflect discrete sampling events, documents/cells may mix several topics/programs, and words/genes may belong to multiple topics/programs depending on context. This alignment motivates topic modeling for scRNA-seq as a principled way to uncover latent structure from high-dimensional counts.

**Figure 2:**
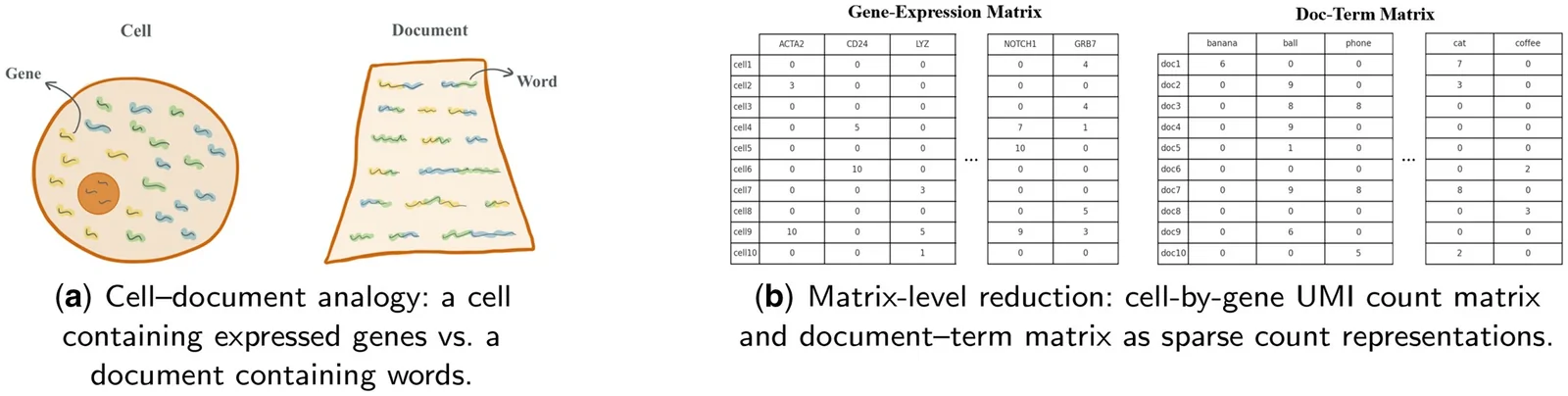
Conceptual correspondence between scRNA-seq and text corpora: (a) a cell with expressed genes versus a document with words, where each color marks a different gene program (topic) that the gene or word participates in, and (b) their count-based matrix representations, whose rows index cells or documents and whose columns index genes or words.

Within this framework, LDA is particularly appealing: its mixed-membership generative process matches the biological desiderata, namely cells that co-activate multiple programs and genes with probabilistic, overlapping participation, while remaining interpretable and lightweight. Because it models discrete counts directly, LDA also operates natively on the sparse cell-by-gene matrix, without the dense normalization or imputation that many factorizations require. Our premise is that LDA’s behavior can be improved, without altering its generative structure, by initializing it with biological signal from the dataset itself. Concretely, PRISM-GEP derives an informative topic-gene prior from the empirical gene–gene co-expression geometry of the very dataset under study, thereby providing a more biologically grounded starting point for inference. This design targets Step (i) (robust gene-set identification) while remaining fully compatible with Step (ii) (gene ordering), which we recover with a diffusion-based ordering.

### 4.2 Constructing gene embeddings

#### 4.2.1 Cell–cell graph construction

To measure functional similarity between genes, we ask which genes are switched on together across similar cells. We reduce the cells to their top 50 principal components, computed on library-size-normalized log counts, and for each cell find its $k_{\text{nn}}=15$ nearest neighbors in that space under Euclidean distance, the Scanpy and Seurat default (Wolf *et al.* 2018; Stuart *et al.* 2019). Every cell therefore defines one neighborhood, made of that cell itself plus those 15 neighbors, so there are as many neighborhoods as there are cells and they overlap heavily. Within each neighborhood we then mark a gene as present if it is detected in at least one of the neighborhood’s 16 cells, which turns every neighborhood into a set of genes. Pooling in this way is what recovers a gene that dropout has hidden in any single cell. Two genes are joined when they appear in many of the same neighborhoods, the pattern expected of genes acting in a shared biological process (Eisen *et al.* 1998). We weight each edge by PPMI (Section 3.2), which discounts how common a gene is on its own, so that ubiquitous genes do not dominate. We give both constructions in full, with the exact matrices and thresholds, in Supplementary Material S1, available as supplementary data at *Bioinformatics* online. The kNN graph only supplies the grouping, so Metacells (Baran *et al.* 2019), SEACells (Persad *et al.* 2023), and Milo-like (Dann *et al.* 2022) constructions can replace it, and we benchmark those alternatives in Supplementary Material S6.5, available as supplementary data at *Bioinformatics* online. The result is an undirected gene–gene graph *G* that links genes co-activated across neighborhoods.

#### 4.2.2 From graph to embeddings

To obtain dense representations of genes from the similarity graph *G*, we apply diffusion maps as defined in Section 3.3. This spectral embedding technique captures higher-order, indirect relationships by modeling multi-step random walks on the graph: genes that participate in similar expression programs will exhibit similar diffusion transition profiles over time, reflecting shared cellular microenvironments.

We embed each gene using the top *m* diffusion components, i.e., the leading eigenvectors of the diffusion operator scaled by their corresponding eigenvalues (empirically used m=20). The resulting vectors are gene embeddings that encode biological relatedness inferred solely from the observed scRNA-seq data.

### 4.3 Estimating Dirichlet parameter β

#### 4.3.1 GEP–gene distributions

To compute empirical GEP–gene distributions from the learned gene embeddings, we fit a GMM (Reynolds, 2009) with $K_{\text{topics}}$ components to the gene representations. The GMM yields posterior responsibilities p(z|g) of each gene *g* belonging to GEP *z*, together with mixture weights p(z).

Our goal, however, is to obtain p(g|z), the probability of a gene given a GEP, as required to estimate the Dirichlet parameter β over GEP–gene distributions. To do so, we apply Bayes’ rule:


$$p(g|z)=\frac{p(z|g)\;p(g)}{p(z)},$$

where p(z|g) is given by the GMM, p(z) is the mixture weight of component *z*, and p(g) is the empirical gene frequency computed in previous steps. Stacking these conditional probabilities yields a GEP–gene matrix


$$X\in R^{K_{\text{topics}}\times V},\;X_{g}^{\left(z\right)}=p(g|z),$$

where each row $X^{\left(z\right)}\in \Delta^{V-1}$ is a multinomial distribution over the *V* genes for GEP *z*. This matrix is grounded entirely in signals derived from the data itself, capturing both cellular microenvironment and gene-level frequency, and we treat its $K_{\text{topics}}$ row-wise distributions $X^{\left(1\right)},\ldots ,X^{\left(K_{\text{topics}}\right)}$ as empirical samples from a Dirichlet-distributed GEP–gene distribution when estimating the parameter β in the next step.

#### 4.3.2 Parameter β estimation

To estimate a Dirichlet parameter $\beta \in R^{V}$ over the vocabulary, we apply the method of moments (Minka, 2000), a classical statistical technique for parameter estimation. The core idea is to match the theoretical moments of the Dirichlet distribution to empirical moments computed from data. Let $X^{\left(1\right)},\ldots ,X^{\left(k\right)}\in \Delta^{V-1}$ be *k* observed multinomial parameter vectors over genes (in our case, $k=K_{\text{topics}}$), assumed to be drawn from a $\text{Dirichlet}(\beta_{1},\ldots ,\beta_{V})$ distribution over the (V−1)-simplex. We write $X_{i}^{\left(\ell \right)}$ for the *i*-th component of sample $X^{\left(\ell \right)}$, and $X_{i}$ for the corresponding scalar random variable. The method-of-moments estimator for $\beta_{i}$ is


$${\hat{\beta }}_{i}=E[X_{i}]\left(\frac{E[X_{i}]\left(1-E[X_{i}]\right)}{V[X_{i}]}-1\right),$$

where


$$E[X_{i}]\approx \frac{1}{k}\sum_{\ell =1}^{k}X_{i}^{\left(\ell \right)},\;V[X_{i}]\approx \frac{1}{k-1}\sum_{\ell =1}^{k}{\left(X_{i}^{\left(\ell \right)}-E[X_{i}]\right)}^{2}.$$

#### 4.3.3 Initializing LDA with $\hat{\beta }$

We use the estimated parameter $\hat{\beta }$ to initialize LDA so that each topic (GEP) is a priori biased toward genes that co-activate in the scRNA-seq. That way, genes with stronger evidence of shared regulation receive greater prior mass. This replaces the usual uniform or scalar prior and encodes the data’s own co-regulation structure at initialization.

To enable this in practice, we extended MALLET to accept a vector-valued $\hat{\beta }$ (rather than a single scalar), leaving the LDA generative model and inference (e.g., collapsed Gibbs sampling) otherwise unchanged. Thus, PRISM-GEP steers the sampler toward biologically coherent gene modules while allowing the posterior to adapt to the observed counts.

## 5 Experiments

Our goal is to evaluate whether PRISM-GEP matches or exceeds widely used specialized scRNA-seq methods for gene-program discovery and whether the data-driven Dirichlet parameter $\hat{\beta }$ improves on standard (uniform-prior) LDA. We assessed performance on a range of biologically diverse datasets, comparing PRISM-GEP primarily against specialized single-cell factorizations and, as a dedicated control that isolates the effect of the informed prior, against its uniform-prior parent (MALLET). Evaluation is based on four complementary metrics that probe the first step of the trajectory-based analysis pipeline: gene-program identification. In addition, we include a quantitatively benchmarked experiment addressing the second step, namely within-program gene ordering.

### 5.1 Experimental design

#### 5.1.1 Baselines

We compare primarily against established single-cell factorizations: cNMF (Kotliar *et al.* 2019) and scHPF (Levitin *et al.* 2019) (strong specialized methods), NMF (a parts-based floor), and ProdLDA (Srivastava and Sutton, 2017) (neural LDA). To isolate our contribution, we additionally compare against vanilla MALLET LDA, which replaces our vector-valued β with a uniform Dirichlet prior and is otherwise identical. All run on the same highly variable gene (HVG)-filtered matrix at identical $K_{\text{topics}}$ over ten seeds. The count-native methods take raw counts, and NMF takes the conventional log1p counts-per-10k (CP10K) input. Seeding details are provided in the Supplementary Material, available as supplementary data at *Bioinformatics* online.

#### 5.1.2 Metrics

Each GEP is summarized by its top-N=20 genes and scored for biological quality along four complementary axes, each chosen to probe a distinct failure mode. *Coherence* (mean pairwise Spearman of the top-*N* genes across cells) asks whether the genes a program groups are actually co-expressed. *Coverage* [the fraction of a GEP’s annotated genes in significantly enriched Gene Ontology Biological Process (GO-BP) pathways] asks whether the program corresponds to known biology. *Strength* (the mean $-{\;\text{log}\;}_{10}q$, FDR-adjusted, over a GEP’s significant pathways) asks how sharp that enrichment is. Finally, an *LLM-based plausibility* score (Hu *et al.* 2025) asks GPT-4 whether a program’s top genes plausibly co-participate in a shared Biological Process and returns a confidence value, a functional interpretability proxy that complements the count-based statistics. Together the four probe co-expression, known-pathway recovery, enrichment sharpness, and expert-judged plausibility. The second step, within-program gene ordering, is scored separately by the absolute Spearman rank correlation |ρ| between the recovered gene order and a canonical marker order. Full metric definitions are in Supplementary Material S5, available as supplementary data at *Bioinformatics* online.

#### 5.1.3 Datasets

Our GO-BP benchmark spans a diverse panel of published scRNA-seq datasets ($∼10^{3}$–$∼10^{5}$ cells, human and mouse, developmental and steady-state regimes). Figure 3 summarizes the full benchmark, and the complete per-dataset tables are in the Supplementary Material (Table S7, available as supplementary data at *Bioinformatics* online). The datasets we describe in detail are BreastCancer (a curated clinical panel, 2,748 cells × 297 genes), PBMC3k, Zeisel cortex, Pancreas (endocrine differentiation), Bonemarrow (ten hematopoietic types, a stress test for small $K_{\text{topics}}$), Hemogenic Endothelium (an endothelial-to-hematopoietic lineage), and three slices of the mouse gastrulation atlas (full, E7.5, and erythroid, with “full” an 8,000-cell subsample of Pijuan-Sala *et al*. 2019). All are preprocessed identically (Supplementary Material S3.1, available as supplementary data at *Bioinformatics* online).

**Figure 3:**
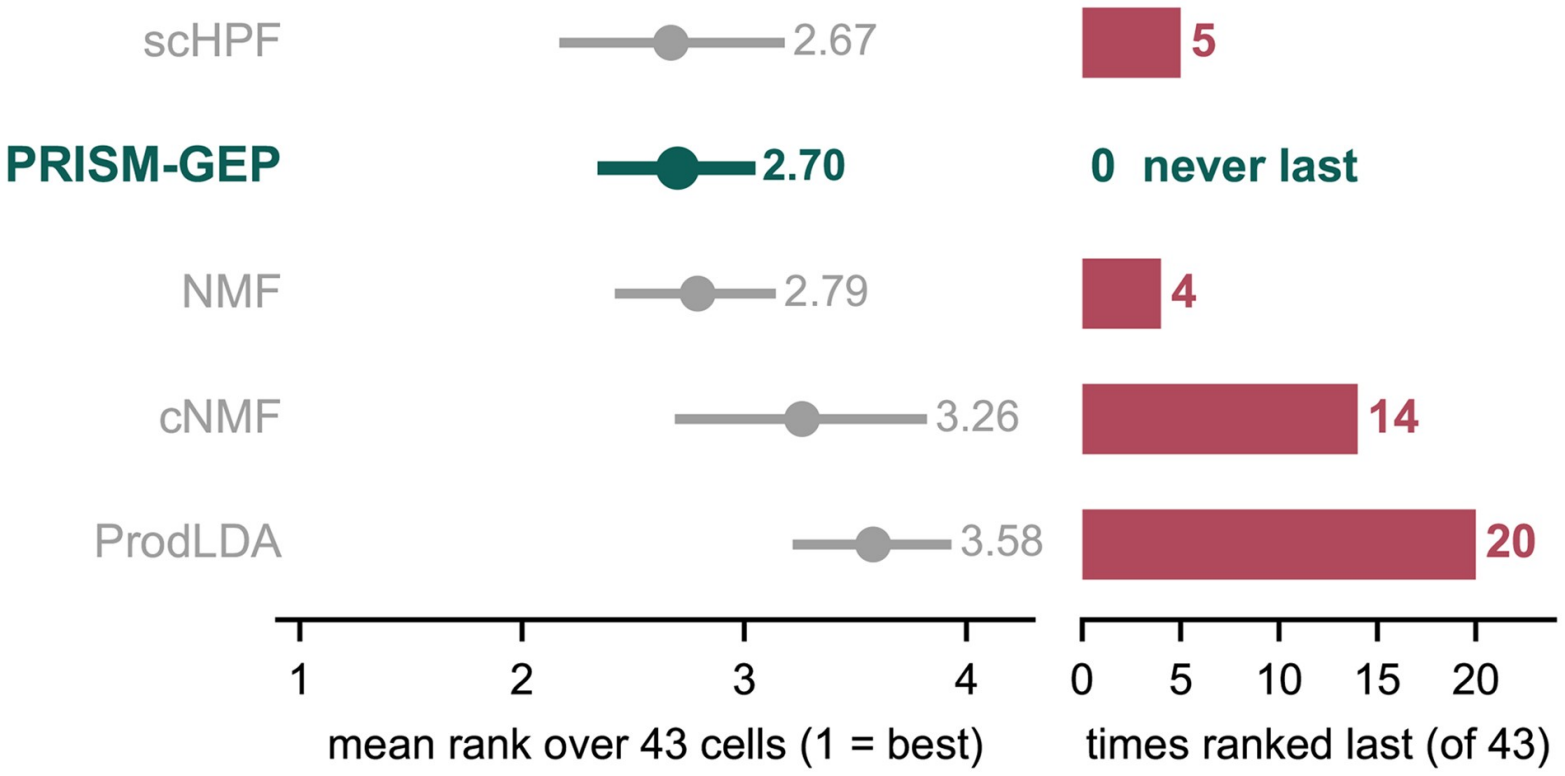
PRISM-GEP is robust across the benchmark. Mean rank (1 = best, 95% bootstrap CI over datasets) and number of last places across the 43 rankable dataset × metric GO-BP evaluations. PRISM-GEP never ranks last and its mean rank is level with the best. Full rank distribution, aggregate statistics, and per-dataset values are in Fig. S5 and Tables S6 and S7, available as supplementary data at *Bioinformatics* online.

#### 5.1.4 Hyperparameters

We fix $K_{\text{topics}}=5$ topics and top-N=20 genes per program as a single untuned configuration applied uniformly across datasets (a conservative under-specification when cell types are many). We also disable MALLET’s periodic hyperparameter re-estimation (optimization interval 0), so the informed β prior is used exactly as estimated rather than re-optimized during inference. The re-estimating variant is reported in the Supplementary Material, available as supplementary data at *Bioinformatics* online. Robustness to the neighborhood size $k_{\text{nn}}$, the topic count $K_{\text{topics}}$, the cell-level readout (the per-cell GEP-attribution vectors), and the cell–cell graph is stress-tested in Supplementary Material S9.1, S6.4 and S6.5, available as supplementary data at *Bioinformatics* online. Program quality is stable to the neighborhood size and to the cell-level readout. For the cell–cell graph no single construction is best on every dataset, so we keep the standard kNN as a simple default and a practitioner may substitute whichever graph suits their data.

### 5.2 Results

We report both stages of the method in turn, gene-program discovery (Step (i)) and within-program gene ordering (Step (ii)), together with the robustness and interpretability analyses.

#### 5.2.1 Step (i): Gene-program discovery results

We report Step (i) results across all datasets: GO-BP program quality against the specialized methods (Fig. 3, with per-dataset values in Table S7, available as supplementary data at *Bioinformatics* online) and the LLM-based plausibility score (Fig. 4).

**Figure 4:**
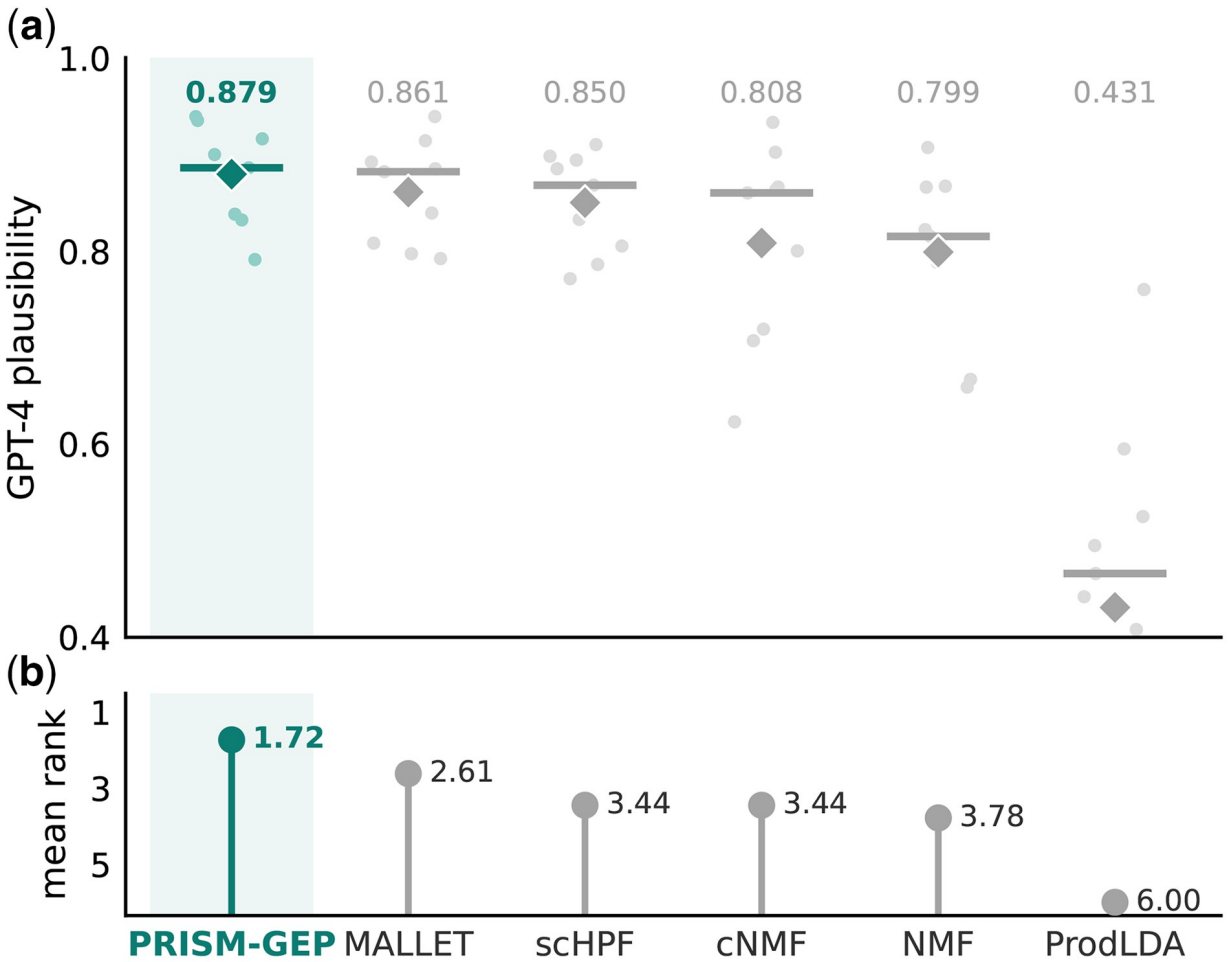
GPT-4 plausibility across nine LLM-scored datasets. (a) Per-method plausibility, one point per dataset, with the method mean (⋄) and median (tick). The axis starts at 0.4, so two low ProdLDA points fall off-scale. (b) Mean rank (1= best). Values and per-dataset scores are in the text and in Table S8, available as supplementary data at *Bioinformatics* online.

No method is uniformly best. Each specialized factorization wins some (dataset, metric) entries and ranks last on others. PRISM-GEP is never last on any of the 43 rankable entries and nearly ties the best method on mean rank, avoiding the single-metric last-place finishes that lower the specialists’ mean rank (Supplementary Material S6.1, available as supplementary data at *Bioinformatics* online). Program quality is thus competitive with methods built specifically for single-cell data, and its consistency across the benchmark is what sets it apart. The per-dataset numbers, against both the specialized methods and the uniform-prior MALLET parent, are in Table S7, available as supplementary data at *Bioinformatics* online.

An LLM-based plausibility score agrees from a functional-interpretability angle. Asked whether a program’s top genes plausibly share a biological process, GPT-4 rates PRISM-GEP highest overall across nine scored datasets, on the mean, the median, and the mean rank alike (Fig. 4, per-dataset scores in Table S8, available as supplementary data at *Bioinformatics* online).

#### 5.2.2 Step (ii): within–topic gene ordering

Beyond discovering gene programs (Step (i)), PRISM-GEP also supports Step (ii): an intrinsic ordering of genes within each program. We adapt the diffusion-based gene ordering of GeneTrajectory (Qu *et al.* 2025) to PRISM-GEP’s gene–gene similarity, which is the same co-expression geometry that defines the β prior (Section 4.2, Supplementary Material S2, available as supplementary data at *Bioinformatics* online). From each GEP, we take the first non-trivial diffusion component as a one-dimensional gene ordering, a “trajectory” along a continuum of co-activation. We validate this ordering in three ways. Against published marker orders it recovers known developmental sequences (Fig. 5) and matches dedicated methods on the benchmark below. Against a cell axis the method never sees, the recovered orders form clean diagonal cascades when cells are sorted by an independent Slingshot pseudotime, shown alongside GeneTrajectory and a root-supervised DPT baseline (Fig. S6, available as supplementary data at *Bioinformatics* online). On synthetic simulators with a known activation order, it reconstructs the cascade from expression alone at |ρ|∈[0.81,0.98], the branching simulators scored within lineages (Fig. S11, available as supplementary data at *Bioinformatics* online). The recovered trajectory for five representative datasets is shown in Fig. S9, available as supplementary data at *Bioinformatics* online. Because genes keep probabilistic membership in several programs, a gene may appear in more than one ordering rather than being forced into a single trajectory.

**Figure 5:**
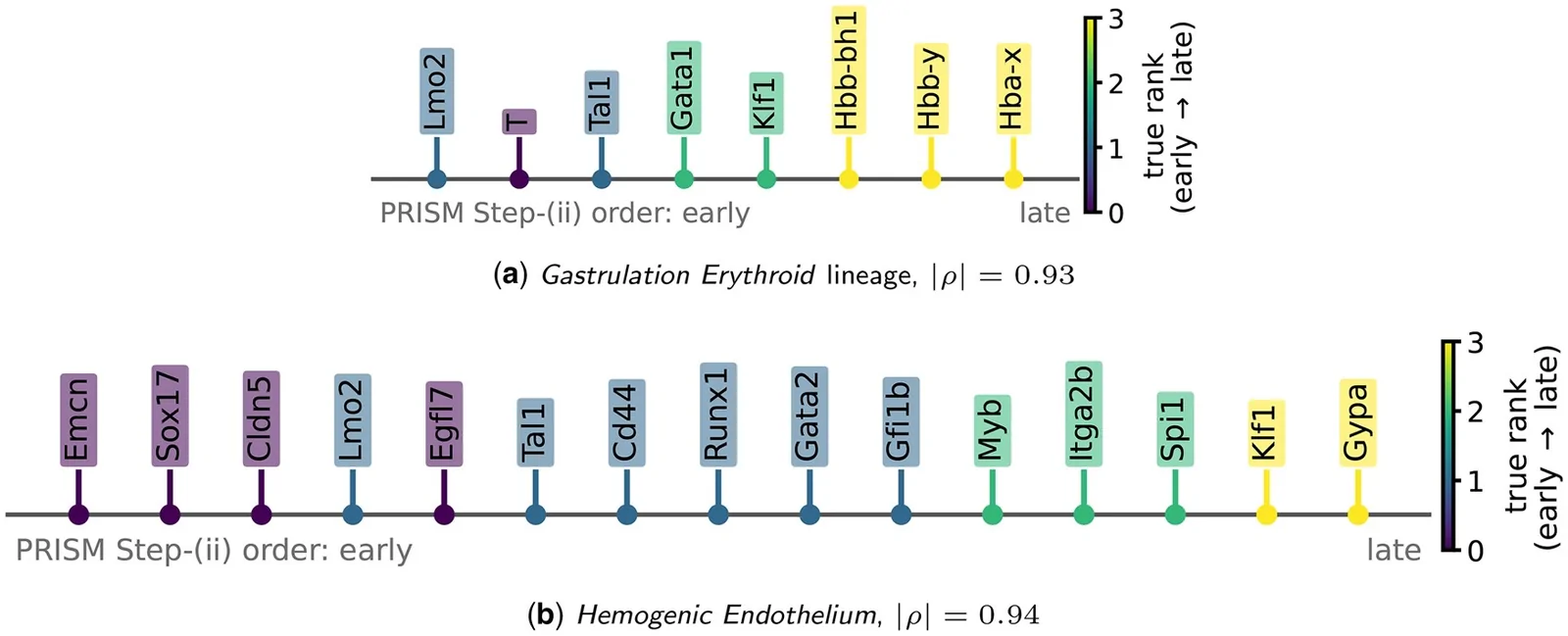
PRISM-GEP recovers known developmental gene trajectories. Markers are ordered by PRISM-GEP Step (ii) and colored by canonical rank (dark early, yellow late); a smooth gradient indicates agreement. (a) Mouse primitive erythropoiesis. (b) Endothelial-to-haematopoietic transition. Each panel shows one fit, so its |ρ| may differ from the ten-fit mean in Table 1a (0.930 and 0.928, respectively).

#### 5.2.3 Gene-trajectory benchmark

We benchmark the gene ordering against established methods on the datasets that carry a canonical marker order (Fig. 6), ordering each program’s genes by a Hellinger diffusion coordinate (Supplementary Material S2, available as supplementary data at *Bioinformatics* online). The result is on par with the dedicated GeneTrajectory method (Qu *et al.* 2025). PRISM-GEP holds the best mean and the best median and is the most consistent across datasets, GeneTrajectory and the root-supervised DPT-weighted-mean baseline each lead on individual datasets, and every marker-bootstrap confidence interval overlaps on every dataset (Table S11, available as supplementary data at *Bioinformatics* online). We therefore read the methods as on par rather than ranked. To keep the comparison fair, GeneTrajectory is a multi-trajectory method scored three ways against the single-lineage marker order (best-matching trajectory, native extract, and a leading-eigenvector re-scoring), and DPT is scored only where a root cell is defined.

**Figure 6:**
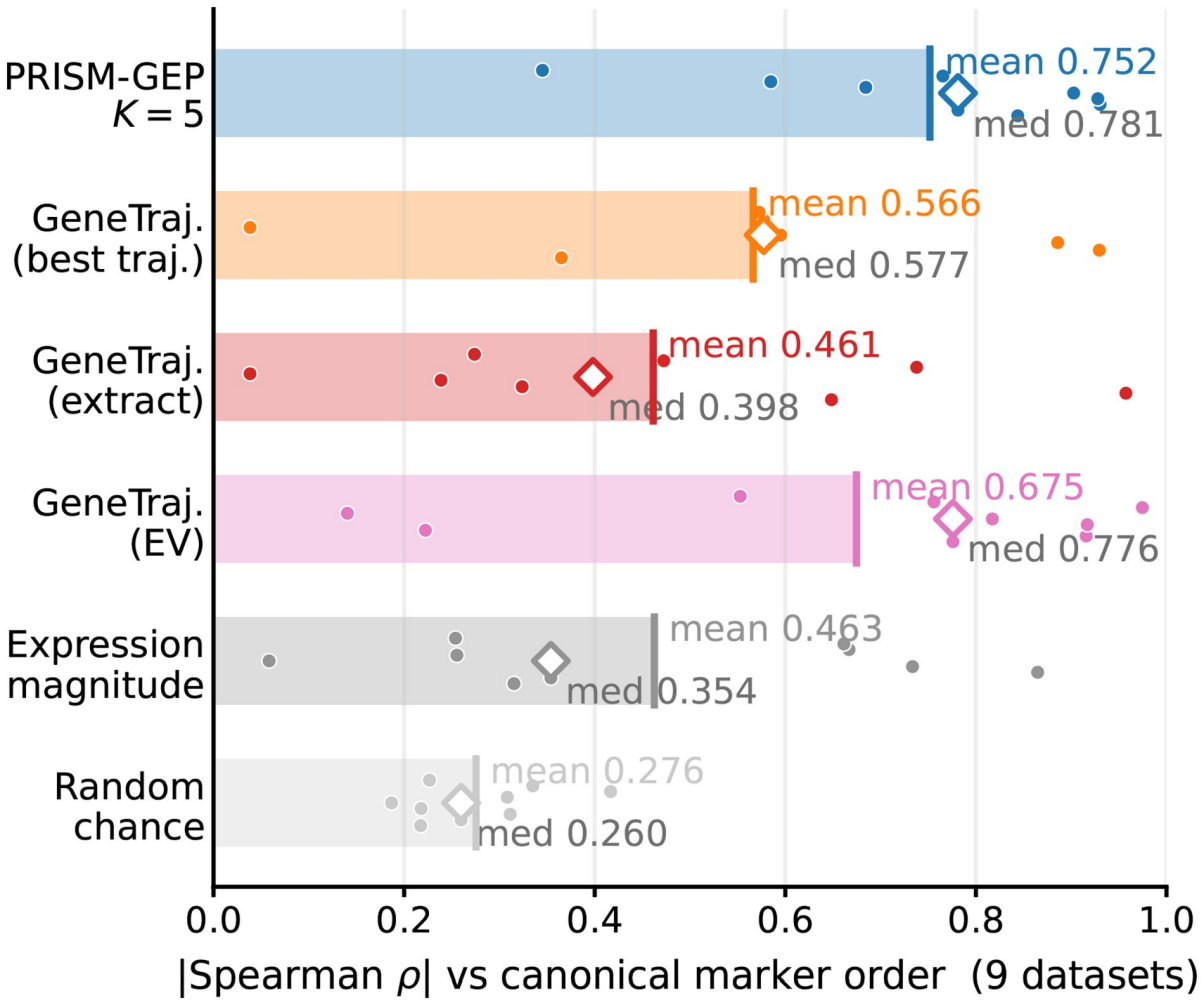
Gene-trajectory recovery across nine datasets with canonical marker orders. Bars, diamonds, and dots show the mean, median, and per-dataset |Spearman ρ|, respectively. Supplementary Tables S11 and S12, available as supplementary data at *Bioinformatics* online, report bootstrap CIs and the configuration sweep.

They are not on par everywhere. Under severe class imbalance, the methods separate: on Hemogenic Endothelium, where 86% of cells are endothelial, PRISM-GEP holds at a bootstrap mean of 0.912 while root-supervised DPT falls to 0.765 (Table S11, available as supplementary data at *Bioinformatics* online), and on the ten-seed means of Table 1a PRISM-GEP is above the chance level on all 9 datasets, that level being the mean of 10,000 random orderings per dataset rather than a single draw. It also exceeds the log1p ordering on all nine, a baseline that is ill-conditioned on six of them (Supplementary Material S7.3, available as supplementary data at *Bioinformatics* online). On the five-marker Dentate Gyrus and the ill-posed Gastrulation E7.5, the margin over chance is under one chance-level standard deviation, so neither is evidence of recovery. The methods also differ in what they cost. PRISM-GEP requires a single eigendecomposition of the gene–gene diffusion operator, which the GEP decomposition has already produced, whereas GeneTrajectory solves an optimal-transport distance between *every* gene pair. Equal accuracy is therefore obtained as a by-product rather than from a dedicated computation.

**Table 1:** Gene ordering across nine datasets, measured by |ρ| between recovered and canonical marker orders.

Method	Panc.	Gastr.	Eryth.	Hemog.	Bone.	Paul15	DG	Endo.	E75	Mean	Median	Mean rank
**(a) Full marker set**												
Markers	n=15	n=11	n=8	n=15	n=20	n=8	n=5	n=7	n=14			
PRISM-GEP	**0.844 (0.057)**	**0.781 (0.039)**	**0.930 (0.012)**	**0.928 (0.013)**	**0.903 (0.004)**	**0.684 (0.043)**	**0.585 (0.043)**	**0.765 (0.030)**	**0.345 (0.238)**	**0.752**	**0.781**	**1.00**
log1p	0.765	0.160	0.618	0.674	0.089	0.192	0.359	0.661	0.126	0.405	0.359	2.56
Random	0.217 (0.158)	0.260 (0.186)	0.311 (0.218)	0.218 (0.158)	0.187 (0.137)	0.308 (0.219)	0.417 (0.276)	0.335 (0.234)	0.227 (0.164)	0.276	0.260	2.44
(**b) scGPT-embeddable marker subset**												
Markers	n=13	n=8	n=4	n=15	n=20	n=4	n=5	n=7	n=13			
PRISM-GEP	**0.875 (0.050)**	**0.751 (0.047)**	0.894 (0.000)	0.928 (0.013)	0.903 (0.004)	**0.949 (0.000)**	0.585 (0.043)	0.765 (0.030)	0.326 (0.251)	**0.775**	**0.875**	1.78
scGPT static	0.831	0.342	0.894	0.064	0.860	0.316	**0.975**	0.643	0.414	0.593	0.643	2.44
scGPT contextual	0.710 (0.000)	0.732 (0.000)	0.894 (0.000)	**0.954 (0.000)**	0.903 (0.003)	0.316 (0.000)	0.800 (0.119)	**0.850 (0.000)**	**0.754 (0.023)**	0.768	0.800	**1.56**

(a) PRISM-GEP and baselines are evaluated on the full marker set. (b) PRISM-GEP and scGPT are evaluated on the same subset of markers within scGPT’s vocabulary. Markers outside it are excluded. Parentheses show SD across ten seeds, except for Random, which reports the mean and SD of 10,000 random orderings. scGPT static is deterministic. **Bold** marks the best value in each column.

#### 5.2.4 Comparison against pretrained gene embeddings

Because Step (ii) relies on gene–gene similarity, we compare our dataset-intrinsic PPMI with static and contextual scGPT embeddings (Cui *et al.* 2024). We score |Spearman ρ| against canonical marker orders on the strict scGPT-embeddable intersection (Table 1b). PRISM-GEP and contextual scGPT are statistically indistinguishable (p=0.95, paired *t*-test), each leading on some datasets. Thus, a similarity derived from the dataset matches a transformer pretrained on tens of millions of cells without external data or pretraining. Their strengths differ, however. scGPT leads on Dentate Gyrus and *Gastrulation E7.5*, where its corpus better covers the biology. Its human-only static vocabulary misses mouse markers such as embryonic globins, contextualization repairs this weakness and leads on Hemogenic Endothelium. Pretrained embeddings are therefore preferable when corpus coverage is strong, whereas dataset-intrinsic similarity is safer when coverage is poor or unknown. Results on individual datasets remain uncertain because some intersections contain few markers, and PRISM-GEP varies widely across seeds on *Gastrulation E7.5*. Supplementary Material S7.3, available as supplementary data at *Bioinformatics* online, provides per-dataset results and the corrected log1p entries.

#### 5.2.5 Cell ordering, a by-product

The same GEP-attribution vectors also order *cells*, without labels, using the leading Jensen–Shannon diffusion-map coordinate (Coifman and Lafon, 2006; Haghverdi *et al.* 2015, 2016). Scored on the same nine datasets against the published lineage rank, a different ground truth from the gene orderings above and not comparable with them, it is competitive (Table 2): it has the best median and the best mean rank of the four methods, and, excluding the two (^a^) columns that cannot separate methods, is best on three of the remaining seven datasets and worst on one, with DPT ahead on the mean (0.727 against 0.722) and Slingshot clearly better on Dentate Gyrus (0.840 against 0.571). One of the four is a trivial first-principal-component floor that stays close on several datasets, so these correlations should be read against it. We treat this as a proof of concept rather than a benchmarked contribution of this paper, since PRISM-GEP is a gene-trajectory method and we have not benchmarked cell ordering to the same depth. The discussion and the metric ablation are in Supplementary Material S8 (Table S13), available as supplementary data at *Bioinformatics* online.

**Table 2:** Cell ordering across nine datasets: |ρ| between the recovered cell order and the published lineage rank.

Method	Panc.	Gastr.	Eryth.	Hemog.^a^	Bone.	Paul15	DG	Endo.^a^	E75	Mean	Median	Mean rank
PRISM-GEP	**0.943**	0.527	0.804	**0.267**	**0.896**	**0.873**	0.571	0.746	0.867	0.722	**0.804**	**2.11**
Slingshot	0.940	**0.644**	0.793	0.127	0.891	0.519	**0.840**	0.737	0.898	0.710	0.793	2.67
DPT/PAGA-DPT	0.933	0.607	**0.849**	0.206	0.837	0.775	0.715	0.707	**0.910**	**0.727**	0.775	2.44
PCA-1	0.912	0.201	0.815	0.217	0.893	0.699	0.490	0.746	0.879	0.650	0.746	2.78

PRISM-GEP values are means over ten seeds.

a Marks a published order with only two ranks, too coarse to separate methods. PCA-1 is a trivial first-principal-component floor.

**Bold** marks the best value in each column.

#### 5.2.6 PRISM-GEP versus MALLET

Vanilla LDA (MALLET) is already competitive, matching scHPF on most tasks, whereas the non-count models ProdLDA and NMF underperform. PRISM-GEP’s informed β prior ties MALLET on the count-based GO-BP metrics: 39 of 43 entries are statistically indistinguishable, and the other four split evenly between the methods (Table S7; Supplementary Material S6.6, available as supplementary data at *Bioinformatics* online). PRISM-GEP nevertheless has the best mean, median, and rank on the LLM plausibility score (0.879/0.886/1.72, versus MALLET’s 0.861/0.882/2.61; Fig. 4), although PRISM-GEP, MALLET, and scHPF remain within ∼0.03. Most importantly, PRISM-GEP also provides an intrinsic within-program gene ordering and grounds the decomposition in the dataset’s own co-expression structure. Thus, at comparable GO-BP quality, it adds interpretability that vanilla LDA does not provide.

## 6 Conclusion

We introduced PRISM-GEP, a data-driven initialization of LDA tailored to single-cell RNA-seq. By deriving a topic–gene prior directly from gene–gene similarity statistics, PRISM-GEP leaves the LDA generative process unchanged yet reshapes its inductive bias.

This study makes three main contributions. First, PRISM-GEP provides a *self-contained framework* for discovering GEPs: its prior is learned from the dataset’s own co-expression structure and therefore requires neither a reference atlas nor a curated pathway database. Second, it delivers *robust and competitive program quality*. Across all 43 rankable dataset–metric entries in the GO-BP benchmark, PRISM-GEP never ranks last and achieves a mean rank level with the best-performing method, whereas every specialized factorization ranks last at least once (Fig. 3, Table S7, available as supplementary data at *Bioinformatics* online). It also ranks first by mean, median, and mean rank in the LLM-based evaluation of biological plausibility across nine datasets (Fig. 4). Third, PRISM-GEP provides an *intrinsic ordering of genes within each program* at no additional cost. This ordering recovers published developmental sequences (Fig. 5) and is comparable to GeneTrajectory (Qu *et al.* 2025) across datasets (Fig. 6). Although GeneTrajectory leads under individual scoring variants on individual datasets, PRISM-GEP holds the better mean and median across the nine, and every bootstrap interval overlaps, so we present the two as on par. Unlike vanilla LDA, PRISM-GEP thus recovers both overlapping GEPs and an interpretable ordering of the genes within them.

Empirically, LDA (MALLET) is already a strong baseline for scRNA-seq, often matching specialized single-cell factorization methods such as scHPF, and PRISM-GEP’s performance is also competitive with cNMF. Modest data-derived priors thus narrow much of the gap between a generic topic model and bespoke single-cell workflows.

More broadly, our results reinforce the analogy between text corpora and single-cell transcriptomes, with PRISM-GEP one step toward an interpretable probabilistic toolkit for dissecting cellular heterogeneity from the dataset’s own signal.

## Supplementary Material

btag728_Supplementary_Data

## Data Availability

The data underlying this article were derived from sources in the public domain. The datasets and their accessions are listed in Supplementary Material Section S3. The code, the result tables underlying all tables and figures, and a preprocessed test dataset are available at https://github.com/shaham-lab/PRISM-GEP and archived at https://doi.org/10.5281/zenodo.23259538.
