# Supplementary material for: PRISM-GEP: Viewing single-cell expression through the lens of topic modeling
Source: Bioinformatics. 2026 Sep 29;42(10):btag728. doi: 10.1093/bioinformatics/btag728 (PMC13661266; doi:10.1093/bioinformatics/btag728)
Supplement: Supplementary file 1 [file btag728_supplementary_data.pdf]

Bioinformatics, 2026, pp. 1–23
DOI HERE
Advance Access Publication Date: Day Month Year
Supplementary Material

Supplementary Material PRISM-GEP: Viewing Single-Cell Expression through the Lens of Topic Modeling

Yanir Buznah,[1] Tal Ishon[1] and Uri Shaham[1,*]

[1]Bar-Ilan University, Ramat Gan, Israel

[*]Corresponding author.

Abstract

This document provides the Supplementary Material for the manuscript "PRISM-GEP: Viewing Single-Cell Expression through the Lens of Topic Modeling." Figures, tables, equations and sections are numbered with an "S" prefix. References to the main text (e.g. Table 1) are resolved against the main article.

Keywords single-cell RNA-seq, gene-expression programs, topic modeling, supplementary material

S1 Method

S1.1 Cell neighborhoods as context windows.

PRISM-GEP builds two graphs in sequence: a graph over cells, defined in this subsection and summarized by the incidence matrix H below, and the gene–gene graph whose edges are counted off it (§S1.2) and converted to PPMI weights (§S1.3). Let $\mathcal{M} \in \mathbb{R}_{\geq 0}^{C\times V}$ be the preprocessed cell-by-gene matrix, where C is the number of cells, V is the number of genes and all entries are non-negative. Using individual cells (rows of $\mathcal{M}$) as context windows could be misleading by producing extremely sparse and unstable co-activation estimates, because most genes are unobserved in any given cell due to zero inflation ("dropout"). We therefore construct overlapping cell neighborhoods that approximate local cellular microenvironments and yield more reliable co-occurrence statistics. Specifically, we embed cells into a low-dimensional space using PCA, build a kNN graph over cells, and define each cell together with its k_{nn} nearest neighbors as a *context window*. We use the standard `scanpy`/Seurat default k_{nn}=15. Program quality barely moves across $k_{\text{nn}} \in \{5, \ldots, 50\}$, as Sec. S9.1 shows. Concretely, we normalize every cell to 10,000 counts, apply $\log(1+x)$, and reduce the result to its top 50 principal components. In that space we retrieve, for each cell c, the $k_{\text{nn}} + 1$ cells closest to c under Euclidean distance. That set contains c itself at distance zero, so the neighborhood of c is c together with its k_{nn} nearest neighbors, 16 cells in total at the default. Every cell defines exactly one neighborhood, so the number of neighborhoods equals the number of cells, $N = C$, and the neighborhoods overlap heavily. We keep the relation as retrieved and do not symmetrize it, so a cell c' may belong to the neighborhood of c without c belonging to the neighborhood of c'. Every row of the incidence matrix H below therefore has exactly $k_{\text{nn}} + 1$ nonzero entries. The pseudocode at the end of Sec. S1.2 gives the whole construction step by step. This kNN-over-cells construction, standard in scRNA-seq workflows (e.g. Scanpy/Seurat[Wolf et al., 2018, Stuart et al., 2019]), preserves local manifold structure, mitigates sparsity by pooling information within neighborhoods, and scales to large datasets. We encode the resulting neighborhoods in a binary incidence matrix $H \in \{0,1\}^{N\times C}$ where N is the number of neighborhoods, and $H_{n,c} = 1$ if cell c belongs to neighborhood n. Aggregating expression via H denoises sporadic dropouts while retaining the geometry of cellular states [van Dijk et al., 2018]. Because the neighborhood enters the pipeline only through H, any construction that assigns cells to groups can replace the kNN graph. We benchmark real Metacells, SEACells and a Milo-like construction in Supplementary Material S6.5.

S1.2 Neighborhood-level co-occurrence.

To obtain neighborhood-level co-occurrence, we first binarize the expression matrix $\mathcal{M}$ to obtain $\mathcal{M}_{\text{bin}} \in \{0,1\}^{C\times V}$, where $\mathcal{M}_{\text{bin}}[c, i] = 1$ if gene i is detected in cell c and 0 otherwise. A gene counts as detected in a cell when its raw count exceeds a cutoff θ_{expr}, and we use $\theta_{\text{expr}} = 2$ in all reported runs. The binarization is applied to the raw counts, not to the normalized values used for the PCA embedding. Using the neighborhood-cell incidence matrix H, we then compute

$$Z = H\mathcal{M}_{\text{bin}} \in \mathbb{N}^{N\times V},$$

where $Z_{n,i}$ counts how many cells in neighborhood n express gene i. These counts are converted to a binary neighborhood-gene matrix

$$B = \mathbb{I}[Z \geq \tau_{\text{nhood}}] \in \{0,1\}^{N\times V},$$

© The Author(s) 2026. Copyright and licence statements to be updated by the publisher during production.

where τ_{nhood} is a minimum-support threshold. We use $\tau_{\text{nhood}} = 1$ in all reported runs, so a gene is present in a neighborhood as soon as it is detected in at least one of that neighborhood's $k_{\text{nn}} + 1$ cells. This pooling is what recovers a gene that dropout has hidden in the index cell itself. From B, we derive neighborhood-level gene–gene co-activation statistics. We first form the co-occurrence matrix

$$A \;=\; B^{\top} B \in \mathbb{R}_{\geq 0}^{V \times V},$$

so that A_{ij} is the number of neighborhoods in which genes i and j are jointly present. We set $\operatorname{diag}(A) = 0$ to ignore self-co-occurrence. In parallel, we compute gene-wise marginal counts

$$g_i \;=\; \sum_{n=1}^{N} B_{ni},$$

which give the number of neighborhoods in which gene i is active. This construction emphasizes genes that repeatedly co-activate within shared neighborhoods, rather than patterns driven solely by global expression abundance.

Algorithm S1 below states the construction end to end, from the raw count matrix to the neighborhood co-occurrence matrix A, together with the default values used in every reported run.

Algorithm S1 Cell-neighborhood construction and gene–gene co-occurrence. Defaults in parentheses are the values used in every reported run.

Require: raw count matrix $\mathbf{M} \in \mathbb{Z}_{\geq 0}^{C \times V}$ (C cells, V genes)
Require: k_{nn} neighbors per cell (15), n_{pca} components (50)
Require: θ per-cell count cutoff (2), τ_{nhood} min. cells per gene (1)
Ensure: $\mathbf{A} \in \mathbb{Z}_{\geq 0}^{V \times V}$, co-occurrence counts over neighborhoods

Embed the cells.
$\mathbf{L} \leftarrow \log\big(1 + \mathbf{M}$ rescaled to 10^4 counts/cell$\big)$
$\mathbf{Y} \leftarrow$ top n_{pca} principal components of $\mathbf{L}$ ▷ $C \times n_{\text{pca}}$

One neighborhood per cell.
for each cell $c = 1, \ldots, C$ **do**
  $\mathcal{N}(c) \leftarrow$ the $k_{\text{nn}}{+}1$ cells nearest to c in $\mathbf{Y}$ (Euclidean) ▷ contains c itself, so $\mathcal{N}(c)$ is c plus its k_{nn} neighbors
end for
There are C neighborhoods and they overlap. The relation is **not** *symmetrized: $c' \in \mathcal{N}(c)$ does not imply $c \in \mathcal{N}(c')$.*

Incidence matrix.
$\mathbf{H}[n, c] \leftarrow 1$ if $c \in \mathcal{N}(n)$, else 0 ▷ $C \times C$, every row sums to $k_{\text{nn}}{+}1$

Genes present in a neighborhood.
$\mathbf{M}_{\text{bin}}[c, i] \leftarrow 1$ if $\mathbf{M}[c, i] > \theta$, else 0
$\mathbf{Z} \leftarrow \mathbf{H}\,\mathbf{M}_{\text{bin}}$ ▷ $\mathbf{Z}[n, i] =$ cells of $\mathcal{N}(n)$ expressing gene i
$\mathbf{B} \leftarrow \mathbb{I}\big[\mathbf{Z} \geq \tau_{\text{nhood}}\big]$

Gene–gene co-occurrence.
$\mathbf{A} \leftarrow \mathbf{B}^{\top}\mathbf{B}$, then set $\operatorname{diag}(\mathbf{A}) \leftarrow 0$ ▷ $\mathbf{A}[i, j] =$ neighborhoods containing both genes
return $\mathbf{A}$

Section S1.3 then converts A into the PPMI-weighted gene–gene graph G.

S1.3 PPMI and sparsification.

Defining $P(i) = g_i/M$ and $P(i,j) = A_{ij}/M$, where $M = \sum_i g_i$ is the total co-occurrence mass, and using small additive smoothing $\epsilon > 0$,

$$\mathrm{PMI}(i,j) \;=\; \log \frac{P(i,j) + \epsilon}{P(i)P(j) + \epsilon} \;=\; \log \frac{A_{ij}\, M + \epsilon'}{g_i\, g_j + \epsilon'},$$

$$\mathrm{PPMI}(i,j) \;=\; \max\{\mathrm{PMI}(i,j), 0\}.$$

PPMI retains only positive associations, while setting non-informative or negatively associated pairs to zero. The resulting undirected similarity graph is G.

S2 Details for Step (ii)

For Step (ii), we adapt the diffusion-based gene-ordering procedure of GeneTrajectory [Qu et al., 2025] to the gene–topic space learned by PRISM-GEP. The goal is to obtain a one-dimensional "trajectory" for each gene program, along which genes are ordered by their diffusion geometry, rather than by arbitrary scores.

Gene representation.

Qu *et al.* first embed genes into a low-dimensional functional space and then operate entirely in that space. Analogously, we represent each gene by its distribution over PRISM-GEP topics (optionally reweighted by topic priors), so that genes with similar topic mixtures are close in this representation.

Gene–gene similarity kernel.

Following GeneTrajectory, we then (i) compute pairwise distances between genes in this space, and (ii) convert distances into a smooth similarity kernel. Concretely, we use the Hellinger distance between gene-level topic distributions and map it through a Gaussian kernel with bandwidth set to the median distance. The Hellinger distance is a standard distance between two probability distributions. It equals the ordinary Euclidean distance between their element-wise square roots. We use it, rather than a plain Euclidean distance, because each gene's topic vector is a set of proportions that sums to one and therefore lies on the probability simplex, where the Hellinger distance is better behaved. The cell-side ordering (main-text Table 2, with the metric ablation in Table S13) uses the Jensen–Shannon divergence instead, a symmetric and bounded measure of how different two probability distributions are, chosen for the same simplex-appropriate reason. This yields a positive, symmetric gene–gene affinity matrix that plays the same role as the diffusion kernel in Qu *et al.*.

Diffusion maps and 1D coordinate.

For each gene program (e.g., the set of top genes for a given PRISM-GEP topic), we restrict the kernel to that gene set and apply diffusion maps [Coifman and Lafon, 2006, Haghverdi et al., 2016] as in GeneTrajectory. Following Qu *et al.*, we use the first non-trivial diffusion component (the second eigenvector, EV2) as a one-dimensional coordinate on the genes, which defines their ordering along the trajectory. All diffusion and ordering steps are identical to those in the GeneTrajectory paper, differing only in the underlying gene representation (PRISM-GEP topic distributions instead of the gene embeddings obtained in their framework).

Trajectory and ordering.

Genes are then ordered by this one-dimensional diffusion coordinate, yielding a trajectory for each program. Genes that are often co-activated in similar topic mixtures lie near each other along the trajectory, whereas genes associated with different microenvironments or cell states appear farther apart. In the main text, we visualize these diffusion-derived trajectories and show that they align with known tumor–microenvironment and neuronal axes. Apart from using PRISM-GEP topic distributions as the gene representation, all steps (kernel construction, diffusion maps, use of the first non-trivial component for ordering) follow Qu *et al.*.

S3 Datasets

We evaluate on a panel of scRNA-seq datasets spanning tumor, blood, brain, pancreas, and several developmental lineages (Table S5). *BreastCancer* is a laboratory-generated matrix from a patient's breast tumor (used as released), *PBMC3k* contains peripheral blood mononuclear cells from a healthy donor, and *Zeisel_brain* profiles mouse cortical cells. The panel also includes *Pancreas* (Bastidas-Ponce et al. 2019, endocrine differentiation), *Bonemarrow* (ten hematopoietic cell types), *Hemogenic Endothelium* (an endothelial-to-hematopoietic lineage), three slices of the mouse gastrulation atlas (Pijuan-Sala et al. 2019: the full atlas as an 8,000-cell subsample to keep the ten-seed benchmark tractable, the E7.5 stage, and the erythroid sub-stream), and *Paul HSC*, *Mouse HSPC*, *Dentate Gyrus*, *PBMC68k*, *Endoderm differentiation* and *Ventral neuron*. All datasets undergo the identical preprocessing described below.

For *PBMC3k* and *Zeisel_brain*, we implemented a standardized preprocessing workflow using Scanpy [Wolf et al., 2018] to identify highly variable genes (HVGs) while preserving raw count structure. Briefly, genes were first filtered by detectability. Library-size normalization and $\log(1+x)$ transformation were performed solely to fit the Seurat v3 HVG model [Stuart et al., 2019]. The resulting HVG mask was then applied to the *original* raw count matrix, yielding a cells × HVGs matrix of untransformed counts. These HVG-filtered raw matrices served as input to PRISM-GEP and all baselines.

Table S5 lists the cell and gene counts of every dataset after preprocessing.

S3.1 Datasets preprocessing

We first filtered lowly detected genes, retaining only those expressed in at least m cells using `scanpy.pp.filter_genes` [Wolf et al., 2018]. Here we set $m = 3$ treating three as the minimal cohort size constituting a group (two cells constitute a pair rather than a group). Counts were normalized per cell to a fixed library size (`scanpy.pp.normalize_total`) and log-transformed (`scanpy.pp.log1p`) [Wolf et al., 2018, Luecken and Theis, 2019]. Highly variable genes (HVGs) were then selected with the Seurat v3 method (`flavor=seurat_v3`), using raw counts via `layer="counts"` and, where applicable, merging HVGs across batches via `batch_key` [Wolf et al., 2018, Stuart et al., 2019]. We request up to 5,000 highly variable genes (`n_top_genes`= 5000) for every dataset, a single setting applied uniformly rather than tuned per dataset. Where fewer genes survive the detectability filter, all of them are retained, which is why the gene counts in Table S5 range from 297 for the curated *BreastCancer* panel up to 5,000. The same vocabulary is then given to every method, so no method sees a gene set chosen to suit it. Analyses were performed in Scanpy with AnnData as the primary container [Wolf et al., 2018, Virshup et al., 2024].

Dataset	# cells	# genes
BreastCancer	2,748	297
PBMC3k	2,700	5,000
Zeisel_brain	3,005	5,000
Pancreas	3,696	5,000
Bonemarrow	5,780	5,000
Hemogenic Endo.	2,679	4,894
Gastrulation (full)	8,000	5,000
Gastrulation E7.5	7,202	5,000
Gastrulation Eryth.	9,815	5,000
Paul HSC	2,730	3,451
Dentate Gyrus	2,930	5,000
PBMC68k	8,000	5,000
Endoderm diff.	1,012	4,638
Ventral neuron	1,733	4,951
Mouse HSPC	1,919	4,823

Table S5 Cell and gene counts retained after preprocessing for each scRNA-seq dataset in this study. Larger counts simply mean more input signal. They are not a quality score.

S4 Baselines

We compare PRISM-GEP to representative methods from both scRNA-seq specific and text-corpus families. (i) scRNA-seq models: cNMF [Kotliar et al., 2019] and scHPF [Levitin et al., 2019]. (ii) Text-corpus models: NMF serves as a transparent parts-based baseline [Lee and Seung, 1999]. ProdLDA reparameterizes LDA within a variational autoencoder for improved optimization [Srivastava and Sutton, 2017]. LDA [Blei et al., 2003] is run via MALLET.

All baselines and PRISM-GEP were applied to the same raw, HVG-filtered matrices, matched for the number of factors/topics K, with identical gene vocabularies and multiple random seeds. All models (PRISM-GEP, LDA, cNMF, and scHPF) were executed using their respective official implementations with default parameter settings, as provided in their public repositories or documentation. Likewise, ProdLDA and NMF were run with default configurations. To ensure comparability across methods, the number of topics (or components) was fixed to $K_{\text{topics}} = 5$ for all models. No additional hyperparameter tuning was performed beyond these defaults. All six methods are unsupervised: each is fit solely on the gene-expression matrix, with no cell-type labels used in training. The five classical factorizations (PRISM-GEP, MALLET, NMF, cNMF, scHPF) are fit on the full set of cells of each dataset. ProdLDA, a neural (autoencoding) topic model trained by stochastic gradient descent, instead uses the standard 80/10/10 train/validation/test split of cells for fitting and early stopping. All coherence, coverage, and strength metrics are computed identically on the full expression matrix for every method, so the comparison is unaffected by this split.

Every stochastic method (PRISM-GEP, MALLET, scHPF, ProdLDA) is evaluated over ten random seeds. cNMF is likewise run over ten seeds. Its consensus step yields materially distinct solutions on two of the six additional datasets (Bonemarrow, Gastrulation) and converges to an effectively identical solution on the other four (verified md5-identical across seeds), so its zero-variance cells reflect genuine consensus convergence rather than a single-seed placeholder. NMF with its default initialization, the deterministic SVD-based

scheme (NNDSVD), which is fully deterministic and hence carries no seed variance, is additionally reseeded with random initialization (ten seeds). This restores seed variance on four datasets and leaves the qualitative ranking unchanged.

S5 Metrics

We assess topics (GEPs) using four complementary, biologically motivated criteria. First, *coherence* is quantified as the mean within-set Spearman correlation of gene expression. Second, *Gene Ontology Biological Process (GO-BP) coverage* measures the fraction of a program's annotated top-N genes that fall in significantly enriched biological processes. Third, *enrichment strength* is computed using a one-sided Fisher's exact test with Benjamini–Hochberg correction and reported as $-\log_{10} q$. Finally, we include an *LLM-based plausibility* score, which rates the alignment between a topic's gene list and candidate GO-BP descriptions, following Hu et al. [Hu et al., 2025].

S5.1 Gene-Set Quality Metrics

We assess the biological plausibility of inferred gene expression programs using three complementary criteria: (i) enrichment, the overrepresentation of curated pathways/functions (e.g., GO/KEGG/MSigDB) relative to a fixed gene universe. (ii) coherence, the within-gene-set co-variation of expression across cells. (iii) coverage, the fraction of a program's annotated genes that fall in significantly enriched pathways. Metrics are computed per program and then aggregated per model and dataset. We control the false discovery rate (FDR), the expected proportion of false positives among rejected hypotheses, using the Benjamini-Hochberg procedure and report BH-adjusted q-values [Benjamini and Hochberg, 1995].

Enrichment strength ($-\log_{10} q$).

Let U be the gene universe ($|U| = u$). For a program $S \subseteq U$ ($|S| = s$) and a curated pathway $P \subseteq U$ ($|P| = M$), with overlap $x = |S \cap P|$, we test overrepresentation using a one-sided hypergeometric (Fisher's exact) test:

$$p = \sum_{i=x}^{\min(s,M)} \frac{\binom{M}{i}\binom{u-M}{s-i}}{\binom{u}{s}}.$$

We then correct the collection of p-values across all tested pathways for S using FDR (Benjamini-Hochberg) to obtain q, and report the strength as the mean $-\log_{10} q$ over the significantly enriched pathways ($q < 0.05$), which is then averaged over the programs of a dataset (higher is better).

Coherence (mean within-set Spearman correlation).

Given the cells×genes expression matrix, compute Spearman correlations ρ_{ij} across cells for all gene pairs $i < j$, $i, j \in S$, and average:

$$\text{coherence} = \frac{2}{s(s-1)} \sum_{i<j,\ i,j \in S} \rho_{ij}.$$

Higher values indicate coordinated expression consistent with a functional program.

Coverage (pathway completeness).

For each GEP S we collect the set $\mathcal{P}^*$ of pathways significantly enriched for S (BH-FDR $q < 0.05$) and define Coverage as the fraction of S's annotated top-N genes that fall into the union of those enriched pathways:

$$\text{coverage}(S) = \frac{\left|\bigcup_{P \in \mathcal{P}^*} (S \cap P)\right|}{|\{\, g \in S : g \text{ is annotated in the library}\,\}|},$$

averaged over GEPs. Higher coverage means a program's top genes concentrate in coherent, significantly enriched pathways rather than scattering across the annotation. The qualitative method ranking is robust to reasonable alternative definitions of this quantity (public repository).

S5.2 LLM Confidence Score

To assess the biological relevance of gene sets derived from topic models, we follow the LLM-based evaluation protocol introduced by [Hu et al., 2025].[1] The core idea is to query a large language model (GPT-4) with each gene set and evaluate whether it can (1) identify a coherent biological process (BP) associated with the gene set, and (2) express high confidence in that association.

Prompt Design.

Each gene set is presented in a natural language prompt, instructing the model to infer a shared biological process based on the listed genes. Prompts are carefully crafted to be neutral and avoid leading the model toward specific functions. The model is then asked to (i) name the most likely BP, and (ii) rate its confidence on a scale from 0 to 1.

Scoring.

The model's textual output is manually inspected to verify whether the inferred BP matches a plausible biological function supported by external evidence (e.g., GO annotations). Confidence scores are recorded for each gene set and aggregated to assess overall coherence across topics.

Interpretation.

As shown in prior work, gene sets yielding low confidence often correspond to functionally inconsistent or noisy groups, whereas high-confidence predictions align with known biological pathways. Thus, the LLM confidence score serves as a proxy for functional coherence and interpretability of the gene sets.

We followed the protocol of Hu *et al.* [Hu et al., 2025] exactly, without any modifications.

S6 Results for Step (i): gene-program quality

S6.1 Aggregate robustness across the metric grid

We summarize the full 15 × 3 (dataset × metric) grid of Table S7 with three aggregate scores (Table S6), scoring PRISM-GEP against the four specialized methods exactly as in main-text Figure 3. BreastCancer's two enrichment-undefined entries are excluded, leaving 43 rankable entries. (i) *Mean rank*: each method's average position (1 best to 5 worst) over all entries. (ii) *Geometric composite*: within each dataset we rescale every metric to $[0, 1]$ (worst method 0, best 1), take the geometric mean across the three metrics, and average over datasets. Because a geometric mean falls to near zero as soon

[1] `https://www.ncbi.nlm.nih.gov/pmc/articles/PMC11725441`

Method	Mean rank↓ (95% CI)	Geom. composite↑	Worst-axis↑
scHPF	2.67 [2.19, 3.16]	0.120	0.535
**PRISM-GEP**	2.70 [2.37, 3.02]	0.463	0.502
NMF	2.79 [2.44, 3.12]	0.166	0.583
cNMF	3.26 [2.71, 3.80]	0.007	0.120
ProdLDA	3.58 [3.24, 3.91]	0.001	0.033

Table S6 Aggregate robustness over the full 15×3 grid of Table S7 (43 rankable entries, after excluding BreastCancer's two enrichment-undefined entries), scoring PRISM-GEP against the four specialized methods as in main-text Figure 3. The geometric composite and worst-axis score lie in $[0, 1]$ and drop toward zero if a method collapses on any single axis. Bracketed values are bootstrap 95% CIs over datasets.

as any one factor is near zero, this score is high only for a method that is good on all three metrics at once. (iii) *Worst-axis score*: each method's lowest rescaled metric, so a high value means the method never drops to the bottom on any single axis. The three views agree on one idea: a method scores well only if it holds up on every metric, not by winning one and collapsing another.

PRISM-GEP is the method that does. On mean rank it is level with scHPF and ahead of NMF, cNMF and ProdLDA, and its bootstrap interval is the narrowest in the panel, so its position is also the most stable across datasets. On the geometric composite it leads by a wide margin, because the two specialists each forfeit one metric entirely, cNMF on Coverage and ProdLDA on Coherence. On the worst-axis score it sits in the same cluster as NMF and scHPF, a little behind both, and far above the two specialists. Table S6 carries all three columns. Cell by cell, a two-sample Welch test over the ten seeds ($\alpha = 0.05$) makes PRISM-GEP best or statistically tied on 123 of the 172 pairwise comparisons.[2]

Table S7 gives the same comparison entry by entry, and it is where these aggregates come from. PRISM-GEP is never the worst method on any of the 43 entries, whereas every specialized method finishes last on several (ProdLDA 20, cNMF 14, scHPF 5, NMF 4, PRISM-GEP 0). Being consistently mid-to-high, rather than winning any single axis, is what separates it here.

S6.2 Keeping the informed prior fixed versus re-estimating it during training

MALLET fits LDA by collapsed Gibbs sampling, and during that sampling it can periodically pause to re-estimate its own Dirichlet hyperparameters from the token counts accumulated so far. How often it does this is set by a single value, the *optimization interval*. An interval of n means the hyperparameters are re-estimated once every n sampling iterations, and an interval of 0 turns the re-estimation off entirely. Re-estimation is a standard and generally helpful device in topic modeling: with the usual uninformative prior, letting the model retune its hyperparameters to the corpus tends to improve fit, because it moves an otherwise arbitrary default toward whatever the data actually support.

Our situation is different, because in PRISM-GEP the prior is the whole point. We replace the uninformative default with a vector-valued $\boldsymbol{\beta}$ estimated from the dataset's own gene co-expression

[2] Reproduce with `scripts/build_robustness_agg_15ds.py`, which re-derives the nine-dataset panel as a self-check before emitting the fifteen-dataset one. Per-method aggregates are written to `robustness_aggregates_9ds.csv` and `_15ds.csv`.

(Sec. 4). If we allowed MALLET to re-estimate its hyperparameters during training, the optimizer would gradually overwrite that informed $\boldsymbol{\beta}$ with values retuned to the running counts, and the fitted model would no longer reflect the prior we set out to evaluate. For the primary configuration we therefore fix the optimization interval to 0, a setting we call *opt0*, so that the informed $\boldsymbol{\beta}$ is used exactly as estimated. opt0 is the $\boldsymbol{\beta}$-faithful configuration reported throughout the main text, as the Hyperparameters paragraph of Sec. 5.1 states. It is also the configuration behind the robustness headline of main-text Figure 3, in which PRISM-GEP is never the worst method on any of the 43 rankable dataset × metric GO-BP entries.

We also report the re-estimating variant, with the interval set to 10 (opt10), as a companion configuration. It is strong in its own right: re-estimating the prior gives PRISM-GEP more first-place finishes (12 of the 43 entries, against 5 with the prior fixed) and a slightly better mean rank (2.51 versus 2.70), as Figure S5 shows. It also finishes last on 3 of the 43 entries rather than none. We keep the fixed-prior configuration primary in the main text for three reasons. First, our emphasis is robustness across every dataset and metric, which the fixed-prior configuration delivers. Second, the Step-(ii) gene-trajectory results are computed from the model before this re-estimation, so the fixed-prior configuration is the one consistent with the rest of the paper. Third, the two configurations differ by little. We therefore give the re-estimating variant here rather than in the main text.

S6.3 LLM-plausibility metric across the scored datasets

The fourth evaluation criterion, the GPT-4 plausibility score of Hu et al. [2025], is reported for BreastCancer, PBMC3k and Zeisel. We additionally scored the six other datasets over ten seeds with `gpt-4-turbo`, and Table S8 panel (B) collects *nine* LLM-scored datasets in one place so the full comparison can be read without cross-referencing the main text. The score depends on the scoring prompt, so we report two and show both for transparency: **(A)** a simplified bare-confidence prompt that asks only for a numeric score with the anchor "0.5 = some genes share function, others do not". Prompt **(B)** is the Hu et al. [2025] naming-then-confidence protocol, which first asks the model to name the single most likely shared process and then to rate its confidence, with gene symbols normalized to the organism convention (mouse Title-case, human upper-case). Prompt (A) returns a non-committal 0.5 for many developmental gene programs and is weakly discriminating, while prompt (B) is sharply discriminating. The conclusion is nonetheless the same under both. On the full four-metric aggregate (Coherence, Coverage, Strength, LLM) PRISM-GEP attains the best mean rank under *either* prompt (2.46 under A, 2.27 under B), and ProdLDA is last under both. On the LLM axis itself, under prompt (B), PRISM-GEP is first on all three summaries across the nine scored datasets, the mean, the median and the mean rank of six methods, and ahead of MALLET on each. PRISM-GEP, MALLET and scHPF form a tight top cluster within about 0.03, above cNMF and ProdLDA. Panel (B) of Table S8 and main-text Figure 4 carry the values. We nonetheless treat the LLM score as supporting, prompt-disclosed evidence rather than a primary metric.

S6.4 Cell-level label agreement

Table S9 reports the agreement of the dominant-GEP partition with published cell-type labels, alongside vanilla Leiden at matched cluster

Table S7 Gene-set quality (GO-BP) across the full benchmark (mean (std) over ten seeds, $K_{\text{topics}} = 5$, β-faithful configuration). **(a)** PRISM-GEP vs specialized factorizations: best per (dataset, metric) **bold**, second underlined. **(b)** PRISM-GEP vs uniform-prior MALLET: **bold** = Welch-significant winner ($p < 0.05$), the rest ties. "–" marks a GEP block with no significant enrichment.

(a) PRISM-GEP vs specialized methods

Dataset	PRISM-GEP Coh↑	PRISM-GEP Cov↑	PRISM-GEP Str↑	cNMF Coh↑	cNMF Cov↑	cNMF Str↑	scHPF Coh↑	scHPF Cov↑	scHPF Str↑	NMF Coh↑	NMF Cov↑	NMF Str↑	ProdLDA Coh↑	ProdLDA Cov↑	ProdLDA Str↑
BreastCancer	.280 (.001)	—	—	.269 (.000)	.384 (.000)	**1.84 (0.00)**	.267 (.005)	—	—	**.286 (.000)**	—	—	.266 (.025)	**.600**	1.34
PBMC3k	**.361 (.011)**	.904 (.024)	4.69 (0.46)	.350 (.000)	.813 (.000)	4.04 (0.00)	.320 (.014)	.935 (.027)	**5.09 (0.49)**	.293 (.000)	**.944 (.000)**	4.58 (0.00)	.250 (.042)	.899 (.079)	2.89 (0.94)
Zeisel brain	.502 (.033)	.681 (.053)	1.83 (0.07)	.542 (.000)	.509 (.000)	**2.13 (0.00)**	.532 (.035)	.712 (.044)	1.90 (0.04)	**.579 (.000)**	.663 (.000)	1.86 (0.00)	.455 (.049)	**.890 (.038)**	1.46 (0.07)
Hemogenic Endo.	.618 (.001)	.938 (.007)	6.33 (0.10)	.375 (.000)	.724 (.000)	2.02 (0.00)	**.626 (.012)**	**.950 (.026)**	**6.95 (0.49)**	.564 (.000)	.935 (.000)	5.84 (0.00)	.336 (.099)	.913 (.102)	3.05 (0.67)
Pancreas	.355 (.035)	.713 (.036)	**4.69 (0.70)**	**.491 (.000)**	.494 (.000)	1.72 (0.00)	.296 (.031)	.760 (.042)	4.18 (0.51)	.343 (.000)	.682 (.000)	4.57 (0.00)	.015 (.011)	**.762 (.142)**	1.39 (0.06)
Gastrulation E7.5	.636 (.009)	**.899 (.008)**	4.74 (0.50)	.385 (.001)	.656 (.000)	1.81 (0.00)	**.645 (.006)**	.886 (.018)	5.16 (0.35)	.622 (.000)	.850 (.000)	**5.60 (0.00)**	.308 (.061)	.877 (.065)	2.18 (0.47)
Gastrulation (full)	**.414 (.012)**	.769 (.075)	3.18 (0.13)	.385 (.000)	.674 (.012)	2.91 (0.01)	.410 (.010)	.737 (.041)	3.03 (0.17)	.400 (.000)	.845 (.000)	**3.62 (0.00)**	.262 (.013)	**.870 (.090)**	1.51 (0.08)
Gastrulation Eryth.	.445 (.001)	.746 (.011)	1.75 (0.01)	**.458 (.000)**	.543 (.000)	**2.04 (0.00)**	.422 (.013)	.784 (.032)	1.76 (0.04)	.347 (.000)	.856 (.000)	1.71 (0.00)	.331 (.053)	**.857 (.086)**	1.54 (0.05)
Bonemarrow	**.313 (.005)**	.467 (.066)	2.07 (0.19)	.301 (.010)	.435 (.011)	1.80 (0.12)	.296 (.011)	.491 (.098)	1.95 (0.06)	.282 (.000)	.508 (.000)	**2.14 (0.00)**	.021 (.006)	**.721 (.075)**	1.47 (0.16)
Paul HSC	.577 (.010)	.662 (.067)	2.55 (0.38)	.394 (.000)	.567 (.000)	1.96 (0.00)	**.589 (.018)**	.708 (.046)	**2.66 (0.17)**	.512 (.000)	.579 (.000)	2.56 (0.00)	.349 (.075)	**.784 (.138)**	1.51 (0.16)
Dentate Gyrus	.360 (.007)	.710 (.074)	3.61 (0.38)	**.429 (.018)**	.679 (.020)	1.68 (0.04)	.340 (.022)	.735 (.052)	3.27 (0.44)	.373 (.000)	**.826 (.000)**	**5.41 (0.00)**	.263 (.031)	.801 (.092)	1.67 (0.11)
PBMC68k	.088 (.003)	.888 (.076)	8.30 (0.79)	**.209 (.000)**	.854 (.000)	4.27 (0.00)	.084 (.007)	**.922 (.036)**	**9.02 (1.03)**	.079 (.003)	.920 (.001)	6.82 (0.03)	.107 (.022)	.898 (.097)	4.59 (1.10)
Endoderm diff.	.247 (.006)	.544 (.148)	1.62 (0.08)	**.608 (.072)**	.598 (.022)	1.63 (0.03)	.218 (.004)	.482 (.144)	**1.70 (0.11)**	.319 (.000)	.699 (.000)	1.44 (0.00)	.370 (.027)	**.920 (.076)**	1.46 (0.04)
Ventral neuron	.140 (.003)	.765 (.014)	1.78 (0.05)	**.400 (.001)**	**.945 (.000)**	**2.32 (0.00)**	.126 (.008)	.719 (.043)	1.62 (0.11)	.180 (.000)	.729 (.000)	1.50 (0.00)	.282 (.045)	.944 (.051)	1.50 (0.07)
Mouse HSPC	.199 (.006)	.535 (.037)	1.87 (0.22)	**.374 (.001)**	.555 (.026)	2.35 (0.24)	.193 (.006)	.569 (.101)	1.79 (0.17)	.233 (.000)	.302 (.000)	**3.46 (0.00)**	.249 (.035)	**.903 (.054)**	1.58 (0.08)

(b) PRISM-GEP vs uniform-prior MALLET

Dataset	PRISM-GEP Coh↑	PRISM-GEP Cov↑	PRISM-GEP Str↑	MALLET Coh↑	MALLET Cov↑	MALLET Str↑
BreastCancer	.280 (.001)	—	—	.276 (.006)	—	—
PBMC3k	.361 (.011)	.904 (.024)	4.69 (0.46)	.364 (.010)	.908 (.021)	4.61 (0.45)
Zeisel brain	.502 (.033)	.681 (.053)	1.83 (0.07)	.527 (.034)	.685 (.034)	1.85 (0.06)
Hemogenic Endo.	.618 (.001)	**.938 (.007)**	6.33 (0.10)	.615 (.007)	.900 (.041)	6.31 (0.41)
Pancreas	.355 (.035)	.713 (.036)	4.69 (0.70)	.341 (.036)	.701 (.054)	4.36 (0.56)
Gastrulation E7.5	.636 (.009)	.899 (.008)	4.74 (0.50)	.640 (.008)	.885 (.020)	5.04 (0.52)
Gastrulation (full)	.414 (.012)	.769 (.075)	3.18 (0.13)	.410 (.015)	.743 (.050)	3.23 (0.25)
Gastrulation Eryth.	.445 (.001)	.746 (.011)	1.75 (0.01)	**.450 (.006)**	.741 (.014)	1.76 (0.02)
Bonemarrow	.313 (.005)	.467 (.066)	2.07 (0.19)	.316 (.005)	.449 (.040)	2.22 (0.15)
Paul HSC	.577 (.010)	.662 (.067)	2.55 (0.38)	**.588 (.005)**	.626 (.058)	2.52 (0.16)
Dentate Gyrus	.360 (.007)	.710 (.074)	3.61 (0.38)	.358 (.013)	.688 (.074)	3.53 (0.39)
PBMC68k	.088 (.003)	.888 (.076)	8.30 (0.79)	.088 (.004)	.888 (.041)	8.40 (0.63)
Endoderm diff.	.247 (.006)	.544 (.148)	1.62 (0.08)	.245 (.013)	.550 (.160)	1.64 (0.10)
Ventral neuron	**.140 (.003)**	.765 (.014)	1.78 (0.05)	.132 (.010)	.739 (.054)	1.74 (0.08)
Mouse HSPC	.199 (.006)	.535 (.037)	1.87 (0.22)	.200 (.007)	.564 (.052)	1.95 (0.20)

count. At matched cluster count (k=5) Leiden attains a higher ARI on 8 of the 13 labeled datasets and PRISM-GEP on 5. The PRISM-GEP wins are Zeisel (0.854 vs 0.834, *a single seed*), full Gastrulation (0.152 vs 0.130), Gastrulation E7.5 (0.274 vs 0.248), Dentate Gyrus (0.504 vs 0.501) and Endoderm (0.400 vs 0.295). This split is expected for a soft decomposition rather than a hard-clustering method. Leiden leads more broadly when each method is given its own best k (the k=#types column). We therefore frame PRISM-GEP as a soft decomposition, not a competitive hard clusterer. Zeisel, PBMC3k and five further datasets are single-seed (seed 0). The other six are ten-seed means. PBMC3k's published labels are themselves named Louvain clusters (the `scanpy` tutorial annotation), so Leiden's lead there is expected and not an independent comparison. We retain the row for completeness. The K_{topics}=# (best-k) PRISM models were trained only for eight of the datasets, so the other five rows show $--$ there.

S6.5 Cell-cell neighborhood ablation

We swapped Stage A's incidence matrix H among nine neighborhood constructions and reran the full pipeline on the six additional datasets over ten seeds (five on the two heaviest). The constructions are kNN at $k \in \{5, 15, 50\}$, the *real* Metacells [Baran et al., 2019] (`metacells 0.9.5`) and SEACells [Persad et al., 2023] (`SEACells 0.3.3`) implementations, and a Milo-like [Dann et al., 2022] overlapping-kNN surrogate. Metacells was run at the recommended ∼75 cells/metacell [Baran et al., 2019], plus a 30/100 small/large bracket. Table S10 reports the discriminating metric, Coverage. The graph choice is within seed noise on four datasets (Pancreas, Hemogenic, E7.5, Erythroid, with spread ≤ 0.034). On Bonemarrow the default kNN is the best construction (by 0.017), and on the full Gastrulation atlas a Metacells coarse-graining narrowly exceeds the default by 0.056 (spread 0.085). No construction systematically beats the default kNN, confirming that the downstream signal is dominated by Stages B–D rather than the Stage-A graph.

Two reading notes for Table S10. The Hemogenic column is saturated near its ceiling (∼0.974, spread below seed noise) and is left unmarked. Values that tie at three decimals are ranked by the full-precision mean (e.g. in Gastrulation Erythroid the runner-up Metacells(30)= 0.8442 edges Metacells(100)= 0.8437, which also prints as 0.844).

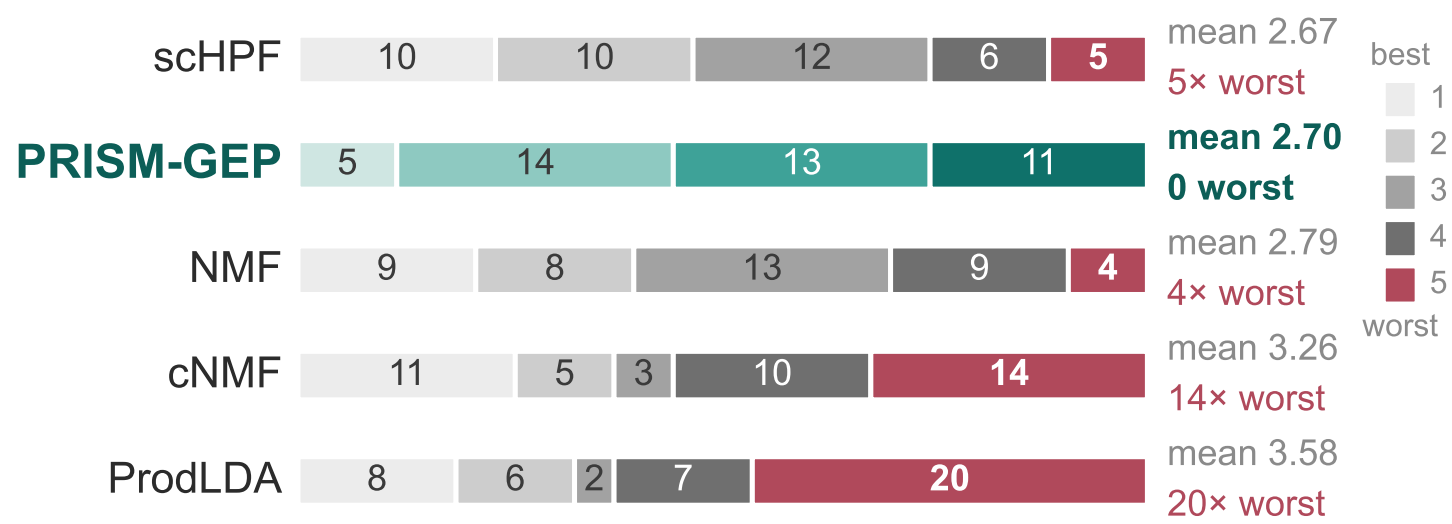

(a) Prior fixed (opt0), the configuration used throughout the main text

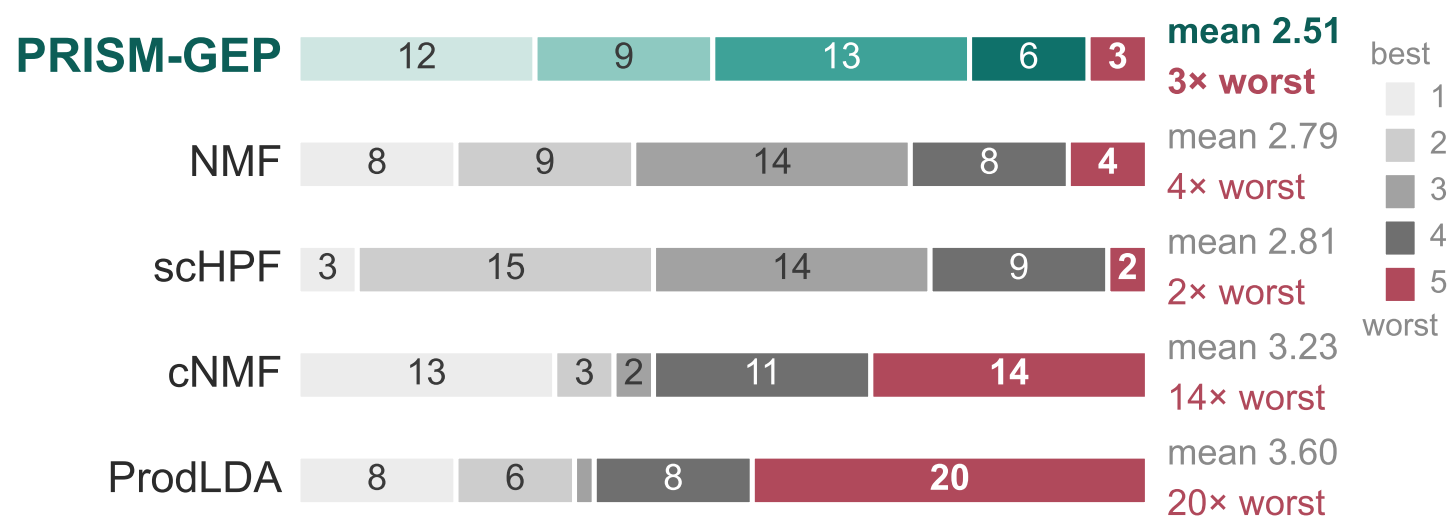

(b) Prior re-estimated every 10 iterations (opt10)

Figure S5 GO-BP rank distribution under the two hyperparameter settings, over the 43 rankable (dataset × metric) entries. **(a)** With the informed prior held fixed, PRISM-GEP finishes last on none of the 43 entries. **(b)** Re-estimating the prior buys more first-place finishes (12 of 43 against 5) and a slightly better mean rank (2.51 against 2.70), but costs three last-place finishes. Never finishing last is therefore specific to the fixed-prior configuration, which is why that one is primary.

Alt text: (a) Five horizontal stacked bars, one per method: scHPF, PRISM-GEP, NMF, cNMF and ProdLDA. Each bar is split into rank 1 to 5 segments with counts printed in the wider ones, grey with rank 5 in red for the four specialized methods and teal for PRISM-GEP, whose bar has no red segment. Mean rank and times worst are printed at the right. (b) The same layout with the rows reordered to PRISM-GEP, NMF, scHPF, cNMF and ProdLDA, and PRISM-GEP's bar ending in a small red segment of 3.

S6.6 PRISM-GEP versus MALLET

PRISM-GEP and vanilla MALLET differ in exactly one component, the topic–gene prior, so comparing them isolates what the informed $\boldsymbol{\beta}$ contributes. We compare both models at optimize-interval 0, the $\boldsymbol{\beta}$-faithful configuration of Sec. S6.2, where the informed prior is used exactly as estimated. PRISM-GEP can equally be run with hyperparameter optimization enabled, but each optimization step re-estimates the prior from the running token counts and dilutes the co-expression structure it started from. The effect is measurable. The prior's Coverage advantage over MALLET is largest at opt0 and roughly halves once optimization is enabled, a mean gain of +0.011 at opt0 against +0.005 at opt10, and on Hemogenic Endothelium a gain of +0.038 that collapses to +0.002. In the limit of heavy optimization PRISM-GEP therefore converges toward vanilla MALLET, which is why we report the $\boldsymbol{\beta}$-faithful opt0 as the primary configuration.

On the count-based GO-BP metrics the two models are a statistical tie. Coherence is essentially identical across datasets, with per-dataset differences of at most 0.025 in either direction, a mean absolute difference of 0.006 and a signed mean of −0.001 in MALLET's favor. Strength differs more per dataset but without a consistent direction, a large gain on one dataset offset by a loss on another, so it too is a wash. The full per-dataset grid is Table S7(b).

The informed prior does leave a consistent mark, and it does so where the data's own co-expression is most informative. On Coverage, the breadth of a program's biological explainability, the informed prior improves clearly over the uniform prior on the focused-lineage datasets: Hemogenic Endothelium (+0.038), the mouse gastrulation atlas (+0.026), ventral neuron differentiation (+0.026), Dentate Gyrus (+0.022), and Pancreas (Coverage +0.012, Strength +0.34). These are the transcriptomes whose gene co-expression is most sharply structured, which is exactly where a co-expression-derived prior is expected to help. We report these cases to characterize the regime in which the prior adds value, not as a claim of uniform superiority. The complete grid, including the datasets on which the two models tie, is Table S7(b).

On the interpretability axis the prior produces a clean aggregate win. On the GPT-4 plausibility score PRISM-GEP attains the best mean, median, and mean rank across nine scored datasets (0.879, 0.886, and 1.72 of six methods, ahead of MALLET's 0.861, 0.882, and 2.61), summarized in Table S8 and Figure 4.

Finally, the same gene–gene geometry that defines the prior yields the intrinsic within-program gene ordering of Step (ii) at no additional cost, a capability vanilla MALLET does not provide. At matched GO-BP quality PRISM-GEP is therefore preferable to its uniform-prior parent. It recovers the same programs while adding a data-derived prior, a Coverage gain where co-expression is focused, the plausibility win, and the gene ordering.

S7 Results for Step (ii): gene trajectories

S7.1 Gene-trajectory recovery

At this marker scale no method is statistically separable from any other, so gene-trajectory recovery is a robustness result rather than a ranking. Table S11 shows why. Step-(ii) orders each program's genes along a one-dimensional pseudotime, and the table measures how closely that order matches a dataset's canonical marker progression. Every stochastic entry is a mean over ten model fits, matching main-text Table 1a, and the table keeps the two uncertainties that bear on it in separate columns. s_{seed} is the spread over those ten fits. The

**(A) Simplified bare-confidence prompt** *(six added datasets)*						
Dataset	PRISM-GEP	MALLET	NMF	cNMF	scHPF	ProdLDA
Hemogenic Endo.	**.678**†	.640	.664	.600	.650	.388
Pancreas	.500	.500	.560	**.600**	.500	.290
Gastrulation E7.5	.538†	.536	.508	**.540**	.530	.352
Gastrulation (full)	.500	.500	.500	**.590**	.504	.360
Gastrulation Eryth.	.522†	.520	.500	**.560**	.504	.382
Bonemarrow	.500	.500	.500	.500	.500	.390
mean rank ↓	2.67	3.50	3.50	**2.00**	3.33	6.00
mean (std)	.540 (.069)	.533 (.055)	.539 (.066)	**.565 (.040)**	.531 (.059)	.360 (.038)
**(B) Naming-then-confidence prompt [Hu et al., 2025]** *(nine LLM-scored datasets)*						
Dataset	PRISM-GEP	MALLET	NMF	cNMF	scHPF	ProdLDA
BreastCancer	**.832**†	.792	.788	.800	.771	.760
PBMC3k	.900†	.839	.659	**.902**	.833	.495
Zeisel brain	**.935**†	.892	.667	.933	.898	.595
Hemogenic Endo.	**.939**	**.939**	.907	.863	.885	.442
Pancreas	.791	**.808**	.798	.707	.786	.073
Gastrulation E7.5	**.916**†	.914	.822	.623	.910	.408
Gastrulation (full)	.878	**.882**	.867	.866	.868	.466
Gastrulation Eryth.	.886†	.885	.866	.719	**.894**	.525
Bonemarrow	.838†	.797	.815	**.860**	.805	.114
mean rank ↓	**1.72**	2.61	3.78	3.44	3.44	6.00
mean (std)	**.879 (.050)**	.861 (.054)	.799 (.086)	.808 (.104)	.850 (.052)	.431 (.218)

Table S8 LLM-plausibility score (`gpt-4-turbo`, mean over 10 seeds) under two prompts. Panel (A) covers the six datasets added in revision, the only ones for which the simplified prompt was run. Panel (B) is the faithful Hu et al. [2025] naming protocol that we rely on, and it collects the **nine** LLM-scored datasets in one place: BreastCancer, PBMC3k and Zeisel together with the six added. Best per row is **bold**, runner-up underlined, and † marks each entry where PRISM-GEP exceeds MALLET. *mean (std)* is each method's mean and standard deviation across that panel's datasets. Prompt (A) is weakly discriminating (a floor of non-committal 0.5 scores on developmental programs). Interpretation is in the text.

Dataset	#types	PRISM K_{topics}=5	Leiden k=5	PRISM K_{topics}=#	Leiden k=#
Zeisel_brain	7	**.854**	.834	.829	**.879**
PBMC3k	8	.537	**.824**	.443	**.625**
Pancreas	8	.399	**.641**	.438	**.578**
Bonemarrow	10	.348	.365	.491	**.617**
Gastrulation	9	.152	.130	.151	**.155**
Gastrulation E7.5	27	.274	.248	**.399**	.354
Gastrulation Eryth.	7	.308	.359	.266	**.458**
Hemogenic Endo.	4	.084	.179	.125	**.228**
Dentate Gyrus	14	**.504**	.501	–	.575
Endoderm Diff.	2	**.400**	.295	–	.740
Paul15	19	.291	**.297**	–	.289
PBMC68k	11	.155	**.199**	–	.160
Ventral Neuron	4	.205	**.288**	–	.195

Table S9 ARI↑ of the dominant-GEP partition vs published cell-type labels, with vanilla Leiden at matched cluster count. Best per row is in **bold**. PRISM-GEP is a soft decomposition rather than a hard clusterer, so the aim is competitive agreement, not agreement exceeding Leiden (interpretation in the text).

bracketed interval is a marker bootstrap, a different axis entirely. Marker sets are small (5–20 genes), so for that interval we resample markers with replacement (B=5000), drawing one of the ten fits per replicate so that the interval averages over fits exactly as the point estimate does. The intervals of PRISM-GEP Step-(ii), GeneTrajectory and the root-supervised DPT-weighted-mean baseline overlap on every dataset without exception. Hemogenic Endothelium is the near miss. At a single fit PRISM-GEP's interval cleared GeneTrajectory's extracted trajectory, but once the bootstrap averages over all ten fits the two meet, [.758, .974] against [.017, .774], so no separation in this table survives. PRISM-GEP clears the expression-magnitude floor on eight of the nine datasets, most clearly on Bonemarrow haematopoiesis, where that floor is at its lowest of any dataset, and then on Pancreas, with the multi-lineage Gastrulation atlas and Paul15 close behind each other. It falls below that floor only on the five-marker Dentate Gyrus. On the near-saturated Erythroid lineage the floor is itself high (.834), so every method's margin there is small.

Table S11 gives the per-dataset values behind the body's gene-trajectory summary (Figure 6), covering all four additionally-scored lineages: Paul15 (myeloid/erythroid), DG (dentate-gyrus neurogenesis), Endo. (definitive-endoderm) and § *Gastrulation E7.5* (E75). E75 is a case where our method loses, and we report it rather than drop it silently. Its canonical order collapses divergent terminal fates (cardiac *Gata4/Hand1/Tbx5*, endoderm *Sox17*, neuroectoderm *Pax6*) into a single terminal rank, so the reference is a branching tree flattened onto one axis and PRISM-GEP's single-lineage Step (ii) is mismatched to it. In this table PRISM-GEP Step (ii) scores 0.345 on E75, its ten-seed mean, which sits above the expression-magnitude floor of 0.254 in this table and above the chance level of 0.227 in main-text Table 1a. That single number hides the behavior that matters. Across the ten fits PRISM-GEP ranges from 0.025 to 0.549 with a standard deviation of 0.238, against 0.013 on Hemogenic Endothelium, and four of the ten fall below the chance level, so we read E75 as a null result rather than a recovery. Those ten values do not scatter, they split into two groups, four near 0.07 and six near 0.53, separated by a gap about four times the spread within either group, where seven of Hemogenic Endothelium's ten fits return an identical value. The instability is spectral. E7.5's gene–gene diffusion operator has near-degenerate leading eigenvalues (the ratio λ_3/λ_2 averages 0.56 across the ten fits and ranges from 0.44 to 0.73, where Hemogenic Endothelium gives 0.016), so which axis occupies the first diffusion component is close to a coin flip. Three methods clear both floors more stably than we do (DPT 0.549, GT-EV 0.553, GT-best 0.573). Two of the three, DPT and GT-EV, are scored on the same 14 markers as PRISM-GEP, so they recover this order better than we do and we report that as a loss. The GT-best value is the one number here that is not comparable to the others, since its best-matching trajectory covers only 5 to 8 of the 14 canonical markers depending on the fit, where GT-extract covers 13 and every other method is scored on all 14, and a Spearman correlation over so few points carries very wide uncertainty. E75 marks a boundary of where a single-lineage gene ordering applies. The root-supervised DPT baseline is omitted (undefined on Paul15, Dentate Gyrus and Endoderm), and GeneTrajectory's native best trajectory is undefined on Gastrulation and DG (too few markers in any single extracted trajectory). On the other eight datasets PRISM-GEP holds the best mean (0.803). Including E75, it holds the best mean among the single-ordering methods (0.752 against GeneTrajectory-EV's 0.675) and also the best median (0.781 against 0.776). The median margin is a fiftieth of the metric's range and far inside the marker-bootstrap width, so it separates nothing on its own. No single method dominates. The body figure makes the same point visually. PRISM-GEP has the tightest cluster among the single-ordering methods and its one low outlier is *Gastrulation E7.5*, while GeneTrajectory's eigenvector ordering is bimodal (strong on several datasets, collapsing on Paul15 and the Erythroid lineage), which is what opens the gap between the two means.

The recovered orderings are shown directly, so the reader can judge them by eye rather than through a single number. Against

H family	Panc.	Bonem.	Hemog.	E7.5	Gastr.	Eryth.
kNN (k=15, def.)	0.747 (0.042)	**0.533 (0.101)**	0.974 (0.000)	0.883 (0.004)	0.770 (0.060)	0.841 (0.009)
kNN (k=5)	0.752 (0.023)	0.475 (0.103)	0.974 (0.000)	**0.884 (0.004)**	0.741 (0.004)	0.843 (0.007)
kNN (k=50)	0.753 (0.028)	0.484 (0.092)	0.974 (0.000)	0.884 (0.009)	0.809 (0.066)	0.843 (0.011)
Metacells (30)	0.758 (0.023)	0.464 (0.071)	0.974 (0.001)	0.881 (0.004)	0.760 (0.038)	0.844 (0.011)
Metacells (75, rec.)	0.735 (0.030)	0.516 (0.107)	0.974 (0.000)	0.881 (0.005)	0.772 (0.071)	0.843 (0.007)
Metacells (100)	**0.767 (0.028)**	0.450 (0.049)	0.974 (0.001)	0.878 (0.001)	**0.826 (0.056)**	0.844 (0.011)
SEACells	0.733 (0.027)	0.504 (0.084)	0.974 (0.000)	0.883 (0.004)	0.761 (0.039)	0.843 (0.007)
Milo-like (p=.10)	0.759 (0.036)	0.469 (0.082)	0.974 (0.000)	0.880 (0.004)	0.805 (0.060)	**0.847 (0.012)**
Milo-like (p=.25)	0.761 (0.029)	0.471 (0.078)	0.973 (0.004)	0.882 (0.005)	0.799 (0.066)	0.841 (0.009)

Table S10 Coverage↑ (mean (std), 10 seeds, or 5 for the two heaviest datasets) under nine Stage-A neighborhood constructions. Per column, **best** is bold and the runner-up is underlined. These runs enable MALLET's hyperparameter re-estimation (optimize-interval 10) rather than the optimize-interval 0 of the primary tables, so read the values against each other and not against Table S7.

an *independent* cell axis (a Slingshot pseudotime PRISM-GEP never sees), a correct gene order produces a diagonal "activation cascade". PRISM-GEP produces one as clean as GeneTrajectory's on Pancreas, Gastrulation and the Erythroid lineage (Figures S6a–S6d). Its own within-program trajectories for five representative datasets are in Figure S9, and on GeneTrajectory's own synthetic simulators, where a ground-truth gene order exists, PRISM-GEP reconstructs the cascade from expression alone (rank agreement $|\rho| \in [0.81, 0.98]$, Figures S11a–S11b).

S7.2 GeneTrajectory configuration sensitivity

GeneTrajectory's marker-recovery score depends strongly on two resourcing knobs: the number of panel genes used to build the gene–gene graph and the number of metacells used to estimate gene diffusion. We report one uniform configuration for every dataset, 500 panel genes and 250 metacells, throughout Table S11. Table S12 places that setting next to one alternative per dataset so the sensitivity is visible directly. We do *not* tune the configuration to the score, and the sweep discloses what that costs us. On three of the five datasets swept, the configuration we do *not* report is the better one for GeneTrajectory. On Pancreas the lighter 300/150 setting reaches 0.83 against the reported 0.65, which would place GeneTrajectory above PRISM-GEP. On Hemogenic Endothelium the heavier 700/350 setting reaches 0.72 against the reported 0.35. On Paul15 the heavier 700/350 setting reaches 0.064 against the reported 0.038, a gap that is negligible at this marker-set scale. The uniform setting is therefore conservative against, not for, our method wherever the difference is material.

The marker-bootstrap intervals in Table S11 overlap for every pair of learned methods on every dataset, with no exception. Hemogenic Endothelium is worth stating explicitly because it was the one near miss. There PRISM-GEP Step-(ii) has bootstrap mean 0.912 with interval $[0.758, 0.974]$ against 0.368 with interval $[0.017, 0.774]$ for GeneTrajectory's extracted trajectory, and those two intervals do meet, though only just. Three things would each have to hold for a superiority claim here, and none does. The intervals touch once the bootstrap averages over all ten fits rather than the single fit we previously reported. At GeneTrajectory's heavier 700/350 setting the same extracted ordering reaches 0.722 with bootstrap mean 0.701 and interval $[0.279, 0.947]$, comfortably overlapping ours. Its own eigenvector ordering here overlaps us too, at $[0.707, 0.972]$. The conclusion is statistical indistinguishability at this marker-set scale on every dataset, and the point-estimate ordering is descriptive only.

S7.3 Gene-embedding comparison (scGPT)

No single gene representation dominates at this marker scale. PRISM-GEP's dataset-intrinsic gene similarity is statistically indistinguishable from scGPT's contextual embeddings and ahead of its static ones on most datasets, without any pretraining. We show this by replacing that similarity with scGPT [Cui et al., 2024] embeddings, static and contextual, inside the Step-(ii) ordering and scoring against canonical markers. We run the comparison on the nine datasets that carry a canonical, ordinal marker sequence to score against, the same roster used for gene-trajectory recovery. The remaining datasets lack a ground-truth gene ordering, so a |Spearman ρ| against canonical order is undefined there. The scGPT-embeddable-intersection group, main-text Table 1b, re-scores PRISM-GEP on the strict marker intersection scGPT can embed, so that no representation gains from a larger marker set. Every method that carries a seed is reported as a mean over ten model seeds with the across-seed standard deviation beside it. Static scGPT and log1p are deterministic on this path, have no seed dimension, and are reported as single values.

Against the static embedding PRISM-GEP leads, averaging .775 against .593, winning six of the nine datasets and tying on one. Its clearest margins are where scGPT's human-only vocabulary is a handicap: Paul15 (.949 against .316), Hemogenic Endothelium (.928 against .064) and Gastrulation (.751 against .342). Its two losses are Dentate Gyrus, where static scGPT reaches .975 against .585, and *Gastrulation E7.5* (.414 against .326). Per-dataset values are in main-text Table 1b.

The contextual pass is a closer comparison and a different one. It is not separable from PRISM-GEP in aggregate, .768 against .775 (paired t p=.95, Wilcoxon p=.74), and it is ahead on five of the nine datasets to PRISM-GEP's three. On Hemogenic Endothelium it leads outright, .954 against .928. It is also close to deterministic in practice, returning an identical value on all ten seeds on six of the nine datasets, so its standard deviations are genuinely near zero rather than unreported.

Two representation-free baselines on the full marker set put those numbers in scale. The random row is a chance level rather than a floor. It is the mean over 10,000 random orderings per dataset, not a single draw, because a single draw is a poor stand-in for one: on Dentate Gyrus one draw returns .616 where the chance level is .417, a draw that sits at roughly the 73rd percentile of that null and would overstate the floor by nearly half. Against the chance level PRISM-GEP is ahead on all nine datasets, but on Dentate Gyrus (.585 vs .417) and *Gastrulation E7.5* (.345 vs .227) the margin is under one

Dataset	Method	$\lvert\rho\rvert$	s_{seed}	boot. mean	95% $\text{CI}_{\text{marker}}$	n
Pancreas	PRISM-GEP Step (ii)	.844	.057	.824	[.494, .962]	15
	GeneTrajectory (best traj.)	.365	.000	—	—	—
	GeneTrajectory (extract)	.648	.004	—	—	—
	GeneTrajectory (EV)	.776	.000	.751	[.347, .928]	15
	DPT-weighted-mean†	.912	.000	.894	[.741, .966]	15
	Expression magnitude	.315	.000	.346	[.019, .765]	15
Gastrulation	PRISM-GEP Step (ii)	.781	.039	.751	[.317, .955]	11
	GeneTrajectory (best traj.)	—	—	—	—	—
	GeneTrajectory (extract)	—	—	—	—	—
	GeneTrajectory (EV)	.916	.000	.892	[.681, .980]	11
	DPT-weighted-mean†	.802	.000	.788	[.320, .981]	11
	Expression magnitude	.354	.000	.394	[.018, .911]	11
Gastr. Erythroid	PRISM-GEP Step (ii)	.930	.012	.901	[.629, .994]	8
	GeneTrajectory (best traj.)	.930	.032	—	—	—
	GeneTrajectory (extract)	.957	.020	.938	[.784, 1.000]	8
	GeneTrajectory (EV)	.222	.000	.457	[.084, .962]	8
	DPT-weighted-mean†	.964	.000	.944	[.788, 1.000]	8
	Expression magnitude	.865	.000	.834	[.342, 1.000]	8
Hemogenic Endo.	PRISM-GEP Step (ii)	.928	.013	.912	[.758, .974]	15
	GeneTrajectory (best traj.)	.886	.014	—	—	—
	GeneTrajectory (extract)	.324	.068	.368	[.017, .774]	15
	GeneTrajectory (EV)	.917	.000	.900	[.707, .972]	15
	DPT-weighted-mean†	.788	.000	.765	[.458, .928]	15
	Expression magnitude	.734	.000	.702	[.391, .899]	15
Bonemarrow	PRISM-GEP Step (ii)	.903	.004	.886	[.734, .959]	20
	GeneTrajectory (best traj.)	.595	.028	—	—	—
	GeneTrajectory (extract)	.239	.119	—	—	—
	GeneTrajectory (EV)	.817	.006	.795	[.594, .916]	20
	DPT-weighted-mean†	.919	.000	.903	[.755, .969]	20
	Expression magnitude	.058	.000	.208	[.008, .563]	20
Paul15	PRISM-GEP Step (ii)	.684	.043	.672	[.085, .994]	8
	GeneTrajectory (best traj.)	.038	.000	—	—	—
	GeneTrajectory (extract)	.038	.000	.393	[.013, .877]	8
	GeneTrajectory (EV)	.140	.000	.425	[.075, .889]	8
	DPT-weighted-mean†	—	—	—	—	—
	Expression magnitude	.255	.000	.396	[.013, .991]	8
Dentate Gyrus	PRISM-GEP Step (ii)	.585	.043	.642	[.053, 1.000]	5
	GeneTrajectory (best traj.)	—	—	—	—	—
	GeneTrajectory (extract)	.738	.000	—	—	—
	GeneTrajectory (EV)	.975	.000	.965	[.745, 1.000]	5
	DPT-weighted-mean†	—	—	—	—	—
	Expression magnitude	.667	.000	.718	[.108, 1.000]	5
Endoderm	PRISM-GEP Step (ii)	.765	.030	.741	[.164, .990]	7
	GeneTrajectory (best traj.)	.577	.000	—	—	—
	GeneTrajectory (extract)	.472	.000	.537	[.060, .935]	7
	GeneTrajectory (EV)	.756	.000	.735	[.163, .990]	7
	DPT-weighted-mean†	—	—	—	—	—
	Expression magnitude	.661	.000	.656	[.063, .990]	7
Gastrulation E7.5§	PRISM-GEP Step (ii)	.345	.238	.380	[.019, .737]	14
	GeneTrajectory (best traj.)	.573	.182	—	—	—
	GeneTrajectory (extract)	.274	.060	—	—	—
	GeneTrajectory (EV)	.553	.018	.556	[.050, .959]	14
	DPT-weighted-mean†	.549	.000	.549	[.045, .953]	14
	Expression magnitude	.254	.000	.315	[.016, .737]	14

Table S11 Gene-trajectory recovery on the nine datasets with a canonical marker order. Columns, left to right. $|\rho|$ is the absolute Spearman correlation between a method's recovered gene order and the canonical order, averaged over *ten model fits*, the same ten reported in main-text Table 1a. s_{seed} is the standard deviation over those ten fits, so it measures how much the fit matters, and it is exactly $.000$ for the two deterministic baselines. boot. mean and 95% $\text{CI}_{\text{marker}}$ come from a *marker* bootstrap, a different axis of uncertainty: marker sets are small, so we resample the markers with replacement ($B{=}5000$), drawing one of the ten fits per replicate so the interval rests on the same ten-fit footing as the point estimate. A bracket here therefore never refers to seed spread, which is the s_{seed} column. The bootstrap mean drifts from the point estimate on the smallest marker sets, so both are shown. n is the marker-panel size (5 to 20 genes). †the root-supervised DPT-weighted-mean is undefined where no root cell is defined. §on *Gastrulation E7.5* the canonical order collapses divergent terminal fates into a single terminal rank, so the reference is a branching tree flattened onto one axis. GeneTrajectory (best traj.) is scored on a per-fit subset of 5 to 8 of those markers, so it is not comparable, and having no fixed panel to resample it carries no bootstrap interval anywhere in the table. Interpretation in the text.

Dataset	Panel/metacells	$\lvert\rho\rvert$ ↑	Bootstrap mean ↑
Pancreas	500/250 (reported)	.650	–
	300/150	.828	.805
Gastrulation	500/250 (reported)	–	–
	300/150	.515	.511
Gastr. Eryth.	500/250 (reported)	.964	.945
	300/150	.272	.495
Hemogenic Endo.	500/250 (reported)	.346	.368
	700/350	.722	.701
Paul15	500/250 (reported)	.038	.394
	700/350	.064	.388

Table S12 GeneTrajectory marker recovery ($\lvert\rho\rvert$ vs the canonical marker progression) under the uniform reported configuration and one alternative per dataset. We report 500/250 on every dataset. On Pancreas, Hemogenic Endothelium and Paul15 the alternative scores higher for GeneTrajectory, so the uniform setting is not tuned in our favor. Bootstrap means for the reported configuration are those of Table S11. A dash marks an undefined quantity. On Gastrulation the 500/250 extraction assigns only 2 of the 11 canonical markers to any single trajectory, so no correlation is defined, and on Pancreas 1 of the 15 markers is unassigned, so the marker bootstrap is skipped.

chance-level standard deviation, so neither is evidence of recovery. The log1p expression baseline trails PRISM-GEP on all nine and averages .405, and the chance level exceeds it on five of the nine.

Two caveats belong with the table. The marker sets behind these scores are small, running from 4 to 20 genes, so the Spearman standard error is ≈0.3 and only large gaps are meaningful. The PRISM-GEP–contextual difference is well within that noise, whereas the chance level and static scGPT's Hemogenic collapse are not. The second caveat concerns the log1p column as a whole. On six of the nine datasets the gene–gene cosine graph is disconnected, the diffusion eigenvalue 1 is degenerate, and the ordering eigenvector is an arbitrary basis choice inside that eigenspace, so a 10^{-7} perturbation of the input moves the value across a wide interval. Under that perturbation the log1p value spans $[.009, .821]$ on Gastrulation, $[.519, .964]$ on Gastrulation Erythroid, $[.183, .775]$ on Hemogenic Endothelium, $[.013, .600]$ on Paul15, $[.051, .975]$ on Dentate Gyrus, and $[.378, .945]$ on Endoderm, around the point values printed in main-text Table 1a. We report the values the shipped pipeline returns and make no claim that they are invalid, but a reader rerunning this baseline on different hardware can legitimately obtain a different log1p row, and on several of those cells the attainable range reaches above PRISM-GEP.[3]

We therefore draw no ranking between PRISM-GEP and the contextual pass. PRISM-GEP holds the better mean and median, the contextual pass holds the better mean rank, and the honest reading is that a representation derived from the dataset itself reaches foundation-model accuracy here without foundation-model pretraining. Marker-bootstrap 95% CIs for the PRISM-GEP ordering are in Table S11.

S7.4 Gene-trajectory cascade heatmaps

A correct gene order should paint a diagonal when the cells are laid out along a developmental axis the method never saw. Figure S6 tests exactly that on four lineages, and PRISM-GEP produces a cascade on all four. Cells are ordered by an *independent* Slingshot pseudotime that PRISM-GEP never sees, and genes by each method's recovered trajectory. Each panel shows, left to right, the canonical marker order (ground truth), PRISM-GEP Step-(ii) (gene-topic Hellinger diffusion), GeneTrajectory's best-matching extracted trajectory, GeneTrajectory's leading-eigenvector ordering, and the root-supervised DPT-weighted-mean baseline. The top strip encodes lineage stage, and $\lvert\rho\rvert$ is the Spearman correlation of each gene order against the canonical order. These panels show a single model fit rather than an average, because a heatmap displays one specific recovered gene order and orderings from different fits cannot be averaged into one. The $\lvert\rho\rvert$ values quoted below and printed on the panels are therefore single-fit values, and they differ slightly from the ten-fit means in Table S11.

PRISM-GEP is not the strongest method on every panel. On Pancreas it yields a clean cascade ($\lvert\rho\rvert$=0.74) comparable to GeneTrajectory's eigenvector ordering (0.78), the root-supervised DPT baseline, given an author-chosen root cell, is strongest here (0.91), and GeneTrajectory's native best trajectory covers only 9 of the 15 markers (0.37). On the harder multi-lineage gastrulation atlas PRISM-GEP recovers a coherent cascade (0.80) and the DPT baseline matches it, while GeneTrajectory's leading-eigenvector ordering is strongest (0.92). GeneTrajectory's native best trajectory is *undefined* there, because its optimal-transport extraction assigns only 2 of the 11 cross-lineage canonical markers to any single trajectory and the rest to "Other", which is exactly why we score GeneTrajectory three ways in Table S11. On the erythroid lineage PRISM-GEP again recovers a clean cascade against the independent Slingshot cell axis. Bonemarrow haematopoiesis completes the set, a further developmental lineage checked against the same independent cell axis. There PRISM-GEP recovers the full twenty-marker cascade at 0.91, the DPT baseline is marginally ahead (0.92), GeneTrajectory's eigenvector ordering reaches 0.82, and its native best trajectory covers only 11 of the 20 markers (0.58).

S7.5 Qualitative gene-ordering illustrations

These figures illustrate the Step-(ii) ordering on two datasets that lack a published ordinal ground truth, where no rank correlation can be scored. Each figure takes a single GEP from one PRISM-GEP run and shows its within-program gene ordering, which also demonstrates gene-level mixed membership (a gene recurring across two programs). These are qualitative examples, not a statistical claim. The quantitative validation of the same ordering is the gene-trajectory benchmark in the main text.

On *BreastCancer* both recovered trajectories run along canonical tumor–microenvironment axes (Fig. S7). The first goes from HER2–luminal epithelium through hypoxia and ECM remodeling to immune–stromal infiltration [Curtis et al., 2012, Semenza, 2012]. The second goes from cycling epithelium through vascular remodeling to complement and antigen-presentation modules [Winkler et al., 2020,

[3] Four cells across the two deterministic rows are corrected here after they proved unreproducible from the code paths that are supposed to produce them. On log1p, Gastrulation moves from .963 to .160, because the published float belonged to an unpublished PPMI variant and is not attainable by the log1p path, and Erythroid from .519 to .618, because the published value was the minimum of its own attainable set. On static scGPT, Hemogenic Endothelium moves from .075 to .064, having been scored against a marker order we have since corrected, and Pancreas from .811 to .831, which is what every current code path returns. Two of the four favor PRISM-GEP and two do not.

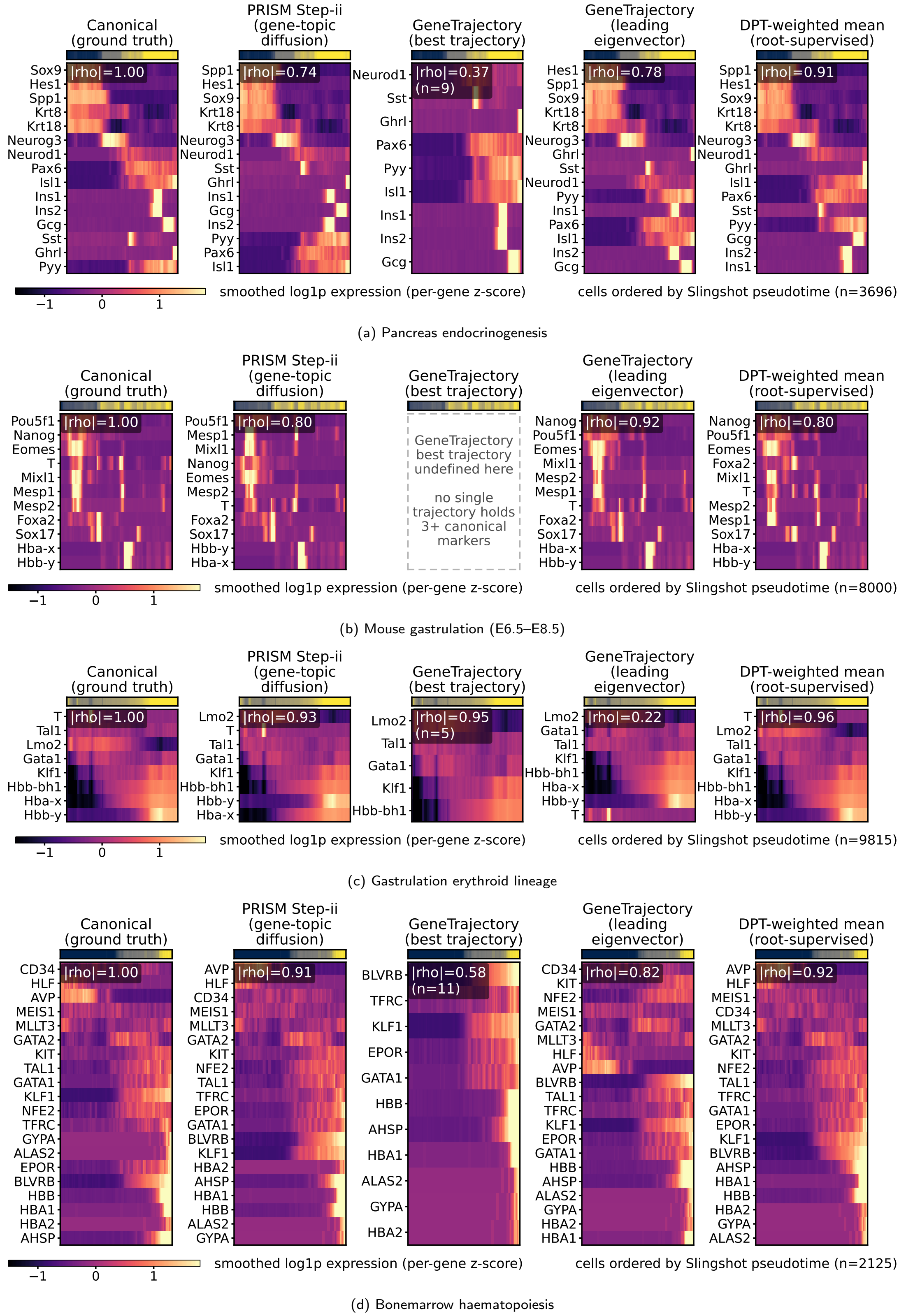

Figure S6 Gene-trajectory cascade heatmaps. Doubly-ordered gene×cell heatmaps in GeneTrajectory's Extended-Data idiom: cells ordered by an *independent* Slingshot pseudotime, genes by each method's recovered order, so a clean diagonal cascade is good. Panels and $|\rho|$ values are described in the text.

Alt text: Panels (a) to (d), for pancreas, gastrulation, gastrulation erythroid and bonemarrow, each a row of five gene-by-cell heatmaps in a dark-purple-through-orange-to-pale-yellow colormap: Canonical ground truth, PRISM Step-ii, GeneTrajectory best trajectory, GeneTrajectory leading eigenvector and DPT-weighted mean. Gene names label the rows, cells run left to right by pseudotime under a colored strip, and an absolute rho is printed in each panel. Bright bands run diagonally in most panels. In (b) the best-trajectory panel is an empty dashed box reading undefined here.

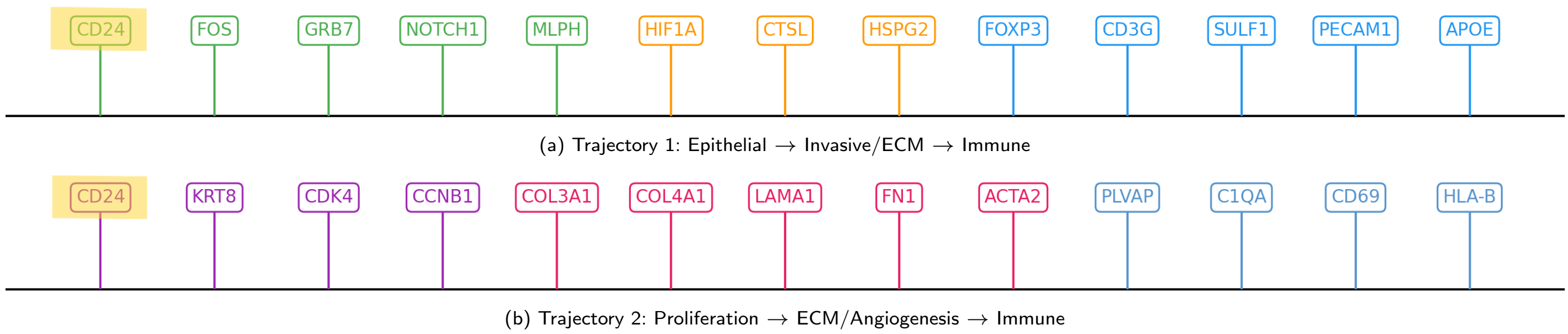

Figure S7 Gene ordering in *BreastCancer* inferred by PRISM-GEP. In each panel color encodes phase along the axis, from early to late: epithelial and cycling, then hypoxia, invasion and ECM remodeling, then immune and stromal infiltration. The two panels use different palettes. CD24 recurs across both trajectories, exemplifying gene-level mixed membership.

Alt text: (a) A horizontal line with 13 gene names on stalks: CD24, FOS, GRB7, NOTCH1 and MLPH in green, HIF1A, CTSL and HSPG2 in orange, then FOXP3, CD3G, SULF1, PECAM1 and APOE in blue. (b) The same layout: CD24, KRT8, CDK4 and CCNB1 in purple, COL3A1, COL4A1, LAMA1, FN1 and ACTA2 in red, then PLVAP, C1QA, CD69 and HLA-B in blue. CD24 has a yellow box at the start of both panels.

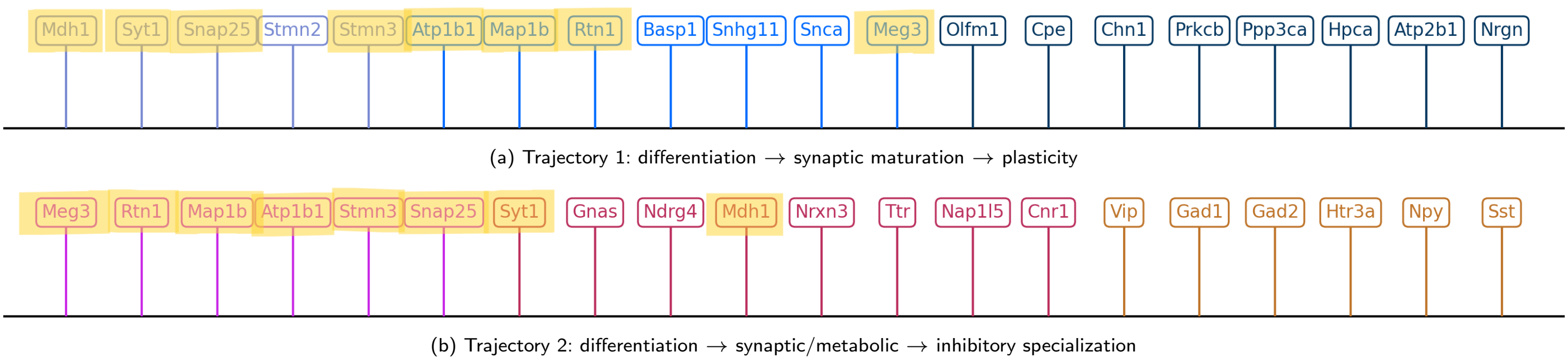

Figure S8 Gene ordering in *Zeisel_brain* inferred by PRISM-GEP. Yellow marks a shared neuronal-differentiation/early-synaptic core. Meg3 and Mdh1 recur across both trajectories, exemplifying module reuse.

Alt text: (a) A horizontal line with 20 gene names on stalks, Mdh1 to Nrgn, labeled in light to dark blue. (b) The same layout with 20 genes, Meg3 to Sst, labeled in magenta, red and orange. Eight genes carry yellow boxes in each panel, Mdh1, Syt1, Snap25, Stmn3, Atp1b1, Map1b, Rtn1 and Meg3, seven of them within the first eight positions.

Roumenina et al., 2019]. The marker genes for each phase are printed on the figure. CD24 recurs across both, which is gene-level mixed membership.

A similar pattern holds in *Zeisel_brain* (Fig. S8). The first trajectory goes from neurite-outgrowth and cytoskeletal genes through core presynaptic machinery to Ca^{2+}-dependent plasticity genes [Lewis et al., 2013, Südhof, 2013, Malenka and Bear, 2004]. The second shares that core but ends in an inhibitory, neuromodulatory state consistent with GABAergic and Vip/Lamp5-like interneuron programs [Tasic et al., 2018]. The marker genes for each phase are printed on the figure. Meg3 and Mdh1 recur across both, reflecting pleiotropy and reuse of synaptic machinery rather than a model limitation [Zhang et al., 2022].

S7.6 Native trajectory and simulation panels

Figure S9 shows PRISM-GEP's own within-program gene trajectories for five representative datasets, each panel ordering a program's genes by the Hellinger diffusion coordinate from early to late. Figures S10–S11b then place PRISM-GEP into GeneTrajectory's own display idioms and simulation model, with Figure S11b reproducing GeneTrajectory's Extended Data Fig. 1 on its own synthetic single-cell simulators (cell-cycle ring, branching tree, line+ring, tree+ring). On GeneTrajectory's synthetic simulators, where a ground-truth gene order exists, PRISM-GEP reconstructs the activation cascade from expression alone with rank agreement $|\rho|$ of 0.95 (ring), 0.98 (tree, within-lineage), 0.81 (line+ring) and 0.87 (tree+ring, within-lineage). On branching processes, a single global diffusion axis cannot linearise several lineages at once, so we grade and display the ordering *within* each lineage.

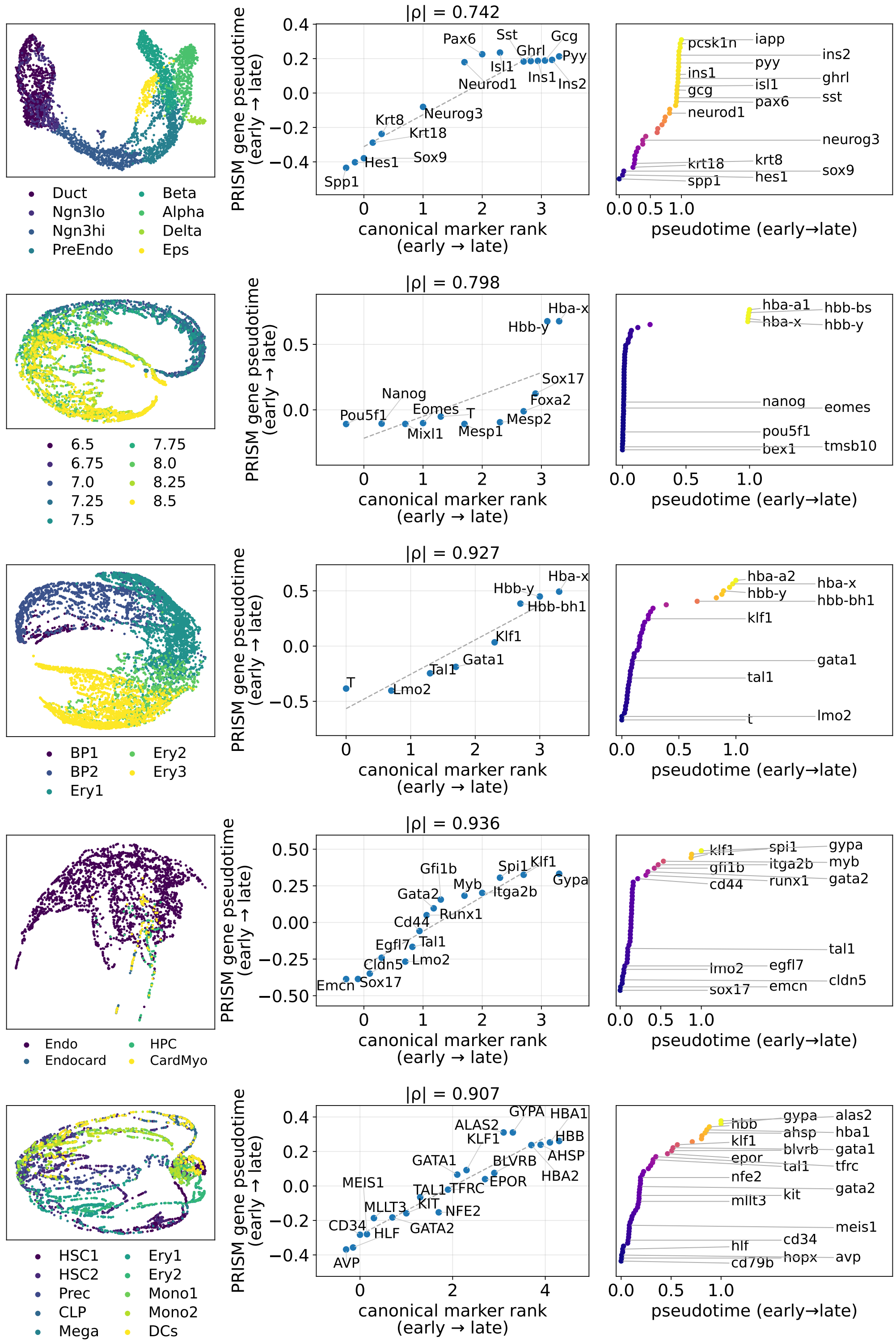

Figure S9 PRISM-GEP's own recovered gene trajectories: Pancreas, Gastrulation, Gastrulation Erythroid, Hemogenic Endothelium and Bonemarrow (native Step-(ii) ordering, one dataset block per row, top to bottom). Each panel orders a program's genes by the Hellinger diffusion coordinate, early to late. Within each dataset block the three panels show, left to right, the PRISM cell embedding colored by published cell type, marker recovery of PRISM's recovered order of the canonical markers against their canonical rank (the annotated $|\rho|$ is the absolute Spearman correlation, chance about 0.31), and the program genes in that same Hellinger-diffusion order.

Alt text: Five rows, one per dataset: Pancreas, Gastrulation, Gastrulation Erythroid, Hemogenic Endothelium and Bonemarrow, each with three panels. Left, a scatter of cells colored by cell type, or by embryonic stage in the Gastrulation row, with a legend below. Middle, a scatter of PRISM gene pseudotime against canonical marker rank with labeled points, a dashed diagonal, and the absolute rho printed above: 0.742, 0.798, 0.927, 0.936 and 0.907. Right, program genes as colored dots along a pseudotime axis from 0 to 1 with a subset of lower-case gene names on leader lines.

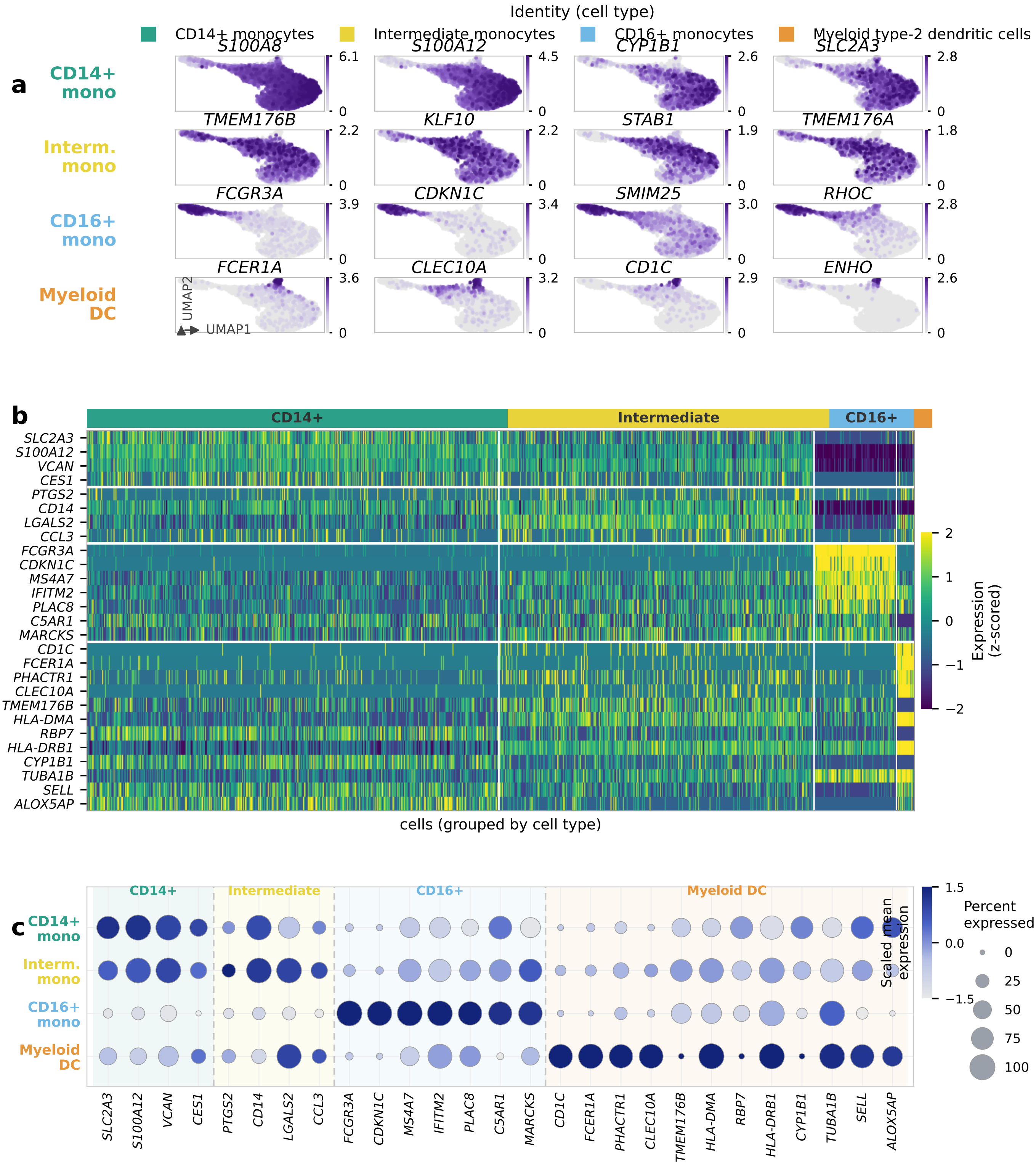

Figure S10 Myeloid marker panels in the GeneTrajectory Extended-Data-Figure-2 idiom: panel (b) a gene$\times$cell z-scored expression heatmap with cells grouped by identity, panel (c) a Seurat-style marker dot plot. Read together, the two panels show that the myeloid marker genes PRISM-GEP orders are expressed specifically in their matching cell populations, a qualitative check that the recovered program tracks a real myeloid identity rather than a diffuse signal.

Alt text: (a) A four-by-four grid of small UMAP plots, rows CD14+ mono, Interm. mono, CD16+ mono and Myeloid DC, each shaded in purple by one gene's expression with its own color bar. (b) A gene-by-cell heatmap of z-scored expression from -2 to 2 with 27 gene rows and cells grouped under header bars for CD14+, Intermediate and CD16+, plus a narrow orange bar for dendritic cells. (c) A dot plot of the same genes by the four cell types, dot size for percent expressed and blue intensity for scaled mean expression.

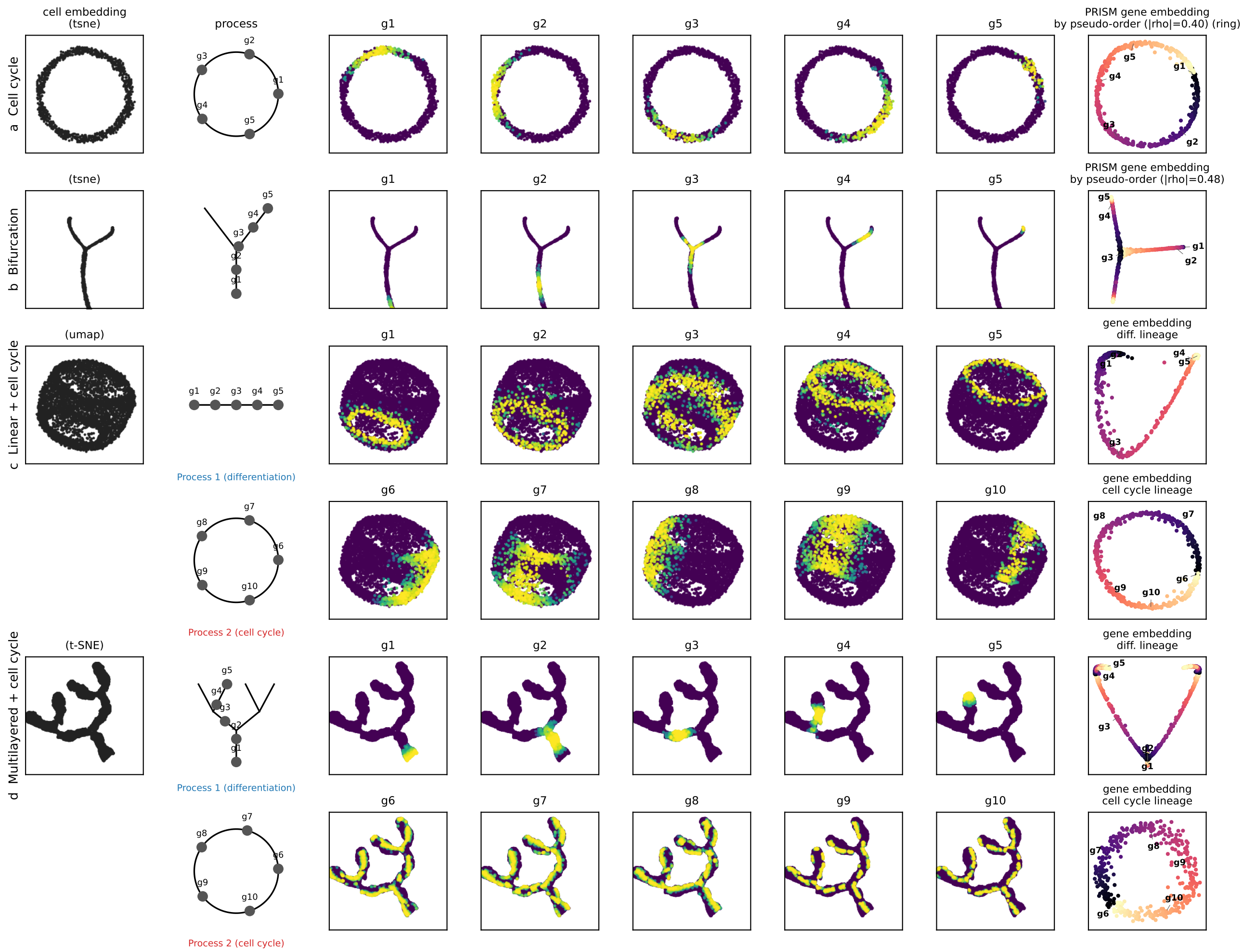

(a) Simulation benchmark (GeneTrajectory Fig. 2 idiom)

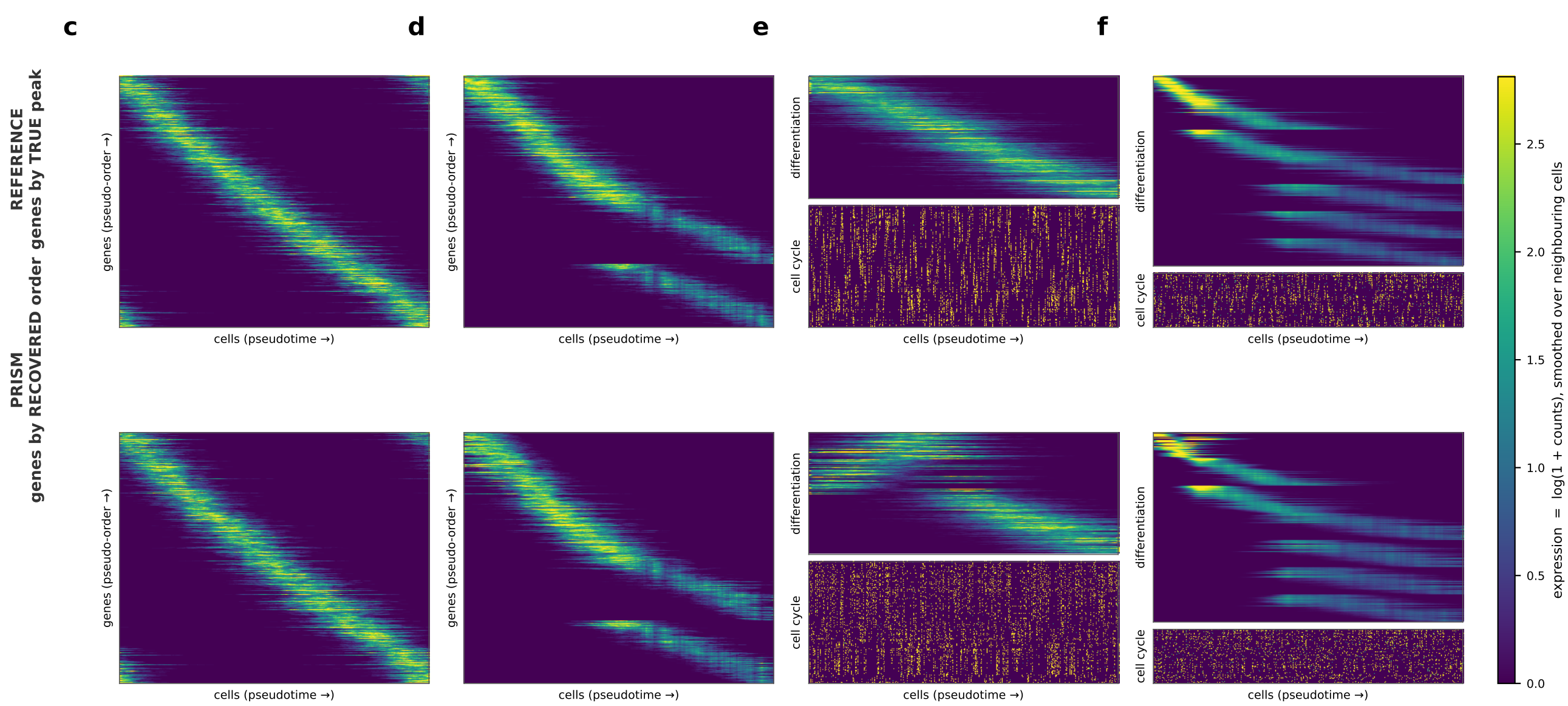

(b) Activation cascades (Extended-Data-Fig. 1 idiom)

Figure S11 Synthetic-simulator validation. (a) PRISM-GEP analogue of GeneTrajectory's Figure 2 on its own simulators. Columns: cell embedding, process schematic, gene expression, and PRISM-GEP's gene embedding colored by the recovered pseudo-order (rows c,d carry differentiation and cell cycle over one embedding, and the final column separates them). **(b)** Activation cascades: the top row orders genes by the ground-truth activation peak, the bottom row by PRISM-GEP's *recovered* order with no access to it, so a surviving diagonal is good. Per-simulator rank agreement ($|\rho| \in [0.81, 0.98]$) is in the text.

Alt text: (a) Rows for four simulations: cell cycle, bifurcation, linear plus cell cycle, and multilayered plus cell cycle. Each shows a black cell embedding, a process schematic with numbered gene groups, five copies of the embedding colored by each group's expression, and PRISM's gene embedding colored by pseudo-order. The last two rows have two processes and so ten expression plots and two embeddings. (b) Two rows of gene-by-cell heatmaps, REFERENCE above PRISM, for columns c to f, each with a diagonal band, splitting into branches in d and stacked above a speckled cell-cycle heatmap in e and f.

S8 Cell ordering, a by-product

Cell ordering is a proof of concept rather than a benchmarked contribution of this paper, so we report it here in full. Main-text Table 2 compares the label-free PRISM-GEP ordering against the dedicated cell-trajectory methods across the same nine datasets used for gene-trajectory recovery, namely those carrying a canonical marker order. We fix that roster in advance rather than choosing it here, so the datasets are not selected to suit the result. It also excludes by rule, rather than by score, the datasets for which no developmental axis exists and an ordering would have to be invented.

PRISM-GEP is on par with the dedicated methods, which is the honest summary. It holds the best mean rank of the four methods. Setting aside the two † columns that cannot separate methods, it is best on three of the remaining seven datasets and worst on one, and its mean over the nine (0.722) sits between DPT (0.727) and Slingshot (0.710). Slingshot is clearly better on Dentate Gyrus (0.840 against 0.571), and we report that as it stands. Two qualifications belong with the table rather than in a footnote. First, *Hemogenic Endothelium* and *Endoderm* have only two label ranks, and with two ranks an ordering and its exact reversal score identically, so those two columns cannot separate any method from any other. They are marked † and should not be read as wins. Second, we include the first principal component of the expression matrix as a trivial floor, because on several datasets it lands within 0.05 of the best method. A cell-ordering correlation is only interpretable next to that floor.

The cell-ordering metric that feeds Step-(ii) is itself a swappable component, so we ask whether the primary conclusion depends on that choice. Table S13 varies the label-free cell-ordering rule applied to the same PRISM-GEP GEP-attribution vectors and reports |Spearman ρ| against the published ordinal lineage (K_{topics}=#types). Every variant and every hyper-parameter (k=15 neighbours, diffusion n_{evec}=15, shrinkage λ, principal-curve knots) is fixed *a priori* rather than tuned to the score, and we report all of them, including the metrics that did not help. Starting from the Jensen–Shannon geometry, the diffusion-map coordinate (used in main-text Table 2) gives the best mean and the Hellinger metric is the most consistent, while the global- and local-Mahalanobis metrics do *not* improve over plain JS-PHATE. The local anisotropic metric is unstable on the multi-lineage Gastrulation atlas at K_{topics}=9, where the per-cell covariance is under-determined ($\rho = 0.41 \pm 0.13$). We keep the Jensen–Shannon default for simplicity and broad applicability, and the Euclidean PHATE-1 row is the plain Euclidean baseline ordering. This ablation varies only the ordering rule, holding the decomposition fixed at K_{topics}=#published types on three datasets, whereas main-text Table 2 compares against the dedicated methods at K_{topics}=5 across nine. The two are therefore read separately: this table asks which ordering rule is best, and main-text Table 2 asks how the chosen rule compares against dedicated cell-trajectory methods. The best PRISM-GEP variant mean stays close to those dedicated methods, consistent with the robustness the method targets. We make no superiority claim.

Table S13 Cell-ordering ablation. Label-free cell-ordering rules applied to the same PRISM-GEP GEP-attribution vectors, |Spearman ρ| ↑ vs the published ordinal lineage (mean ± s.d. over 10 seeds, K_{topics}=#types). Best PRISM-GEP variant mean in **bold**. Interpretation and hyper-parameters are in the text.

Cell-ordering rule	Pancreas	Gast. Eryth.	Gastrulation	Mean
PHATE-1, Euclidean *(baseline)*	.830±.017	.778±.034	.515±.047	.708
PHATE-1, Jensen–Shannon *(reference)*	.871±.008	.813±.014	.617±.025	.767
+ principal curve	.866±.015	.808±.007	.509±.065	.727
+ kNN denoise	.832±.012	.782±.008	.618±.035	.744
Diffusion-map DC1, Jensen–Shannon	.942±.004	.807±.006	.589±.036	**.779**
Diffusion pseudotime, Jensen–Shannon	.929±.006	.808±.008	.548±.015	.762
PHATE-1, Hellinger ($\sqrt{}$-simplex)	.880±.013	.817±.009	.618±.027	.772
Diffusion-map DC1, global Mahalanobis	.911±.011	.793±.012	.539±.025	.747
Diffusion-map DC1, local Mahalanobis	.883±.042	.798±.012	.408±.130	.697

S9 Hyperparameter search

S9.1 Sensitivity to the neighborhood size k_{nn} and the topic count K_{topics}

We swept the Stage-A neighborhood size $k_{\mathrm{nn}} \in \{5, 10, 15, 20, 30, 50\}$ on nine datasets described in the main text, recomputing the $\boldsymbol{\beta}$ prior and retraining MALLET at every k_{nn}. Figure S12 shows the metric-vs-k_{nn} curves for the eight enrichment-bearing datasets. Across datasets the metric span is small: Coherence ≤ 0.054 absolute, Coverage ≤ 0.05, and Strength at most 15.1% of the per-dataset mean (Pancreas, with all other datasets $\leq$ 9.3%), all within seed-to-seed noise. The default $k_{\mathrm{nn}} = 15$ (the standard `scanpy`/Seurat convention) lies inside the small-spread region on every dataset. BreastCancer is omitted from the figure because it is unlabeled and yields no significant GO-BP enrichment, so its Coverage and Strength are undefined.

The default K_{topics}=5 also survives a sweep of the topic count. We retrain PRISM-GEP over ten seeds at $K_{\mathrm{topics}} \in \{3, 5, 8, 10\}$ and at each dataset's published-cell-type count, and Table S14 reports every setting. Three datasets depart from that grid. PBMC3k and Zeisel are small, so they are reported on a reduced two-point grid, and Gastrulation E7.5, whose 27 published types lie beyond the swept range, instead gets K_{topics}=15 as an intermediate point. The gene-set metric means vary smoothly with K_{topics} and none degenerates. The std = 0 entries at $K_{\mathrm{topics}} = 3$ reflect cross-seed convergence, not a degenerate metric. The default $K_{\mathrm{topics}} = 5$ sits in the stable region and setting K_{topics} = #types yields no systematic gain, confirming $K_{\mathrm{topics}} = 5$ as a conservative default. Table S14 reports two Strength bases: Strength$_{\mathrm{peak}}$ is the single-best sharpness $-\log_{10}(q_{\min})$, and Strength$_{\mathrm{mean}}$ is the mean $-\log_{10} q$ over significant pathways, which is the basis used in Table S7. This sweep is a separate set of fits at a different optimize-interval setting, so its K_{topics}=5 entries are not expected to match that table and differ from it by as much as 1.5 on Hemogenic Endothelium. It should be read for the trend across K_{topics} rather than against the primary numbers.

A complementary held-out perplexity sweep (85/15 cell split, sklearn LDA, Figure S13) reaches its optimum at K_{topics}=5 on three of six datasets and within ∼3% of the optimum on two more. Only *Zeisel_brain* prefers a larger K_{topics} (∼15). This model-fit criterion is orthogonal to the gene-set metrics, and the two agree that K_{topics}=5 is a sound default.

S10 Cell-level views of the recovered programs

S10.1 Cell embeddings in program space

Clustering cells is not what PRISM-GEP is built to do. It is a soft decomposition, so every cell carries a mixture over programs rather than a label, and a dedicated hard-clustering method is the better

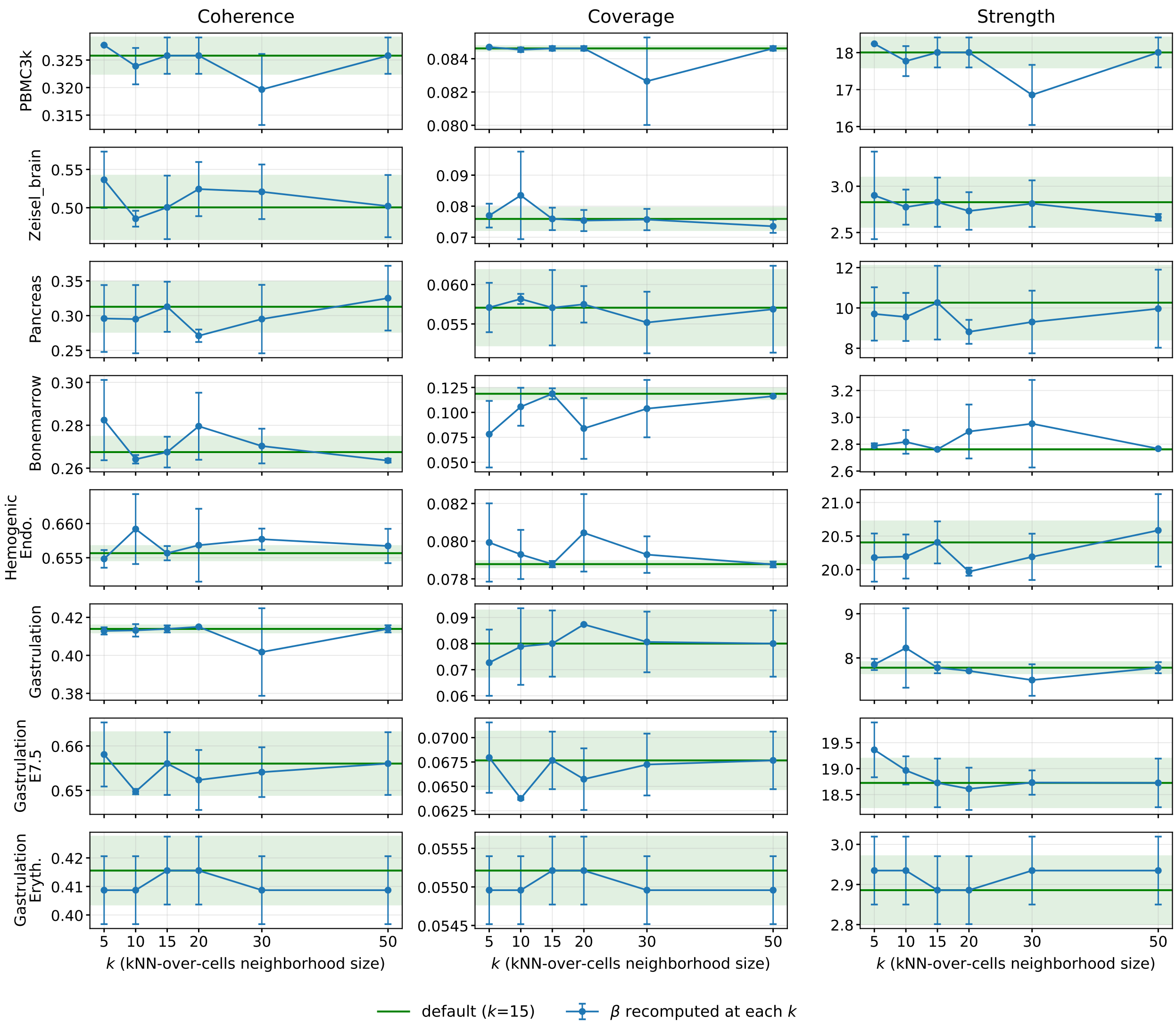

Figure S12 Sensitivity of Coherence, Coverage and Strength to the neighborhood size k_{nn} across the eight datasets with significant GO-BP enrichment. Blue traces recompute $\boldsymbol{\beta}$ and retrain MALLET at each k_{nn}. The green line and band mark the default $k_{\mathrm{nn}}=15$ baseline $\pm$ one std, so a good result is a curve that stays inside that band.

Alt text: A grid of 24 line plots, eight dataset rows by three metric columns, Coherence, Coverage and Strength. The x-axis is the neighborhood size k at 5, 10, 15, 20, 30 and 50. A blue line with error bars traces the metric, and a green line with a light-green band marks the default k=15 value plus or minus one standard deviation. Most blue traces stay inside or near the band, with a few points outside it.

tool for that job (Table S9). We include this gallery as a proof of concept for one narrow question: does the K_{topics}-dimensional bottleneck throw the cell-type structure away? Each cell's GEP-attribution vector (MALLET's document–topic distribution) is embedded with UMAP and shown against a raw-expression UMAP reference, with cells colored by published cell-type labels (BreastCancer has no label file, so it is colored by dominant GEP). Where the two agree, the bottleneck preserved cell-type identity. As a concrete example, in PBMC3k the two monocyte subtypes (CD14+ and FCGR3A+) co-localize because they share a single dominant myeloid program (GEP 3 in 100% of cells of each subtype, centroid distance 0.045 in the attribution simplex, the smallest of any cell-type pair). That is expected biology for a $K_{\mathrm{topics}}=5$ decomposition, not an embedding artifact.

S10.2 Per-program heatmaps

For each GEP we provide a per-GEP top-gene×cell heatmap, with genes ordered by the Step-(ii) pseudotime and cells ordered by GEP attribution. We show it for three datasets: the two datasets of the qualitative trajectory illustrations (*BreastCancer* in Figure S7 and *Zeisel_brain* in Figure S8), together with *PBMC3k*. The three attribution heatmaps share Figure S15, two datasets per page for legibility. The same heatmap for every remaining dataset is included in the public repository.

A well-formed program appears as a coherent block. Each GEP places most of its probability mass on a small, distinct set of genes, and those genes are co-expressed specifically in the cells most strongly attributed to that program, which is what produces the block. A program without a clear cell block would instead indicate a redundant, poorly separated topic. This concentration is the qualitative

Dataset	K_{topics}	Coherence	Coverage	Strength$_{peak}$	Strength$_{mean}$
Pancreas	3	0.300 (0.000)	0.820 (0.045)	12.490 (0.227)	6.212 (0.429)
	**5**	0.312 (0.040)	0.777 (0.025)	10.031 (1.443)	4.515 (0.503)
	8†	0.284 (0.015)	0.731 (0.036)	9.325 (0.440)	4.528 (0.316)
	10	0.274 (0.018)	0.721 (0.024)	9.394 (0.316)	5.002 (0.438)
Bonemarrow	3	0.296 (0.001)	0.729 (0.000)	3.769 (0.000)	1.984 (0.000)
	**5**	0.270 (0.007)	0.439 (0.083)	2.868 (0.169)	2.002 (0.149)
	8	0.288 (0.002)	0.579 (0.089)	3.500 (0.513)	1.987 (0.071)
	10†	0.292 (0.007)	0.562 (0.036)	3.530 (0.332)	2.081 (0.175)
Hemogenic Endo.	3	0.659 (0.000)	0.978 (0.000)	19.901 (0.000)	7.417 (0.000)
	4†	0.664 (0.008)	0.971 (0.010)	20.467 (0.673)	7.770 (0.210)
	**5**	0.657 (0.003)	0.973 (0.004)	20.574 (0.288)	7.808 (0.122)
	8	0.602 (0.024)	0.923 (0.025)	16.681 (1.867)	6.158 (0.587)
	10	0.561 (0.021)	0.914 (0.020)	13.946 (1.617)	5.084 (0.507)
Gastrulation	3	0.399 (0.003)	0.768 (0.011)	6.885 (0.226)	3.865 (0.157)
	**5**	0.396 (0.019)	0.797 (0.063)	7.677 (0.772)	3.183 (0.214)
	8	0.380 (0.011)	0.847 (0.040)	8.016 (0.780)	3.385 (0.220)
	9†	0.367 (0.009)	0.812 (0.037)	7.280 (0.800)	3.080 (0.178)
	10	0.367 (0.011)	0.802 (0.029)	7.212 (0.718)	3.272 (0.289)
Gastrulation E7.5	3	0.613 (0.000)	0.875 (0.000)	18.284 (0.000)	5.112 (0.000)
	**5**	0.656 (0.006)	0.882 (0.004)	18.858 (0.177)	5.617 (0.251)
	8	0.634 (0.005)	0.865 (0.015)	17.639 (0.352)	5.377 (0.446)
	10	0.617 (0.007)	0.877 (0.014)	15.471 (0.342)	4.088 (0.188)
	15	0.596 (0.013)	0.866 (0.023)	14.415 (0.874)	3.924 (0.352)
Gastrulation Eryth.	3	0.428 (0.000)	0.806 (0.000)	2.317 (0.000)	1.733 (0.000)
	**5**	0.412 (0.011)	0.846 (0.008)	2.911 (0.079)	1.794 (0.015)
	7†	0.404 (0.007)	0.787 (0.032)	3.005 (0.146)	1.808 (0.041)
	8	0.395 (0.012)	0.783 (0.054)	3.047 (0.275)	1.835 (0.050)
	10	0.397 (0.008)	0.777 (0.024)	3.155 (0.353)	1.833 (0.051)
PBMC3k	3	0.308 (0.006)	0.939 (0.020)	16.791 (0.904)	6.644 (0.062)
	**5**	0.323 (0.010)	0.922 (0.016)	12.061 (0.508)	4.128 (0.260)
	8†	0.326 (0.006)	0.928 (0.008)	14.290 (0.448)	5.327 (0.211)
	10	0.328 (0.006)	0.919 (0.011)	13.106 (0.398)	4.795 (0.198)
Zeisel_brain	3	0.513 (0.018)	0.734 (0.034)	2.872 (0.409)	1.864 (0.135)
	**5**	0.520 (0.038)	0.689 (0.050)	2.683 (0.145)	1.905 (0.031)
	7†	0.519 (0.032)	0.654 (0.042)	2.801 (0.295)	1.962 (0.093)
	8	0.524 (0.042)	0.672 (0.053)	3.089 (0.440)	1.984 (0.081)
	10	0.519 (0.028)	0.643 (0.061)	3.283 (0.434)	1.986 (0.071)
BreastCancer	3	0.237 (0.003)	–	–	–
	**5**	0.268 (0.001)	–	–	–
	8	0.250 (0.003)	0.438 (0.000)	1.398 (0.000)	1.398 (0.000)
	10	0.242 (0.002)	0.423 (0.032)	1.439 (0.112)	1.411 (0.027)

Table S14 Topic-count (K_{topics}) sensitivity of PRISM-GEP (mean (std) over 10 seeds). The default K_{topics}=5 is in **bold**, and † marks the sweep point equal to the dataset's number of published cell types. Gastrulation E7.5 has 27 published types, beyond the swept range, so no row is marked there. These runs enable MALLET's hyperparameter re-estimation (optimize-interval 10) rather than the optimize-interval 0 of the primary tables, the one exception being the K_{topics}=5 rows of PBMC3k, Zeisel_brain and BreastCancer, which come from earlier optimize-interval 0 runs. A good result is a metric that varies smoothly and does not collapse across K_{topics}. BreastCancer is unlabeled, so only its Coherence is defined (Coverage/Strength shown as "–"). Reproduce from `k_topics_sensitivity_agg.csv`.

counterpart of the quantitative scores, because the block-diagonal cell attribution is why the top-gene sets recover known, pathway-enriched programs (the Coverage and Strength columns of Table S7).

Two features of these panels look like defects and are not. The gene row-labels are colored by the canonical cell type or program each gene marks, with the legend below the panels, and a gene that marks no canonical program, such as a ribosomal, mitochondrial or generic housekeeping gene, is drawn in black. We do not filter those genes out, because PRISM-GEP trains on the full HVG-selected vocabulary. In the datasets that contain many of them, one or two GEPs become ribosomal/housekeeping topics that absorb these ubiquitous high-count genes and thereby keep the remaining programs cleaner, so a mostly-black program is one of these sinks rather than a failure of the method. A program whose top genes share one color and concentrate in one cell block is therefore a directly readable check that the recovered gene ordering corresponds to a known cell type. The second feature is that some genes appear in the top set of more than one GEP. This is not redundancy but a direct consequence of the mixed-membership model, in which a gene can contribute to several programs at once, something a hard one-gene-one-cluster assignment cannot represent.

The recovered blocks span recognizable programs. In *BreastCancer*, they are an epithelial/tumor program (*KRT19*, *KRT18*, *TFF1*, *ERBB2*), a plasma/B-cell program (*IGHG1*, *IGKC*) and an immune/stromal program (*APOE*, *C1QA*, *FOXP3*). In *Zeisel_brain*, they are oligodendrocyte (*Plp1*, *Mbp*, *Mog*), astrocyte (*Apoe*, *Slc1a2*, *Aqp4*) and GABAergic interneuron (*Sst*, *Vip*, *Gad1*) programs. The two pyramidal/neuronal programs share a core of synaptic genes (*Snap25*, *Syt1*, *Meg3*). In *PBMC3k*, GEP 1 is NK/cytotoxic (*NKG7*, *GNLY*, *GZMA*), GEP 3 the CD14 monocyte program (*LYZ*, *S100A8*, *CST3*) and GEP 0 antigen-presenting/dendritic (*HLA-DRA*, *CD74*). The two remaining GEPs are ribosomal/housekeeping sinks.

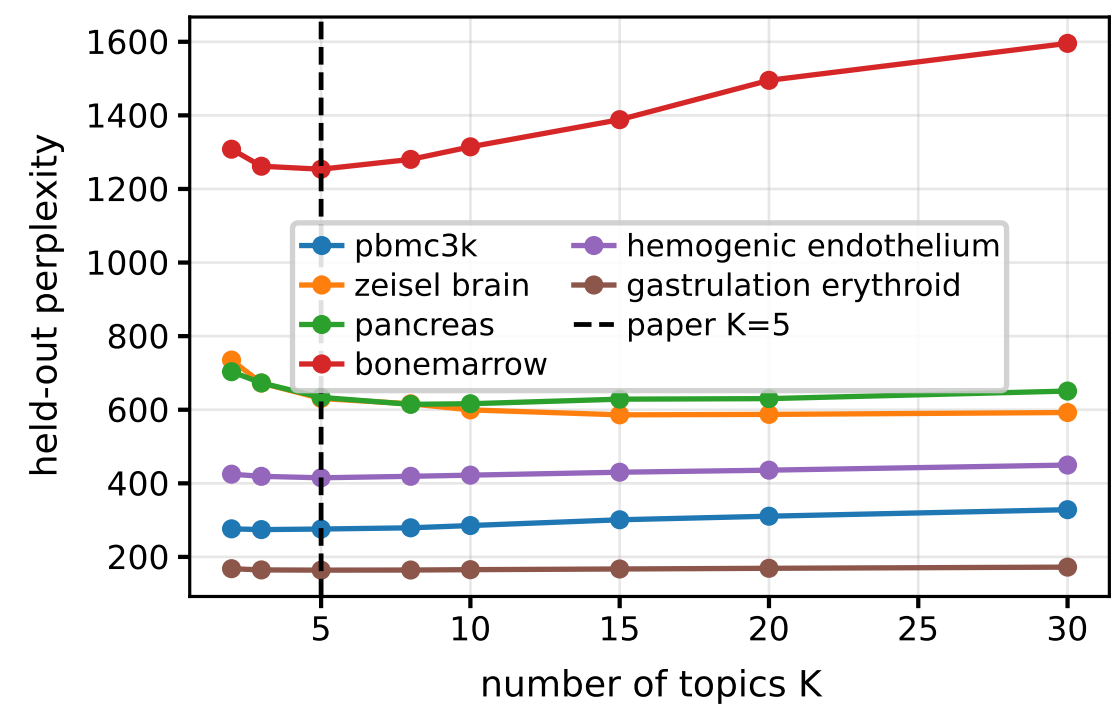

Figure S13 Held-out perplexity versus topic count K_{topics} (85/15 cell split, sklearn LDA, lower is better, dashed line marks the default K_{topics}=5), a model-fit criterion orthogonal to the gene-set metrics. A good result is a curve whose minimum sits at or near K_{topics}=5. Source: `scripts/heldout_k_sklearn.py`.

Alt text: Line chart of held-out perplexity, 200 to 1600, against the number of topics K, 2 to 30, with six colored lines for pbmc3k, zeisel brain, pancreas, bonemarrow, hemogenic endothelium and gastrulation erythroid, and a dashed vertical line at K=5. The red bonemarrow line is highest, dips slightly near K=5 and then rises steadily. Zeisel brain and pancreas start near 700, drop to about 650 by K=5 and then change only gradually. The other three lines stay below 450 and change little.

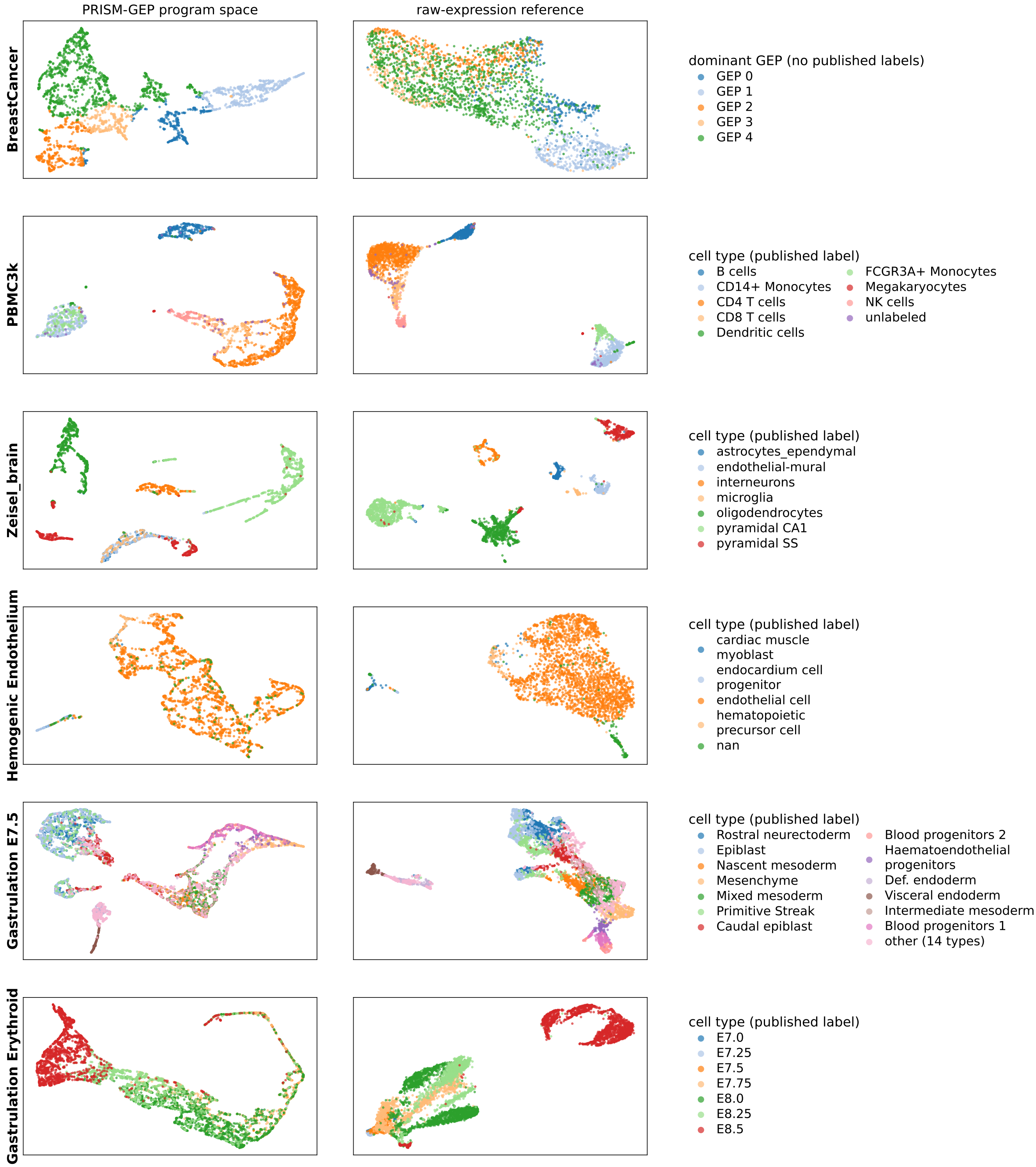

Figure S14 Cell embeddings from the PRISM-GEP program space, shown as a proof of concept. For each of the six datasets, cells are embedded by UMAP from their K-dimensional PRISM-GEP attribution vectors (left) and, for reference, from a 50-component PCA of the log1p CP10K expression (right). Points are colored by published cell-type label, except BreastCancer, which has no cell-type annotation and is instead colored by its dominant GEP. Clustering or visualizing individual cells is not what PRISM-GEP is built for. PRISM-GEP produces a soft decomposition of expression into programs, and a dedicated hard-clustering method recovers discrete cell types better than the argmax of these soft loadings. These panels are therefore included only as a proof of concept. As an honest summary we report the adjusted Rand index (ARI) between each cell's argmax PRISM-GEP program and its published label. The program space clearly recovers the cell types on PBMC3k (ARI 0.54) and Zeisel_brain (ARI 0.85), partially recovers them on Gastrulation Erythroid (ARI 0.31), and does not cleanly recover them on Gastrulation E7.5 (ARI 0.27) or Hemogenic Endothelium (ARI 0.08). BreastCancer has no labels and so is not scored.

Alt text: Six rows of two UMAP scatter plots, for BreastCancer, PBMC3k, Zeisel_brain, Hemogenic Endothelium, Gastrulation E7.5 and Gastrulation Erythroid: the PRISM-GEP program space on the left and the raw-expression reference on the right. Points are colored by the legend beside each row, dominant GEP for BreastCancer and published cell types or embryonic stages for the others. PBMC3k and Zeisel_brain show well separated clusters in both columns, while Hemogenic Endothelium is almost entirely one orange color.

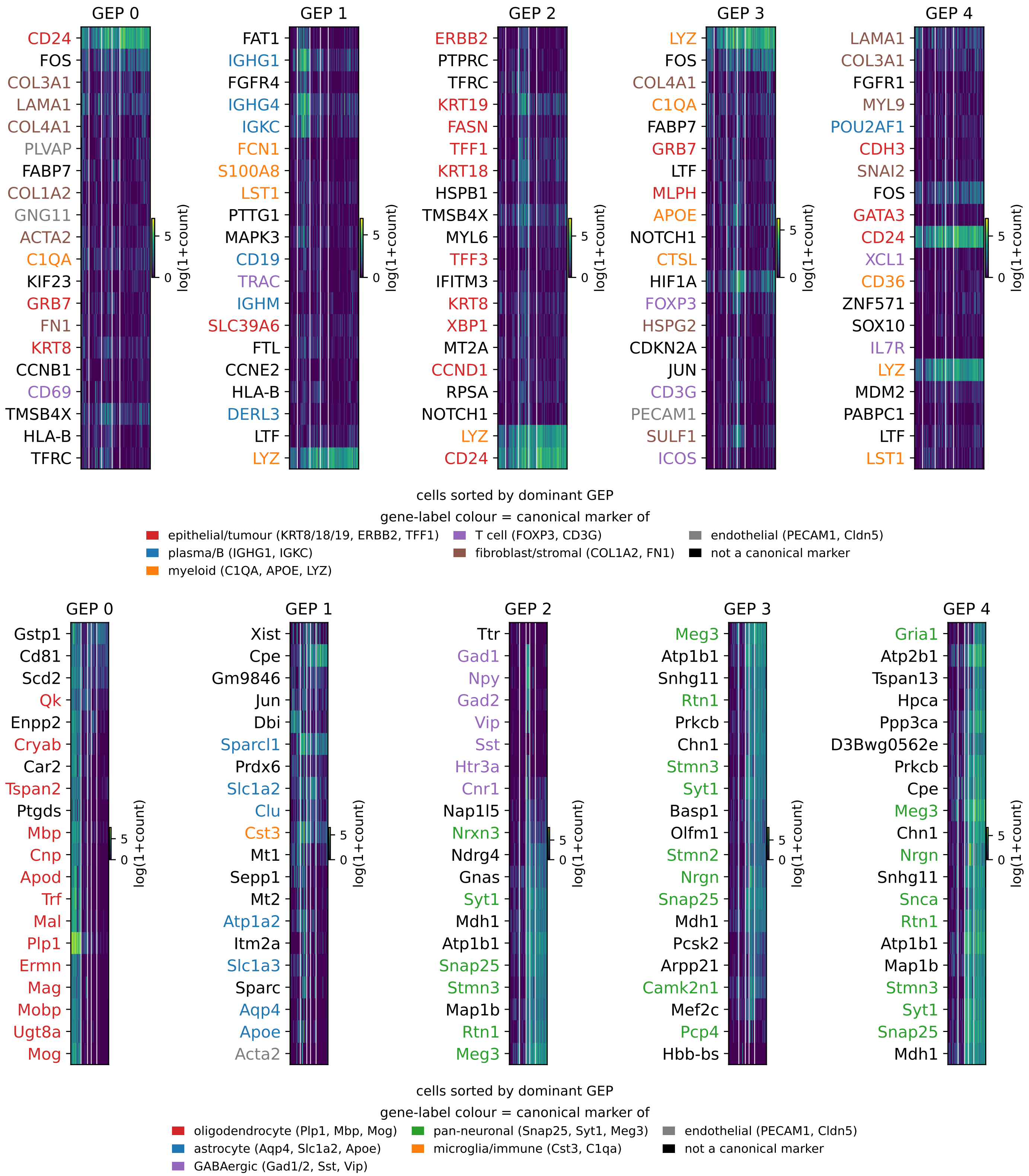

Figure S15 Per-GEP top-gene×cell attribution heatmaps for three datasets, two per page for legibility. This page: *BreastCancer* (top, the dataset of Figure S7) and *Zeisel_brain* (bottom, Figure S8). *PBMC3k* continues under the same figure number. Within each dataset, cells (columns) are sorted by dominant GEP and genes (rows) by the Step-(ii) within-program pseudotime, so a clean block per GEP is good. Gene row-labels are colored by the canonical cell type each gene marks (legend below each panel). Black labels are genes that are not canonical markers (ribosomal, mitochondrial or housekeeping), which are not filtered from the vocabulary and collect into one or two housekeeping GEPs per dataset, leaving the others cleaner. The interpretable programs are epithelial/tumor and plasma/B-cell in *BreastCancer*, oligodendrocyte, astrocyte and GABAergic in *Zeisel_brain*, and NK/cytotoxic (GEP 1) and CD14 monocyte (GEP 3) in *PBMC3k*.

Alt text: Two rows of five narrow heatmaps, one per GEP 0 to 4, for BreastCancer on top and Zeisel_brain below, each listing 20 gene names and showing cells as columns in a purple-to-yellow log(1+count) scale. Gene names are colored by canonical marker class as in the legend under each row, with black for genes that are not canonical markers. Bright blocks appear in different column ranges for different GEPs.

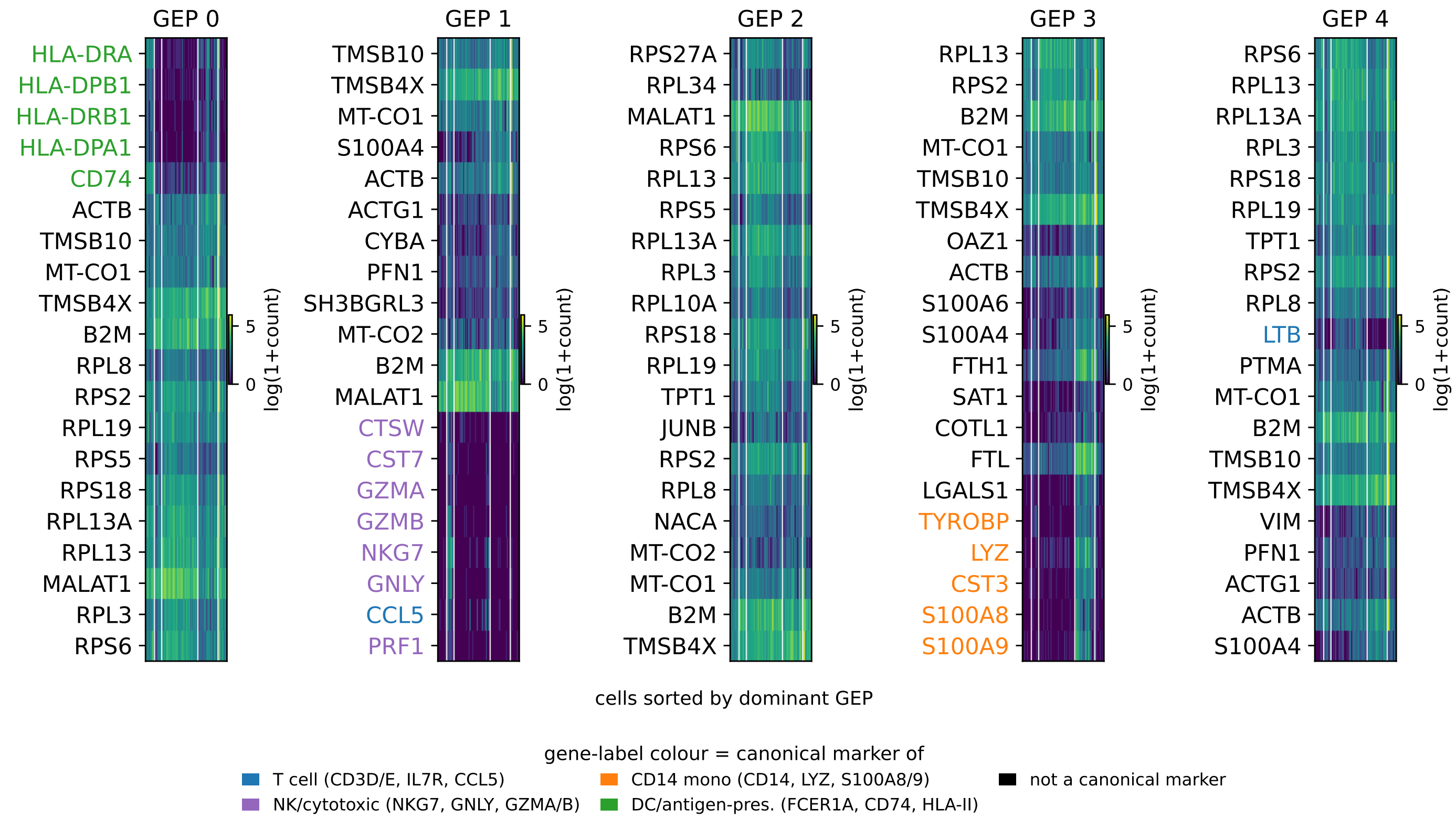

Figure S15 (Continued.) Attribution heatmap for *PBMC3k*. Axes, ordering and gene-label colors as on the first page. GEP 1 is NK/cytotoxic and GEP 3 the CD14 monocyte program, while the ribosomal/housekeeping GEPs are mostly black.

Alt text: One row of five narrow heatmaps for PBMC3k, GEP 0 to 4, each listing 20 gene names with cells as columns in a purple-to-yellow log(1+count) scale. Most names are black. GEP 0 has four HLA genes and CD74 in green at the top, GEP 1 has purple NK genes such as NKG7 and GZMB at the bottom, and GEP 3 has orange monocyte genes such as LYZ and S100A8 at the bottom.

References

Y. Baran, A. Bercovich, A. Sebe-Pedros, Y. Lubling, A. Giladi, E. Chomsky, Z. Meir, M. Hoichman, A. Lifshitz, and A. Tanay. Metacell: analysis of single-cell rna-seq data using k-nn graph partitions. *Genome Biology*, 20(1):206, 2019. doi: 10.1186/s13059-019-1812-2.

Y. Benjamini and Y. Hochberg. Controlling the false discovery rate: A practical and powerful approach to multiple testing. *Journal of the Royal Statistical Society: Series B*, 57(1):289–300, 1995.

D. M. Blei, A. Y. Ng, and M. I. Jordan. Latent dirichlet allocation. *Journal of Machine Learning Research*, 3:993–1022, 2003.

R. R. Coifman and S. Lafon. Diffusion maps. *Applied and Computational Harmonic Analysis*, 21(1):5–30, 2006.

H. Cui, C. Wang, H. Maan, K. Pang, F. Luo, N. Duan, and B. Wang. scgpt: toward building a foundation model for single-cell multi-omics using generative ai. *Nature Methods*, 21(8):1470–1480, 2024. doi: 10.1038/s41592-024-02201-0.

C. Curtis, S. P. Shah, S.-F. Chin, and et al. The genomic and transcriptomic architecture of 2,000 breast tumours. *Nature*, 486:346–352, 2012. doi: 10.1038/nature10983.

E. Dann, N. C. Henderson, S. A. Teichmann, M. D. Morgan, and J. C. Marioni. Differential abundance testing on single-cell data using k-nearest neighbor graphs. *Nature Biotechnology*, 40(2):245–253, 2022. doi: 10.1038/s41587-021-01033-z.

L. Haghverdi, M. Büttner, F. A. Wolf, F. Buettner, and F. J. Theis. Diffusion pseudotime robustly reconstructs lineage branching. *Nature Methods*, 13(10):845–848, 2016. doi: 10.1038/nmeth.3971. URL `https://doi.org/10.1038/nmeth.3971`.

M. Hu, S. Alkhairy, I. Lee, R. T. Pillich, D. Fong, K. Smith, R. Bachelder, T. Ideker, and D. Pratt. Evaluation of large language models for discovery of gene set function. *Nature Methods*, 22(1):82–91, 2025. doi: 10.1038/s41592-024-02525-x.

D. Kotliar, A. Veres, M. A. Nagy, I. Tirosh, and et al. Identifying gene expression programs of cell-type identity and cellular activity with cnmf. *Cell Systems*, 8(5):433–446.e5, 2019. doi: 10.1016/j.cels.2019.03.003.

D. D. Lee and H. S. Seung. Learning the parts of objects by non-negative matrix factorization. *Nature*, 401(6755):788–791, 1999. doi: 10.1038/44565.

H. M. Levitin, J. Yuan, Y. Cheng, and et al. De novo gene signature identification from single-cell rna-seq with hierarchical poisson factorization. *Molecular Systems Biology*, 15(2):e8557, 2019. doi: 10.15252/msb.20188557.

T. L. J. Lewis, J. Courchet, and F. Polleux. Cell biology in neuroscience: Cellular and molecular mechanisms underlying axon formation, growth, and branching. *The Journal of Cell Biology*, 202(6):837–848, 2013. doi: 10.1083/jcb.201305098.

M. D. Luecken and F. J. Theis. Current best practices in single-cell RNA-seq analysis: a tutorial. *Molecular Systems Biology*, 15(6):e8746, 2019. doi: 10.15252/msb.20188746.

R. C. Malenka and M. F. Bear. Ltp and ltd: An embarrassment of riches. *Neuron*, 44(1):5–21, 2004. doi: 10.1016/j.neuron.2004.09.012.

S. Persad, Z.-N. Choo, C. Dien, N. Sohail, I. Masilionis, R. Chaligne, T. Nawy, C. C. Brown, R. Sharma, I. Pe'er, M. Setty, and D. Pe'er. Seacells infers transcriptional and epigenomic cellular states from single-cell genomics data. *Nature Biotechnology*, 41(12):1746–1757, 2023. doi: 10.1038/s41587-023-01716-9.

R. Qu, X. Cheng, E. Sefik, et al. Gene trajectory inference for single-cell data by optimal transport metrics. *Nature Biotechnology*, 43:258–268, 2025. doi: 10.1038/s41587-024-02186-3.

L. T. Roumenina, M. V. Daugan, T. Noél, and et al. Context-dependent roles of complement in cancer. *Nat Rev Cancer*, 19 (12):698–715, 2019. doi: 10.1038/s41568-019-0210-0.

G. L. Semenza. Hypoxia-inducible factors in physiology and medicine. *Cell*, 148(3):399–408, 2012. doi: 10.1016/j.cell.2012.01.021.

A. Srivastava and C. Sutton. Autoencoding variational inference for topic models. *International Conference on Learning Representations (ICLR)*, 2017.

T. Stuart, A. Butler, P. Hoffman, C. Hafemeister, E. Papalexi, W. M. Mauck, Y. Hao, M. Stoeckius, P. Smibert, and R. Satija. Comprehensive integration of single-cell data. *Cell*, 177(7):1888–1902.e21, 2019. doi: 10.1016/j.cell.2019.05.031.

T. C. Südhof. Neurotransmitter release: The last millisecond in the life of a synaptic vesicle. *Neuron*, 80(3):675–690, 2013. doi: 10.1016/j.neuron.2013.10.022.

B. Tasic et al. Shared and distinct transcriptomic cell types across neocortical areas. *Nature*, 563:72–78, 2018. doi: 10.1038/s41586-018-0654-5.

D. van Dijk, R. Sharma, J. Nainys, K. Yim, P. Kathail, A. J. Carr, C. Burdziak, K. R. Moon, C. L. Chaffer, D. Pattabiraman, B. Bierie, L. Mazutis, G. Wolf, S. Krishnaswamy, and D. Pe'er. Recovering gene interactions from single-cell data using data diffusion. *Cell*, 174(3):716–729.e27, 2018. ISSN 0092-8674. doi: 10.1016/j.cell.2018.05.061. URL `https://www.cell.com/cell/fulltext/S0092-8674(18)30724-4`. Epub 2018 Jun 28.

I. Virshup, S. Rybakov, F. J. Theis, P. Angerer, and F. A. Wolf. anndata: Access and store annotated data matrices. *Journal of Open Source Software*, 9(101):4371, 2024. doi: 10.21105/joss.04371.

J. Winkler, A. Abisoye-Ogunniyan, K. J. Metcalf, and V. M. Weaver. Concepts of extracellular matrix remodelling in tumour progression and metastasis. *Nat Commun*, 11:5120, 2020. doi: 10.1038/s41467-020-18794-x.

F. A. Wolf, P. Angerer, and F. J. Theis. Scanpy: large-scale single-cell gene expression data analysis. *Genome Biology*, 19(1):15, 2018. doi: 10.1186/s13059-017-1382-0.

X. Zhang et al. Global pleiotropy and modularity in gene regulatory networks. *Nature Communications*, 13:1234, 2022. doi: 10.1038/s41467-022-XXXXX-X.
